# Metabolic Understanding of the Genetic Dysregulation in the Tumor Microenvironment of Kidney Renal Clear Cell Carcinoma

**DOI:** 10.1155/2022/6085072

**Published:** 2022-01-20

**Authors:** Junwei Xie, Lingang Cui, Shaokang Pan, Dongwei Liu, Fengxun Liu, Zhangsuo Liu

**Affiliations:** ^1^Department of Nephrology, The First Affiliated Hospital of Zhengzhou University, Zhengzhou 450052, China; ^2^Research Institute of Nephrology, Zhengzhou University, Zhengzhou 450052, China; ^3^Research Center for Kidney Disease, Zhengzhou, 450052 Henan, China; ^4^Key Laboratory of Precision Diagnosis and Treatment for Chronic Kidney Disease in Henan Province, Zhengzhou 450052, China; ^5^Core Unit of National Clinical Medical Research Center of Kidney Disease, Zhengzhou 450052, China; ^6^Department of Urology, The First Affiliated Hospital of Zhengzhou University, Zhengzhou 450052, China

## Abstract

The metabolic dysregulation is a hallmark of cancers including KIRC, specifically caused by alterations in metabolic genes. Currently, a lack of consensus exists between metabolic signatures in the tumor microenvironment. Here, in this study, we observed the significant correlations of differentially expressed metabolic genes (DEmGs) between KIRC and the related normal samples. Briefly, we collected sets of metabolic genes through RNA-seq data of KIRC and normal tissues from TCGA, followed by the identification of KIRC-related DEmGs. Next, patients were classified into three clusters, and using WGCNA, we identified metabolic genes involved in the survival among different clusters. Furthermore, we investigated survival and clinical parameters along with immune infiltration in the clusters. At the same time, we constructed and validated a prediction model based on these DEmGs. These analyses revealed that the patients having high expression of DEmGs showed poor survival, while infiltration of less-immune cells was associated with the metastasis of KIRC. In the end, we identified NUDT1 as a hub gene as it showed significantly high expression in KIRC samples as well as associated with the survival and prognosis of the patients. Further analysis revealed the oncogenic role of NUDT1 in 786-O and ACHN cells. Thus, we conclude that NUDT1 could be a potential diagnostic and prognostic marker for KIRC.

## 1. Introduction

Kidney renal clear cell carcinoma (KIRC) is a type of renal cell carcinoma (RCC), considered as one of the common cancers which accounts for 70–80% of cases [1], ranked as the 16^th^ most common cause of cancer-related mortality worldwide [2]. Renal cell carcinoma (RCC) is the most common type of renal cancer and accounts for 90% of the kidney cancer cases [3]. Recently, bioinformatics-based identification of the potential markers in cancers is being widely used but only few reliable biomarkers of KIRC have been identified or most of the markers are so far not fully validated. Thus, bioinformatics analyses coupled with experimental validations are necessary to elucidate the potential mechanism of the biogenesis and progression of KIRC [4]. Deep exploration of the tumor microenvironment and the development of immunotherapy have made it possible to study the interaction between tumor and the immune system [5]. Yet, many of the identified biomarkers have not been studied for their effect on the cellular phenotype and respective underlying molecular mechanisms in the KIRC [6]. However, the prognosis of KIRC patients is poorly understood and the overall 5-year survival is less than 10% after first diagnosis [7, 8]. Generally, the normal tissues predominantly contain high levels of the antiangiogenic factors. The disturbance in the level of these factors may activate the proangiogenic factors which further increase the division of cells at an abnormal rate leading to tumor formation [9]. In this process, tumor cells express a high level of proangiogenic growth factor and this effect supports the development of new blood vessels in the tumors [10]; thus, the development of the tumor begins. Hence, many other factors are involved in the tumorigenesis, including infiltration of the immune cells and the expression of various metabolic genes. It has been well known that the presence of different types of the immune cells in the tumor microenvironment largely affects the tumor progression and immunotherapy; thus, alterations of immune-related genes also affect the proportion and function of immune cells in the tumor microenvironment [11]. For example, the infiltration of immune cells in the tumor microenvironment significantly affects the development of glioma [12]. Despite the lacking interest in metabolic genes that influence cancers, the current studies have renewed that awareness of cancer as a metabolic disorder [13, 14]. These studies provide the base for including metabolic reprogramming as a new hallmark of malignant transformation [15]. However, the expression of all metabolic genes or pathways among different tissues including tumors and normal tissues differs from each other and it is largely unexplored especially in KIRC.

Overall, there is a smaller number of reliable biomarkers available for the prediction of prognosis and immunotherapeutic responses in the KIRC; thus, it is hard to have a complete clinical success. Therefore, comprehensive bioinformatics studies coupled with experimental validations could elucidate the potential mechanisms of the biogenesis and progression of KIRC. Thus, the current study is aimed at comprehensively assessing the effects of the metabolic pathways on the metabolic transcriptional profiles in KIRC compared with their matched normal tissues. Here, we successfully not only segregated the different disease sites or different molecular subtypes of the same disease but also predicted the response to metabolism-targeted therapy. This represents a new way of identifying a mechanism by which metabolic pathways are disturbed in the malignancy and offers novel targets for clinical interventions.

## 2. Material and Methods

### 2.1. Data Acquisitions

The RNA sequencing data (FPKM) relevant to KIRC were directly downloaded from The Cancer Genome Atlas (TCGA). Subsequently, the data were processed according to desired downstream applications. In addition, clinicopathologic data for the corresponding KIRC patients, including gender, race, age, tumor location, histology classification, differentiation grade, tumor stage, and survival information, were obtained from UCSC Cancer Browser. To gain insights of metabolic heterogeneity of KIRC patients, the metabolic genes' sets were taken from previous publications [16, 17]. The graphical abstract of this study is shown in supplementary Figure 1, and the detailed work flow of data acquisition and downstream process study is shown in supplementary Figure 2.

### 2.2. Identification of the Differentially Expressed Metabolic Genes (DEmGs)

The sets of the metabolic genes were identified by intersections of two datasets and used for DEG analysis between tumor and normal samples. Later, we also identified DEmGs using WGCNA, and based on survival modes, patients classified them into three clusters. In short, the patients were divided into three clusters, namely, cluster C1, cluster C2, and cluster C3. To identify DEGs, linear models were used by employing R package limma. A false discovery rate (FDR) adjusted *p* value < 0.05 combined with a simultaneous absolute value of log2 fold change, logFC > 1, and logFC < −1 were set as the threshold for DEG identification. The volcano plots were made using ggplot2. The genes with logFC > 1 were considered upregulated, and those with logFC < −1 were assigned as downregulated genes.

### 2.3. Functional Enrichment Analyses and Protein-Protein Interaction

The enrichment analyses by GO and KEGG terms were performed through the R package Cluster profile. The GO terms were divided into three categories including molecular functions (MF), cellular components (CC), and biological processes (BP). To determine the significant differences and correlations of DEmGs between tumor and normal tissues, we used computational software GSEA (gene set enrichment analysis). KEGG and GO (Gene Ontology) enrichment analyses of the DEGs were performed to identify potential pathways and functions. Furthermore, the cystoscope application was used to construct the protein-protein interaction (PPI) network of DEmGs. The initial PPI was obtained from the STRING database. The highest confidence limit was set to 0.9.

### 2.4. Survival Analysis

The differentially expressed genes in renal cancer patients were identified through the limma package, and the differentially expressed genes were analyzed by using WGCNA package to perform coexpression, to construct a coexpression network which identified a total of 6 modules. *β* = 5 and the network is a scale-free network. The prognostic significance of DEmGs identified by WGCNA in survival mode was determined by Kaplan-Meier (KM) plots with the logrank test.

### 2.5. Immune Infiltration Analysis

The immune and stromal cells were calculated using the R package, “xCELL; ” samples with *p* < 0.05 were selected for further analysis. Furthermore, the immune cells containing biological markers were analyzed by the R package GSVA. Moreover, to analyze the purity of the tumor, we used ESTIMATE algorithm to estimate the stromal and immune scores of a series of KIRC tissues.

### 2.6. Construction of the Prediction Model

The differentially expressed metabolic genes were obtained from RNA sequencing data of KIRC tumors with survival information, and the patients were randomly divided into training and testing cohorts using the R package “caret;” then, univariate survival analysis was performed on the DEmGs. Furthermore, LASSO analysis was performed by R package “glmnet;” and for optimization of the prediction model, a stepwise proportional hazards model was used.

### 2.7. Cell Culture

The human renal cancer cell lines 786-O and ACHN were purchased from the American Type Culture Collection (ATCC) (Manassas, VA, USA). The 786-O cell lines were cultured in RPMI 1640 medium (cat. no. 11875093, Invitrogen, Carlsbad, CA, United States) supplemented with 10% fetal bovine serum (cat. no. 04-001-1ACS, Biological Industries, Israel). The ACHN cell lines were cultured in MEM medium (cat. no. 41500034, Invitrogen, Carlsbad, CA, United States) supplemented with 10% fetal bovine serum. All cultures were incubated at 37°C and 5% CO_2_ in a humidified incubator.

### 2.8. Real-Time Quantitative PCR

Total RNA from the KIRC cell lines and tissues was isolated by using TRIzol (cat. no. 9109; TaKaRa, Tokyo, Japan) according to the manufacturer's protocols. RT-qPCR was performed using QuantiNova SYBR Green PCR Kit (cat. no. 208054, QIAGEN, Duesseldorf, Germany). Relative gene expression was calculated by the 2^−ΔΔ*Ct*^ method. The primer sequences are listed in Supplementary Table 1.

### 2.9. Cell Viability Assay

The cytotoxicity after NUDT1 siRNA transfection in 786-O and ACHN was determined using a Cell Counting Kit-8 (CCK-8) assay (cat. no. C0038, Beyotime Biotechnology Co. Ltd., China). The NUDT1 siRNA sequences are provided in Supplementary Table 1. They were synthesized from GenePharma (Shanghai, China). Briefly, both cell lines 786-O and ACHN in the logarithmic growth phase (24 h after siRNA transfection) were collected and dispensed into 96-well cell culture plates (1000 cells/well). The following day, 10 *μ*l of CCK-8 was added to each well. After incubation at 37°C for a further 2 h, the optical density (OD) at 450 nm of cells was detected via a microplate reader (Bio-Rad Laboratories, Richmond, CA, USA).

### 2.10. Cell Migration, Invasion, and Apoptosis Assay

KIRC cells 786-O and ACHN were transfected for siRNA-mediated knockdown of the NUDT1. After 24 h of the transfection, cells were seeded into the 6-well plates for wound healing assay. After cells obtained 100% confluence, the wounds were generated in the cells as a monolayer using a plastic pipette tip. The cells were then rinsed with PBS and cultured for another 48 h. The distance of wound closure was visualized by a microscope and photographed for measuring the effect of NUDT1 knockdown on KIRC 786-O and ACHN cells. Cell migration & invasion were performed using transwell 24-well plates (Corning, New York, NY). In short, an equal number of the cells were cultured in the FBS-free media in the upper chamber and migration of the cells was observed in the lower chamber of the transwell where media was supplemented with 10% FBS. Transwell chambers were carefully washed and stained with 0.2% crystal violet, visualized, and photographed; later on, differences between migrated cells between negative control and NUDT1 knockdown were determined by simply cell counting. Apoptosis assay was performed after 72 h of the transfection, cells were harvested, washed, and resuspended in ice-cold PBS. Cells were then detected by the Apoptosis Detection Kit (cat. no. KGA1013, KeyGen BioTech, Nanjing, China) according to the manufacturer's instructions and examined by flow cytometry (FACScan; BD Biosciences). All experiments were performed in triplicate.

### 2.11. Statistical Analysis

All the statistical analyses were performed at GraphPad prism (V7.0) and R (3.6.4), the Wilcox test was used to compare the infiltration of immune cells in normal and tumor tissues, and the chi-square test was performed for studying the correlation between immune cell infiltration and pathological parameters. Analysis of the variance was used to compare the immune score, stromal score, and tumor purity among the three clusters. For the survival analysis, the *p* value was calculated using the logrank test. A *p* value of <0.05 was considered statistically significant.

## 3. Results

### 3.1. Identification of Differentially Expressed Metabolic Genes in KIRC

**Figure 1:**
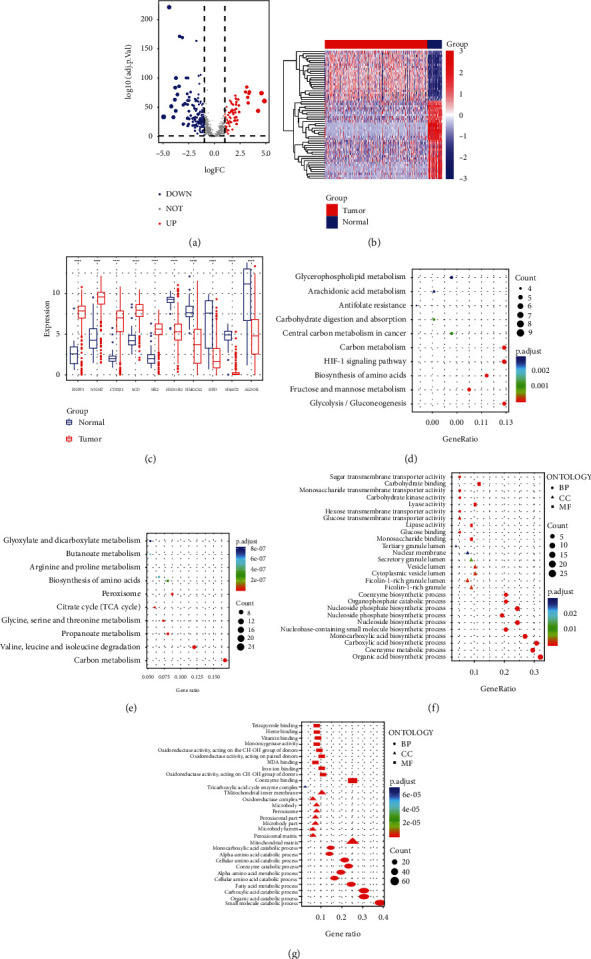
Differentially expressed metabolic genes in KIRC tumors. (a, b) Volcano and heat map showing the differently expressed metabolic genes (DEMGs) between tumor and normal tissues. (c) Boxplot showing top10 DEGs. (d, e) KEGG enrichment analysis of up- and downregulated genes. (f, g) GO enrichment analysis of up- and downregulated genes.

In order to explore the metabolic dysregulation in KIRC, we explored the available TCGA data to get deep insights for metabolism-targeted therapeutics in the clinic. For this purpose, we selected a set of 1916 metabolic genes that were intersected from two different datasets [16, 17] and screened out the 1100 differentially expressed genes in tumor vs normal tissues (Supplementary Table 2). These differentially expressed metabolic genes were plotted in volcanoes and heat maps (Figures 1(a) and 1(b)). Out of 1100 differentially expressed metabolic genes, 78 genes were upregulated and 163 genes were downregulated. Moreover, there were 859 genes were unchanged. The heat map represents the individual expression index of those differential metabolic genes in tumor and normal samples (Figure 1(b)). Next, we identified the top 10 differentially expressed metabolic genes; among them, ENPP3, NNMT, CYP2J2, SCD, and HK2 were upregulated and HSD11B2, HMGCS2, HPD, HS6ST2, and ALDOB were downregulated. The box plots of these DEmGs are shown in Figure 1(c). Among the upregulated genes, ENPP3 is ~7-fold expressed in tumors. Alternatively, the gene ALDOB is ~5-fold downregulated in analyzed tumor samples.

In addition, we evaluated KEGG pathway and GO analyses of DEmGs. KEGG pathway analysis revealed that the upregulated genes were significantly enriched in carbon metabolism, HIF1 signaling, and glycolysis/gluconeogenesis with a higher gene ratio (8–9 number of genes in each pathway) (Figure 1(d)). Similarly, among downregulated DEmGs, we found that carbon metabolism and valine, leucine, and isoleucine degradation were the top pathways affected by metabolically active genes (Figure 1(e)). The pathways related to peroxisome organelle were also significantly enriched in a downregulated group of gene tumor samples. Noteworthy, compared with those in the upregulated genes, the pathways involved in downregulated genes have higher significant *p* values. It is worth mentioning that most of the KEGG pathways enriched in downregulated gene categories were related to amino acid metabolism. To further dissect the involvement of DEmG in tumorigenesis, the GO functional analyses of upregulated and downregulated genes were performed. We divided the GO ontology in three functional subontology groups, BP (biological process), CC (cellular component), and MF (molecular function) (Figures 1(f) and 1(g)). In addition, GSEA analysis revealed a significant increase in enrichment of genes associated with BENPORATH_MYC_TARGETS_WITH_EBOX in tumors (Supplementary Figure 3(a)), while BROWN_MYELOID_CELL_DEVELOPMENT_UP, KEGG_ALPHA_LINOLENIC_ACID_METABOLISM and KEGG_ETHER_LIPID_METABOLISM were found to be negatively enriched (Supplementary Figures 3(b)-3(d)). In the next stage, we constructed a protein-protein interaction PPI network with up- and downregulated DEmGs. A number of the genes showed interaction with each other. Through these genes' interactions, we isolated hub genes. As shown in supplementary Figure 4(a), each node is discrete from the other based on the degree value; further, we isolated the top 7 hub genes for PPI (Supplementary Figure 4(b)). We also explored the correlation between these hub gene expression and clinicopathological features of KIRC in TCGA datasets (Supplementary Table 3).

### 3.2. Network Analysis Reveals Basic Metabolic Changes in Various Tumor Ontologies

**Figure 2:**
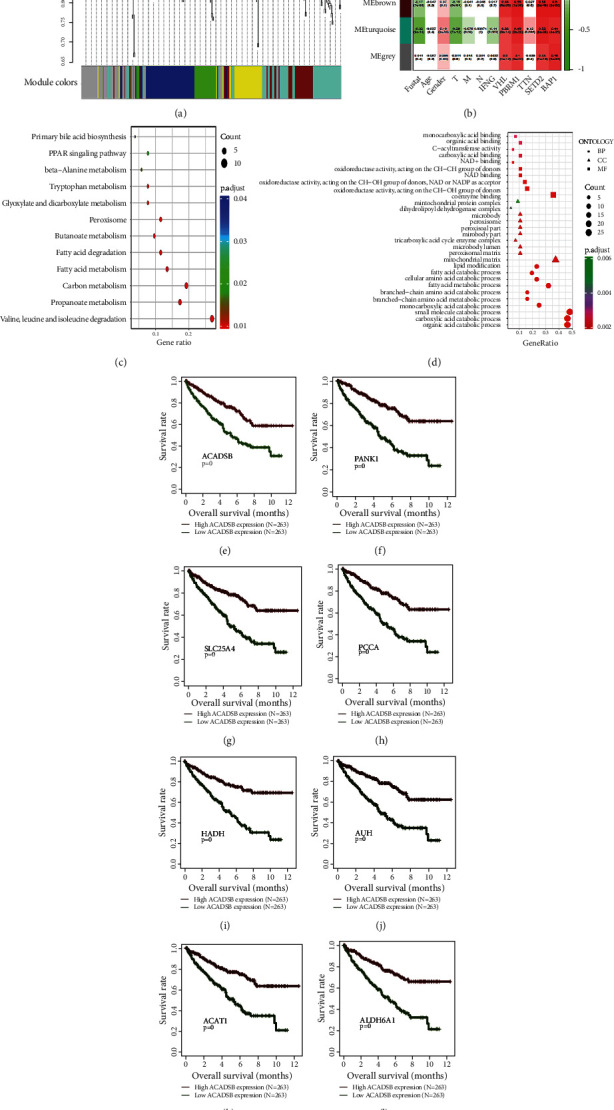
Enrichment analyses of the differentially expressed metabolic genes. (a) Cluster tree of WGCNA. (b) Heat map showing the correlation between the module and clinical parameters. (c, d) KEGG and GO enrichment analyses of genes in the survival module. (e–l) Survival analysis of top metabolic genes in KIRC.

Next, the expression data of DEmGs were selected and used as the input data for WGCNA, which identified 6 distinct coexpression modules containing a different number of genes for each module (Figure 2(a)). We correlated differential genes with external traits and identified the modules that were significantly associated with clinical traits (Figure 2(b)). Based on the correlation coefficient, we found that MEturquoise modules were negatively correlated with the survival status. GO and KEGG pathway enrichment analyses were performed using genes from these modules (Figures 2(c) and 2(d)). The most enriched KEGG pathways were valine, leucine, and isoleucine degradation; carbon metabolism; propanoate metabolism; fatty acid metabolism; fatty acid degradation; peroxisome and butanoate metabolism; glyoxylate and dicarboxylate metabolism; and tryptophan metabolism (Figure 2(c)). The genes related to BP terms were predominantly enriched in small molecule, carboxylic acid, and organic acid catabolic processes. The genes related to CC terms were mainly enriched in the mitochondrial matrix. The differentially expressed genes related to MF were mainly enriched in coenzyme binding (Figure 2(d)). In addition, we performed a survival analysis of 8 genes in the survival module. Patients with higher ACADSB, PANK1, SLC25A4, PCCA, HADH, AUH, ACAT1, and ALDH6A1 expression had a longer survival rate than those with lower expression of these genes (*p* = 0) (Figures 2(e)–2(l)).

### 3.3. Clustering of the KIRC Patients

**Figure 3:**
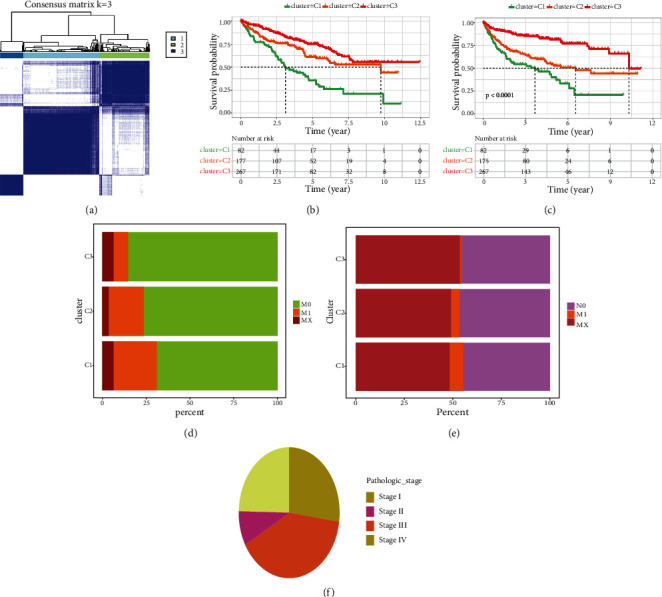
Clustering of the KIRC patients based on differentially expressed metabolic genes. (a) Heat map showing clusters of KIRC based on the expression of genes in MEturquoise module. (b, c) OS and DFS of three clusters. (d, e) Difference of clinical parameters of three clusters. (f) Pathological stages of KIRC according to different clusters.

We selected top DEmGs for cluster analysis; the KIRC patients were grouped into three clusters based on the differential expression of metabolic genes.  Figure 3(a) shows the heat maps of DEmGs in the KIRC patients. The color scale indicates the expression value (light-blue indicates lower expression value; darker-blue indicates higher gene expression values).

KM curves were plotted to compare the overall survival of the three clusters for KIRC patients. The overall survival rates differed significantly across the three clusters (*p* < 0.01Figure 3(b)). Cluster 1 showed a worse survival rate compared with cluster 2 and cluster 3. The PFS survival rate also differed significantly among the 3 clusters (*p* < 0.001, Figure 3(c)), and cluster 1 exhibited a worse PFS survival rate compared with cluster 2 and cluster 3.

Different colors in our model represent clinical parameters and underlying pathological stages (Figure 3(d)). Cluster 3 has lower Mo ratios and higher M1 value as compared with clusters 1 and 2 suggesting higher cancer metastasis and more advanced stage of tumors in cluster 3 than clusters 1 and 2. Similarly, in cluster 3, cancer has spread more to the lymph nodes (higher N1) as compared with those in clusters 1 and 2. Most of the KIRC patients were diagnosed at stages III and IV (Figures 3(e) and 3(f)), suggesting larger or expanded tumors, as well as moving through the blood or lymphatic system to a distant region in the body.

### 3.4. Immune Status of Three Clusters

**Figure 4:**
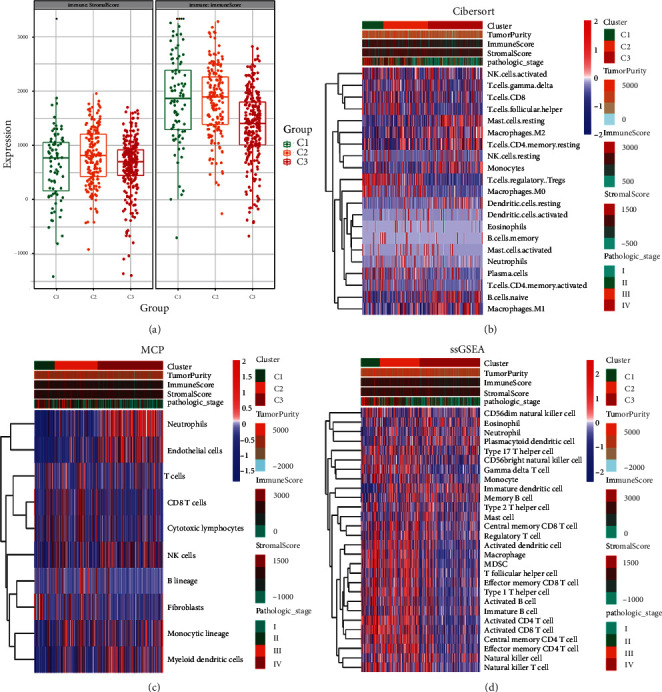
Immune cell infiltration in KIRC tumors. (a) Immune and stromal score of three clusters. (b–d) Infiltration of immune cells in three clusters analyzed using CIBERSORT, MCP-counter, and ssGSEA.

We used the ESTIMATE algorithm to estimate the stromal and immune scores of a series of KIRC tissues based on their metabolic transcriptional profiles (Figure 4(a)). Later, these scores were taken into account to develop a stromal-immune score-based metabolic genes signature for prognosis stratification in KIRC. As shown in Figure 4(a), three cluster groups (C1, C2, and C3) were stratified in box plots based on their stromal-immune score. Among three clusters, C1 showed the higher significant score in both stromal and immune classifications.

Furthermore, three clusters were analyzed by CIBERSORT with a *p* value < 0.1 (Figure 4(b)). The tumor purity, immune score, and stromal score along with the pathological stages of 3 clusters are shown at the top of heat map. In this analysis, we majorly found that regulatory T cells (Tregs) were enriched in cluster C1 and patients in C1 were mainly at pathological stages III and IV. Moreover, the activated NK cells, CD8+ T cells, T follicular helper cells, and M0 macrophages in the C1 cluster; CD8+ T cells and T follicular helper cells in the C2 cluster; and resting mast cells, M2 macrophages, resting memory CD4 T cells, monocytes, naive B cells, and M1 macrophages in the C3 cluster were also detected (Figure 4(b)).

Apart from CIBERSORT, we employed other algorithmic packages to check the status of immune infiltration. The hierarchical heat map of MCP analysis is shown in Figure 4(c). The key findings of MCP analysis were neutrophil infiltration and endothelial cell infiltration in cluster C3 that were missing in cluster C1. This analysis also revealed NK cell, monocytic lineage, and myeloid dendritic cell infiltration in cluster C3. Other immune cell populations were mixed in three analyzed clusters (Figure 4(c)).

To complement CIBERSORT and MCP analyses, we applied ssGSEA to quantify infiltration levels for immune cell types implemented in R package GSVA. Three clusters' data were fed to ssGSEA package and obtained richness of 28 immune-related cells and types in KIRC samples. Results revealed that C1 and C2 had more immune infiltration; some innate immune cells, including NK, neutrophils, and eosinophil, were mixed in 3 clusters (Figure 4(d)).

### 3.5. Construction and Validation of the Predicting Model Based on DEmGs

**Figure 5:**
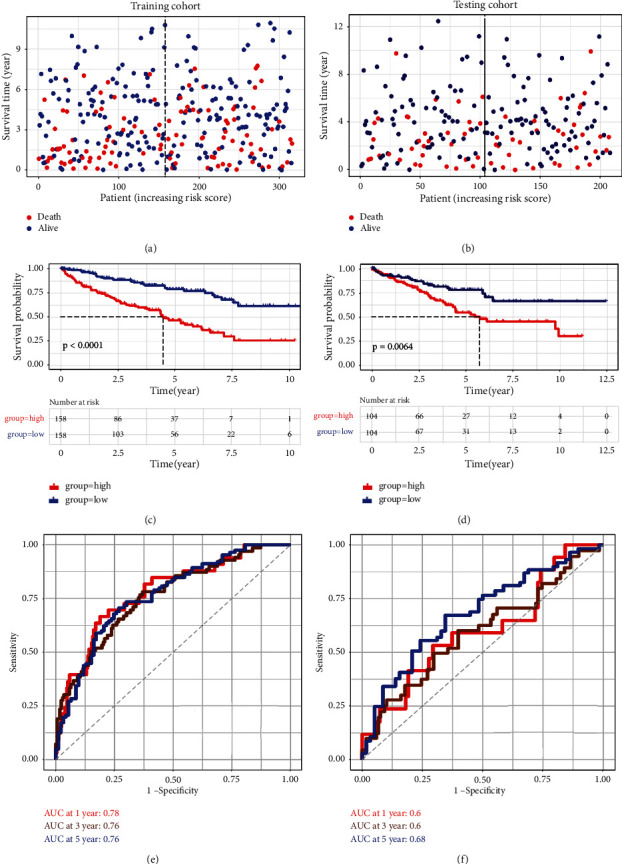
Construction and validation of the prediction model. (a, b) Dot plot showing the correlation of the risk score in training and testing cohort. (c, d) Survival analysis of high- and low-risk groups in training and testing cohorts. (e, f) ROC analysis of 1, 3, and 5 years in training and testing cohort.

Lastly, we constructed and validated the prediction model based on the differential expression of the metabolic genes. We calculated the immune-related risk score of DEmGs based on overall survival. For this purpose, we devised two groups for evaluating the correlation of the risk score; one is for the training cohort and the other for the testing cohort. We found that overall survival was low and scattered across the risk score (Figures 5(a) and 5(b)). Next, based on the median risk score, we assigned KIRC patients into high- and low-risk groups for further evaluation. We then performed survival analysis of these two risk groups in training and testing cohorts. As expected, the high-risk groups were found to have a low survival as compared with the low-risk groups (Figures 5(c) and 5(d)). Furthermore, ROC curve analysis was performed for training and testing cohorts. We observed a ROC score of 0.68 at 5 years in testing cohorts, which indicates a good performance in predicting the prognosis of KIRC (Figures 5(e) and 5(f)). In addition to the ROC curve analysis, we also executed the LASSO COX regression model to validate our prognostic model as indicated by partial likelihood deviance (Supplementary Figure 5(a)) and regression coefficient of the DEmGs (Supplementary Figure 5(b)). Lastly, five genes (ABCG1, CRYL1, FDX1, PANK1, and SLC44A) were predicted to be potential prognostic factors with HR < 1 (Supplementary Figure 5(c)).

### 3.6. Underlying Mechanisms of the KIRC Progression

**Figure 6:**
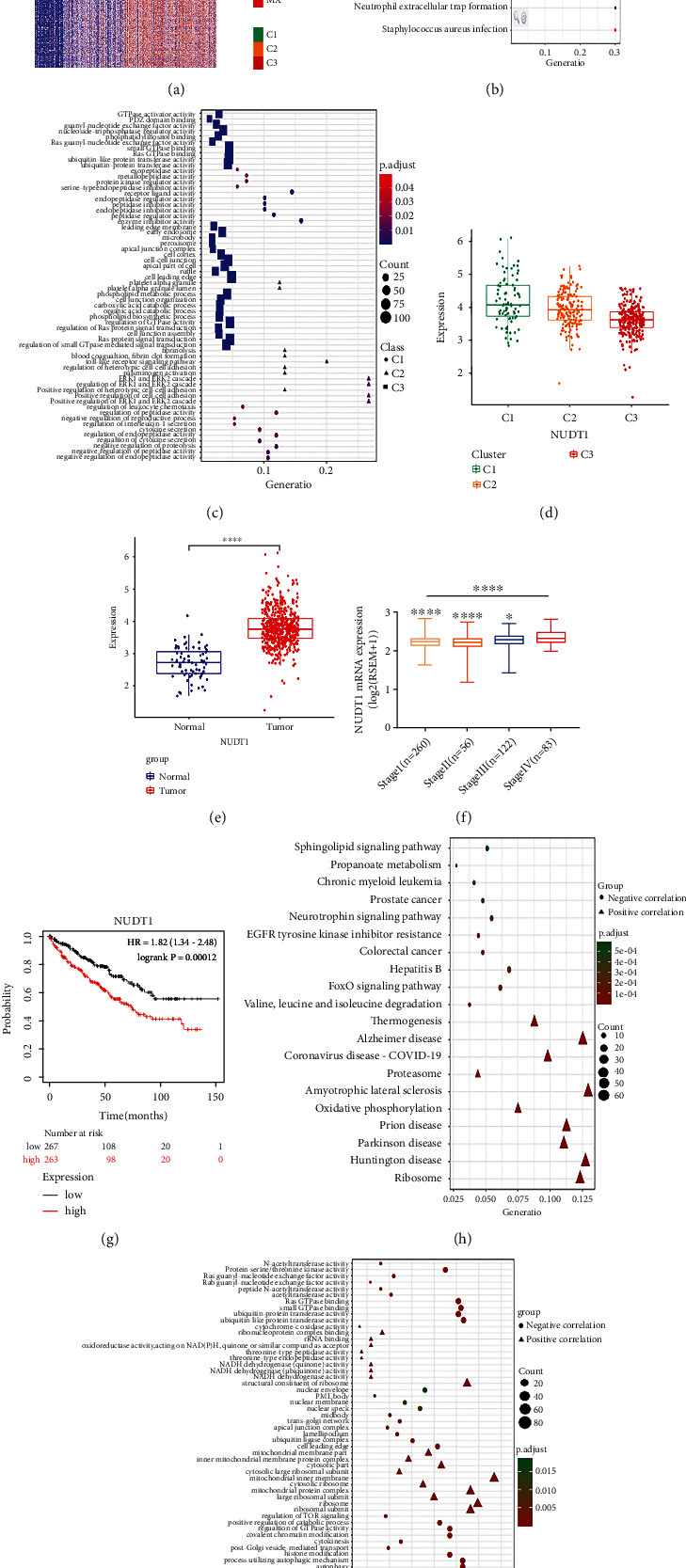
Genes involved in KIRC progression and underlying mechanisms. (a) Heat map showing the DEGs among three clusters. (b, c) KEGG and GO enrichment analyses of DEGs among three clusters. (d) NUDT1 expression in three clusters. (e) NUDT1 expression in normal and tumor tissues. (f) NUDT1 expression during stages I–IV. (g) Survival analysis of NUDT1 in KIRC. (h, i) KEGG and GO enrichment analyses of NUDT1-related genes.

To investigate further the underlying mechanism for KIRC progression, we conducted differential expression analysis among all clusters and utilized a heat map plot to visualize the results (Figure 6(a)). To identify the signaling pathways of the DEmGs, we performed KEGG and GO enrichment analyses of the DEGs in three clusters. In short, these results revealed that DEGs of three clusters were mainly enriched in focal adhesion, the Foxo signaling pathway, and the Apelin signaling pathway for cluster C3 and mineral absorption, neutrophil extracellular trap formation, and staphylococcus aureus infection for cluster C2 (Figure 6(b)). In addition, GO functional analysis of DEGs uncovered MF-, CC-, and BP-related ontologies shown in Figure 6(c). Interestingly, the differential expression analysis disclosed abnormal behavior of genes' regulation in three clusters. Mostly, NUDT1 was highly expressed in C1, which had the worst survival. Further investigation revealed that NUDT1 expression was significantly downregulated through the progression from C1 to C3 (Figure 6(d)). Moreover, the NUDT1 was found to be upregulated in KIRC tumor samples (Figure 6(e)). Next, we highlighted the NUDT1 expression in each tumor's stages (Figure 6(f)). Overall survival analysis was also performed by the Kaplan-Meier plotter, and we found that patients with higher expression of NUDT1 had worse overall survival (HR = 1.82 (1.34–2.48), logrank *p* = 0.00012) (Figure 6(g)).

Lastly, we performed KEGG and GO functional enrichment analyses for genes interacting with NUDT1. The genes were divided into two groups—positively correlated with NUDT1 and negatively correlated with NUDT1. KEGG pathway analysis showed that positively correlated genes were mainly enriched in the ribosomal pathway, Huntington disease, amyotrophic lateral sclerosis, and Alzheimer's disease. On the other hand, the negatively correlated genes were enriched mainly in hepatitis B and Foxo signaling (Figure 6(h)). Moreover, the GO ontology of three different groups MF, CC, and BP for both positively and negatively correlated genes was shown in Figure 6(i). In addition, we found that the expression of NUDT1 was highly correlated with the infiltration of immune cells (Supplementary Figure 6) and different clinical features of the KIRC patients (Table 1).

### 3.7. Loss of NUDT1 Inhibits Renal Cancer Cell Proliferation and Migration

**Figure 7:**
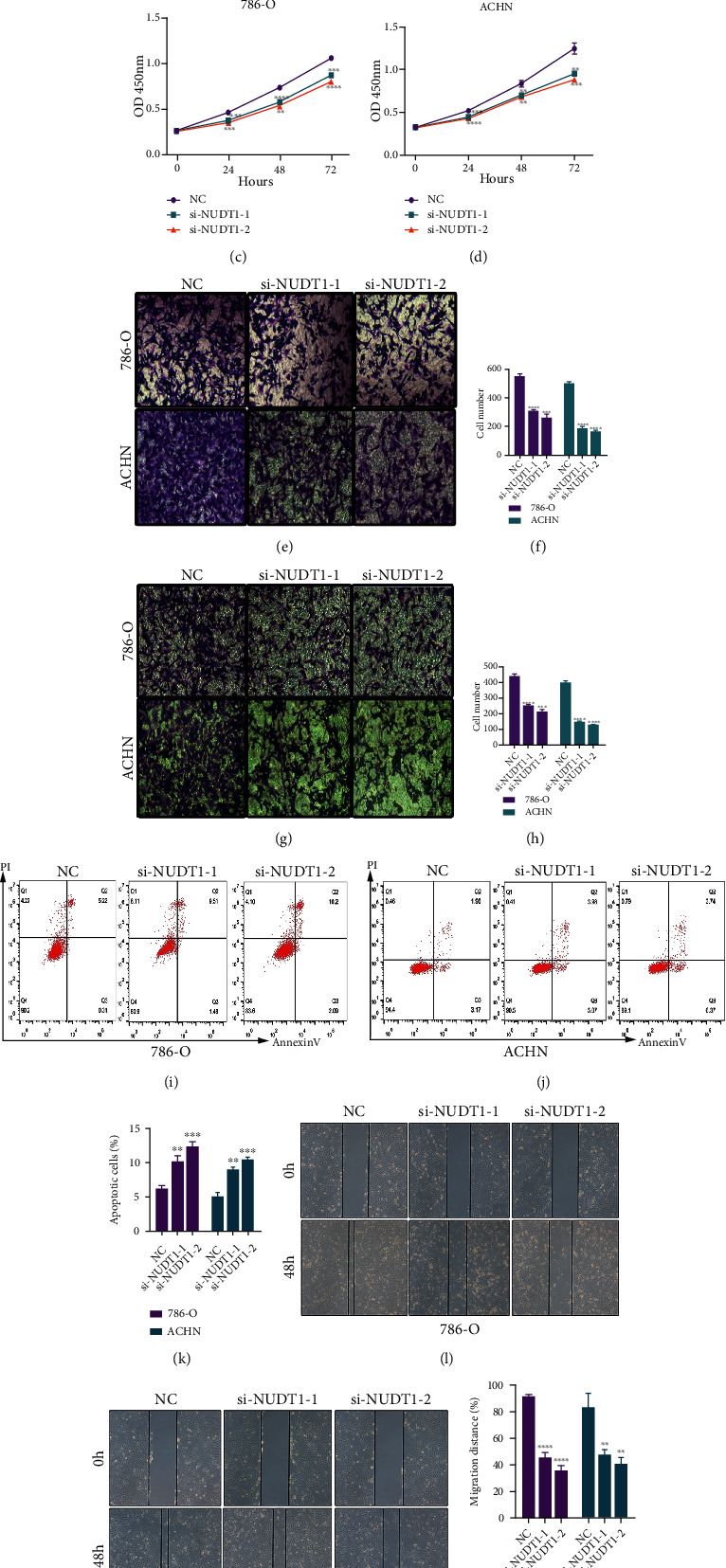
NUDT1 regulate renal cancer cell proliferation and migration. (a) NUDT1 relatively express higher in the KIRC tissues as compared to normal tissues. (b) siRNA-mediated knockdown of NUDTI in 786-O and ACHN cells. (c, d) Cell proliferation assay showing reduced proliferation of KIRC cells in siRNA-mediated silencing of the NUDTI cells. (e–h) Cell migration and cell invasion reduced in 786-O and ACHN cells after NUDTI knockdown. (i–k) Number of apoptotic cells significantly increased in knockdown cells. (l–n) Wound healing slows down in the NUDT1 knockdown cells.

Next, we compared the expression level of NUDTI in KIRC tissues and their associated normal tissues, which revealed that NUDT1 highly expressed in KIRC tissues (Figure 7(a)). Furthermore, we determined the effects of NUDT1 loss on renal cancer cell lines by using siRNA mediated inhibition. NUDT1 was targeted for siRNA knockdown in two cell lines 786-O and ACHN and the NUDT1 mRNA levels were successfully inhibited as evidenced by qPCR analysis (Figure 7(b)). Following the siRNA-mediated knockdown of NUDT1, cell viability assay showed reduced cell viability in both cell lines (Figures 7(c) and 7(d)). Afterwards, cell migration assay upon knockdown of NUDT1 showed significantly reduced cell migration in NUDT1-depleted 786-O and ACHN cells (Figures 7(e) and 7(f)). The migration capacity of 786-O cells was reduced to about 50%, and 70% reduction was observed in ACHN cells upon NUDT1 knockdown (Figure 7(f)). The cell invasion was also inhibited in both cell lines when the NUDT1 gene was knocked down (Figures 7(g) and 7(h)). To complement the migration, we also performed wound healing assay when NUDT1 was depleted from 786-O and ACHN cell lines and we observed similar results of reduced wound healing capability in both cell lines lacking NUDT1 (Figures 7(l)–7(n)). Based on these results, we hypothesized that loss of NUDT1 could lead to apoptosis in renal cancer cells. Therefore, we measured the percentage of the apoptotic cells upon silencing of NUDT1. Interestingly, we discovered that the percentage of apoptotic cells was significantly increased in NUDT1-depleted cells (Figures 7(i)–7(k)).

## 4. Discussion

Kidney renal clear cell carcinoma (KIRC) is one of the top commonly occurring cancers worldwide, generally showing no early symptoms until the tumor becomes large enough; therefore, the mortality rate is relatively high [18–20]. Thus, it is necessary to investigate the carcinogenesis of KIRC and to identify useful biomarkers for its early diagnosis. However, limited knowledge so far is established about the pathogenesis and carcinogenesis of KIRC. In addition, not many molecular markers for clinical practice have been validated. Advanced high-throughput sequencing and bioinformatics technology make it possible to select effective biomarkers [21]. The RNA sequencing data and clinical annotations of over five hundred KIRC cases are freely available on the TCGA database. Taking advantage of this freely available data from TCGA, we analyzed RNA sequence data for differentially expressed metabolic genes in tumor vs normal tissue samples. Among upregulated and downregulated genes, we identified the top 10 differentially expressed metabolic genes (DEmGs). We believe that metabolic genes have diverse functions in KIRC; still, finding suitable diagnostic and therapeutic markers could be a challenge from the pool of genes with diversified functions.

Previously, studies have estimated immune cell infiltration in the tumor microenvironment of several cancers. A relationship between tumor immune cells and angiogenesis in KIRC samples' data obtained from TCGA was studied, and RFX2, SOX13, and THRA were identified as the top three MTF in regulating angiogenesis signature in KIRC patients [4]. Moreover, two independent m6A modification patterns control biological functions, immunological characteristics, and prognoses of KIRC [22]. Autophagy-related protein 5 (ATG5) has been linked with the progression of several cancers including KIRC [23]. In the current analysis, some of the differentially expressed genes including PBRM1, SET2, VHL, and BAP1 showed significant correlation with metabolic pathways in KIRC data. For further deep investigation, we clustered the patients based on the DEmGs; cluster 1 showed a worse overall survival rate as compared with the other clusters; anyhow, the KIRC patients in cluster 3 have advance tumor stages and have high lymph nodes (higher N1) as compared with those in clusters 1 and 2, showing cancer metastasis and expansion of tumors in cluster 3. It shows a lesser number of metabolic genes in clusters associated with the cancer metastasis.

In addition, immune infiltration scores in different clusters show C1 with high scores in stromal and immune classifications. Noteworthy, C1 patients at pathological stages III and IV have high infiltration of T cells along with CD8+ T cells, T follicular helper cells, and macrophages. While also have abundant Tregs. The Tregs have a vital role in the immune tolerance and homeostasis [24]. In many cancers such as colon cancer, breast cancer, and pancreas cancer, the increased percentage of the Tregs is associated with the poor prognosis of cancer [25, 26]. The M0 macrophage induces the invasion and proliferation of cells [27], and elevated levels of the macrophages are associated with poor prognosis in RCC [28]. Likewise, CD8+ T cells are known as the key antitumor cells and top choice of the targeted immune cell therapy for the cancers [29]. Although C1 has a highest infiltration of CD8+ T cells than other clusters, it had the worst overall survival.

We used three methods CIBERSORT, MCP, and ssGSEA to study the immune cell infiltration in the KIRC tumor microenvironment. The traditional method to measure the tumor immune infiltration is through histology on tissue sections and immune subsets inferred by immunohistochemistry of individual markers. However, there are several limitations where immunohistochemistry cannot identify many immune populations and performs poorly at capturing functional phenotypes (e.g., activated vs. resting lymphocytes). Therefore, we utilized CIBERSORT, a computational approach developed by [30] that addresses the challenges faced by immunohistochemistry. Apart from CIBERSORT, we employed other algorithmic packages to check the status of immune infiltration. This is because CIBERSORT measures only intrasample proportions of immune cell populations that can be resolved by another package such as the MCP-counter which can estimate the cells' population in abundance that enables an intersample comparison of infiltrating cells in the tumor microenvironment [31]. To complement CIBERSORT and MCP analyses, we applied ssGSEA to quantify infiltration levels for immune cell types implemented in R package GSVA [32, 33]. The ssGSEA is a rank-based method that computes an overexpression for a genes' list of interest relative to all other genes in the genome. The CIBERSORT showed better results as compared to the other two methods; thus, further analyses were performed from data obtained from CIBERSORT.

Moreover, we focused on the underlying mechanism of the KIRC progression by differential expression analysis based on the RNA-seq data. Overall, we targeted abnormal differential expression of the common genes among three clusters. Most of the genes were downregulated except NUDT1 in C1; however, its expression significantly downregulated through the progression from C1 to C3. Thus, NUDT1 was further validated for its role in KIRC progression. The siRNA-mediated inhibition of NUDTI gene expression in two KIRC cell lines (786-O and ACHN) reduced the cell viability and cell migration and increased apoptosis, which confirm its role in tumor progression. Previously, it has been reported that the level of NUDT1 expression correlated with the tumor grade, stage, size, differentiation, degree of vascular invasion, overall survival (OS), and disease-free survival (DFS) in HCC patients, also predicted as a prognostic marker with therapeutic potentials in HCC patients [34]. Overexpressing NUDT1 in pulmonary arterial hypertension reduces the oxidative stress and DNA damage, hence promoting cell proliferation and reducing apoptosis [35]. It has been shown that patients with oral squamous cell carcinoma (OSCC) having high expression of NUDT1 have shown a poor survival rate [36]. Based on the fact that not enough literature is available about NUDTI's role in cancers and, so far, no study has ever reported its role in KIRC, therefore, we are reporting for the first time the role of NUDTI in KIRC progression. The current study had some limitations; although our research found that the signature might be associated with immunotherapy of KIRC, the efficacy of the signature could not be validated due to the lack of data, the potential underlying mechanisms, and functional roles of NUDT1 in KIRC and clinical practice need further exploration.

## 5. Conclusions

We screened dysregulated metabolic genes between normal and tumor tissues and explored their function. WGCNA analysis identified a group of genes correlated with the survival status of KIRC. Consensus clustering based on survival-related genes demonstrated three clusters with different survival rates and immune infiltration patterns. NUDT1 negatively correlated with survival, and further analyses revealed that knockdown of NUDT1 inhibits proliferation and migration of tumor cells. Of note, a prediction model was constructed based on survival-related genes, which showed high efficiency in predicting the survival of KIRC. In conclusion, we performed an exhaustive analysis of metabolic genes in KIRC and identified NUDT1 as oncogene which could be used as a therapeutic and prognostic target.

## Figures and Tables

**Table 1:** Correlation between NUDT1 expression and clinicopathological features of KIRC in TCGA datasets.

Characteristics	*n*	NUDT1 relative expression		*p* value
		Low (*n*)	High (*n*)	
*Age (years)*				
<65	329	169	160	0.137
≧65	197	88	109	
*Gender*				
Male	342	156	186	**0.042**
Female	184	101	83	
*T stage*				
0, 1, 2	269	147	122	**0.007**
3, 4	257	110	147	
*LN meta*				
With	16	3	13	**0.010**
Without	239	124	115	
*Distant metastases*				
With	78	27	51	**0.004**
Without	416	218	198	
*Stage*				
I, II	319	177	142	**<0.001**
III, IV	205	80	125	

## Data Availability

The RNA-seq data used in this study are available in The Cancer Genome Atlas repository (https://cancergenome.nih.gov), clinicopathologic data regarding KIRC patients are available at the UCSC Cancer Browser, and raw data or figures generated in the current study can be requested from the corresponding author upon reasonable request.

## Abbreviations

KIRC: Kidney renal clear cell carcinoma
RCC: Renal cell carcinoma
DEGs: Differentially expressed genes
DEmGs: Differentially expressed metabolic genes
TCGA: The Cancer Genome Atlas
UCSC: University of California Santa Cruz
KEGG: Kyoto Encyclopedia of Genes and Genomes
GO: Gene Ontology
MF: Molecular functions
CC: Cellular components
BP: Biological processes
GSEA: Gene set enrichment analysis
PPI: Protein-protein interaction
Tregs: Regulatory T cells
OS: Overall survival
DFS: Disease-free survival.

## Supplementary Materials

Supplementary Materials Supplementary Figure 1: graphical abstract of this study. Supplementary Figure 2: the detailed workflow of data acquisition and downstream process study. Supplementary Figure 3: GSEA enrichment analysis of metabolic genes in tumor and normal tissue. Supplementary Figure 4: PPI network of metabolic genes. (a) PPI network of up- and downregulated metabolic genes. (b) Top 10 hub genes of PPI. Supplementary Figure 5: screening the survival-related genes. (a) Partial likelihood deviance of DEGs identified by LASSO regression model. (b) LASSO coefficients of DEGs. (c) Forrest plot showing multivariate analysis of selected genes identified by LASSO regression model. Supplementary Figure 6: correlation of NUDTI expression with immune infiltration level in KIRC. Supplementary Table 1: primers used in qPCR analysis and the small RNA interfering assay. Supplementary Table 2: the 1100 differentially expressed metabolic genes in tumor vs normal tissues. Supplementary Table 3: correlation between hub gene expression and clinicopathological features of KIRC in TCGA datasets.
