# Supplementary material for: Metabolic Understanding of the Genetic Dysregulation in the Tumor Microenvironment of Kidney Renal Clear Cell Carcinoma
Source: Dis Markers. 2022 Jan 20;2022:6085072. doi: 10.1155/2022/6085072 (PMC8794690; doi:10.1155/2022/6085072)
Supplement: Supplementary Materials — Supplementary Figure 1: graphical abstract of this study. Supplementary Figure 2: the detailed workflow of data acquisition and downstream process study. Supplementary Figure 3: GSEA enrichment analysis of metabolic genes in tumor and normal tissue. Supplementary Figure 4: PPI network of metabolic genes. (a) PPI network of up- and downregulated metabolic genes. (b) Top 10 hub genes of PPI. Supplementary Figure 5: screening the survival-related genes. (a) Partial likelihood deviance of DEGs identified by LASSO regression model. (b) LASSO coefficients of DEGs. (c) Forrest plot showing multivariate analysis of selected genes identified by LASSO regression model. Supplementary Figure 6: correlation of NUDTI expression with immune infiltration level in KIRC. Supplementary Table 1: primers used in qPCR analysis and the small RNA interfering assay. Supplementary Table 2: the 1100 differentially expressed metabolic genes in tumor vs normal tissues. Supplementary Table 3: correlation between hub gene expression and clinicopathological features of KIRC in TCGA datasets. [file 6085072.f1.zip › Supplementary table 2.docx]

| **Supplementary table 2: The 1100 differentially expressed metabolic genes in tumor vs normal.** | | | | | | | |
| --- | --- | --- | --- | --- | --- | --- | --- |
| **logFC** | **AveExpr** | **t** | **P.Value** | **adj.P.Val** | **B** | **threshold** | |
| ENPP3 | 4.837965 | 6.69666 | 18.9384 | 4.65E-63 | 1.31E-61 | 132.555 | UP |
| NNMT | 4.550369 | 8.64553 | 21.57758 | 7.15E-77 | 3.75E-75 | 164.2882 | UP |
| CYP2J2 | 4.219104 | 5.805885 | 15.52555 | 6.03E-46 | 8.14E-45 | 93.2687 | UP |
| SCD | 3.37228 | 7.396753 | 21.97769 | 5.45E-79 | 3.16E-77 | 169.1557 | UP |
| HK2 | 3.337424 | 5.045531 | 21.53108 | 1.26E-76 | 6.30E-75 | 163.7232 | UP |
| IDO1 | 3.261348 | 4.333246 | 18.29667 | 9.23E-60 | 2.21E-58 | 124.9798 | UP |
| ENO2 | 3.21321 | 6.517157 | 20.1828 | 1.57E-69 | 6.17E-68 | 147.4202 | UP |
| SLC16A3 | 3.12376 | 6.080024 | 23.56921 | 1.90E-87 | 1.61E-85 | 188.5949 | UP |
| TYMP | 3.006188 | 6.059094 | 21.98499 | 4.99E-79 | 3.05E-77 | 169.2446 | UP |
| ABCC3 | 2.391744 | 5.409495 | 15.09632 | 6.94E-44 | 8.48E-43 | 88.54078 | UP |
| LPCAT1 | 2.365348 | 6.497043 | 17.42544 | 2.46E-55 | 4.59E-54 | 114.8174 | UP |
| SLC16A1 | 2.301604 | 5.414122 | 19.4495 | 1.05E-65 | 3.49E-64 | 138.6349 | UP |
| ALDOC | 2.214617 | 5.977167 | 11.90042 | 1.82E-29 | 1.18E-28 | 55.50552 | UP |
| SLC2A5 | 2.140089 | 4.961748 | 10.29923 | 5.13E-23 | 2.17E-22 | 40.76092 | UP |
| TYMS | 2.114964 | 4.908557 | 18.61841 | 2.07E-61 | 5.17E-60 | 128.769 | UP |
| ALOX5 | 2.113641 | 4.63677 | 13.14478 | 7.28E-35 | 6.31E-34 | 67.86225 | UP |
| PFKP | 2.10731 | 8.015577 | 16.26308 | 1.53E-49 | 2.51E-48 | 101.5211 | UP |
| PFKFB4 | 2.095076 | 3.636611 | 17.7633 | 4.82E-57 | 9.81E-56 | 118.7402 | UP |
| PIK3R5 | 2.068421 | 2.744263 | 19.75564 | 2.67E-67 | 9.80E-66 | 142.2944 | UP |
| SLC2A3 | 2.063559 | 5.529597 | 13.05169 | 1.89E-34 | 1.62E-33 | 66.91356 | UP |
| SHMT2 | 2.019709 | 6.835831 | 20.75507 | 1.56E-72 | 6.60E-71 | 154.3192 | UP |
| HSD3B7 | 2.01341 | 6.025284 | 14.98423 | 2.37E-43 | 2.78E-42 | 87.3159 | UP |
| HS3ST2 | 2.004832 | 2.061854 | 11.8385 | 3.31E-29 | 2.10E-28 | 54.90989 | UP |
| PYGL | 1.979912 | 5.275326 | 15.91189 | 8.03E-48 | 1.24E-46 | 97.57214 | UP |
| SLC2A1 | 1.953193 | 7.086745 | 12.95646 | 4.99E-34 | 4.16E-33 | 65.9469 | UP |
| CHST13 | 1.921671 | 3.673528 | 11.69743 | 1.29E-28 | 7.78E-28 | 53.56024 | UP |
| RRM2 | 1.832434 | 2.748449 | 14.32661 | 2.96E-40 | 3.08E-39 | 80.21553 | UP |
| LDHA | 1.798591 | 9.486487 | 17.72817 | 7.26E-57 | 1.45E-55 | 118.3312 | UP |
| ADA | 1.788544 | 3.460672 | 15.68455 | 1.02E-46 | 1.46E-45 | 95.03441 | UP |
| PLCB2 | 1.758248 | 3.150157 | 14.9705 | 2.76E-43 | 3.19E-42 | 87.16613 | UP |
| CES3 | 1.737608 | 4.417755 | 7.54941 | 1.63E-13 | 4.23E-13 | 19.13203 | UP |
| CHST11 | 1.699263 | 4.185473 | 15.96149 | 4.60E-48 | 7.33E-47 | 98.12783 | UP |
| PIK3R6 | 1.667863 | 1.935592 | 15.6698 | 1.21E-46 | 1.70E-45 | 94.87031 | UP |
| HK3 | 1.664495 | 2.309027 | 15.26017 | 1.14E-44 | 1.41E-43 | 90.33867 | UP |
| TBXAS1 | 1.654931 | 3.751412 | 18.10575 | 8.72E-59 | 2.04E-57 | 122.7401 | UP |
| ALOX15B | 1.625239 | 1.68543 | 11.11262 | 3.22E-26 | 1.66E-25 | 48.07577 | UP |
| DPEP2 | 1.607213 | 2.390133 | 18.65252 | 1.38E-61 | 3.53E-60 | 129.1718 | UP |
| INPP5D | 1.565264 | 4.066396 | 15.3747 | 3.22E-45 | 4.07E-44 | 91.60052 | UP |
| SLC6A8 | 1.558693 | 7.432504 | 10.984 | 1.06E-25 | 5.32E-25 | 46.89417 | UP |
| PLD4 | 1.554992 | 2.292539 | 12.81273 | 2.15E-33 | 1.71E-32 | 64.49546 | UP |
| GAPDH | 1.488802 | 11.86857 | 19.12916 | 4.80E-64 | 1.43E-62 | 134.8197 | UP |
| CHSY3 | 1.48766 | 2.189611 | 14.80757 | 1.64E-42 | 1.82E-41 | 85.39376 | UP |
| FOLR2 | 1.478238 | 5.237326 | 9.344503 | 1.79E-19 | 6.32E-19 | 32.6792 | UP |
| ELOVL2 | 1.473845 | 1.758444 | 11.54868 | 5.33E-28 | 3.06E-27 | 52.14816 | UP |
| ABCA1 | 1.463449 | 4.778093 | 13.21171 | 3.66E-35 | 3.27E-34 | 68.54675 | UP |
| PLA1A | 1.395039 | 5.620158 | 10.32636 | 4.04E-23 | 1.72E-22 | 40.99867 | UP |
| SLC27A3 | 1.393316 | 4.200827 | 14.90869 | 5.42E-43 | 6.15E-42 | 86.49268 | UP |
| LCAT | 1.371518 | 3.489078 | 11.11908 | 3.03E-26 | 1.57E-25 | 48.13533 | UP |
| CHIT1 | 1.368749 | 1.450851 | 7.012931 | 6.31E-12 | 1.50E-11 | 15.5407 | UP |
| ACLY | 1.351114 | 7.801192 | 11.26947 | 7.45E-27 | 3.98E-26 | 49.5289 | UP |
| ABCG1 | 1.335778 | 5.013182 | 13.97302 | 1.29E-38 | 1.29E-37 | 76.46207 | UP |
| SLC5A1 | 1.309914 | 3.59119 | 4.669353 | 3.73E-06 | 6.26E-06 | 2.604418 | UP |
| LPIN3 | 1.309817 | 4.551393 | 10.85503 | 3.46E-25 | 1.68E-24 | 45.71863 | UP |
| B4GALNT1 | 1.306033 | 1.658021 | 7.192659 | 1.90E-12 | 4.68E-12 | 16.71911 | UP |
| FDXR | 1.276777 | 4.386687 | 13.9625 | 1.44E-38 | 1.41E-37 | 76.35117 | UP |
| MOGAT3 | 1.23457 | 1.978098 | 7.353831 | 6.35E-13 | 1.60E-12 | 17.79715 | UP |
| HSD17B7 | 1.233784 | 3.186855 | 11.86712 | 2.51E-29 | 1.61E-28 | 55.18496 | UP |
| ADCY7 | 1.219539 | 1.924528 | 10.89958 | 2.30E-25 | 1.14E-24 | 46.12369 | UP |
| DEGS1 | 1.210775 | 7.217834 | 12.19579 | 1.01E-30 | 7.25E-30 | 58.37263 | UP |
| ENPP2 | 1.196677 | 6.908393 | 5.967721 | 4.12E-09 | 8.38E-09 | 9.199748 | UP |
| SMOX | 1.192882 | 4.237332 | 10.70889 | 1.31E-24 | 6.16E-24 | 44.39781 | UP |
| ALDOA | 1.185086 | 9.848792 | 16.93458 | 7.15E-53 | 1.27E-51 | 109.1626 | UP |
| B3GNT4 | 1.18129 | 2.096297 | 9.157634 | 8.28E-19 | 2.84E-18 | 31.16523 | UP |
| GLB1L | 1.161042 | 5.299119 | 9.230786 | 4.56E-19 | 1.57E-18 | 31.75516 | UP |
| ENTPD2 | 1.146828 | 3.575798 | 7.73464 | 4.40E-14 | 1.17E-13 | 20.42292 | UP |
| ALOX5AP | 1.12162 | 4.374337 | 8.615817 | 6.14E-17 | 1.86E-16 | 26.90771 | UP |
| CSAD | 1.117413 | 3.45771 | 8.638682 | 5.14E-17 | 1.57E-16 | 27.08333 | UP |
| SPHK1 | 1.102011 | 2.281649 | 8.82897 | 1.16E-17 | 3.67E-17 | 28.55884 | UP |
| NUDT1 | 1.10067 | 3.682455 | 15.69587 | 9.03E-47 | 1.32E-45 | 95.16047 | UP |
| VKORC1 | 1.096284 | 6.259906 | 12.95381 | 5.13E-34 | 4.24E-33 | 65.91999 | UP |
| FOLH1 | 1.069065 | 4.090571 | 6.846416 | 1.88E-11 | 4.36E-11 | 14.47154 | UP |
| LIPA | 1.061197 | 6.798142 | 8.901453 | 6.50E-18 | 2.12E-17 | 29.12739 | UP |
| TPI1 | 1.040559 | 10.01412 | 13.3308 | 1.07E-35 | 9.98E-35 | 69.76929 | UP |
| HSD17B3 | 1.034603 | 1.197077 | 9.496377 | 5.08E-20 | 1.85E-19 | 33.92642 | UP |
| PLA2G2D | 1.034173 | 1.217833 | 6.771425 | 3.04E-11 | 6.98E-11 | 13.99721 | UP |
| IDUA | 1.025054 | 3.323211 | 11.6083 | 3.02E-28 | 1.76E-27 | 52.71277 | UP |
| PYCR1 | 1.01175 | 2.810895 | 6.825246 | 2.15E-11 | 4.96E-11 | 14.33718 | UP |
| ABCD1 | 1.007191 | 4.274161 | 14.33018 | 2.85E-40 | 2.99E-39 | 80.2536 | UP |
| ALDH3B1 | 0.997048 | 4.515773 | 8.217237 | 1.29E-15 | 3.63E-15 | 23.90532 | NOT |
| SLC37A4 | 0.995277 | 6.254137 | 9.488259 | 5.44E-20 | 1.97E-19 | 33.85938 | NOT |
| NADSYN1 | 0.99129 | 3.64022 | 15.69119 | 9.52E-47 | 1.38E-45 | 95.10839 | NOT |
| BCAT1 | 0.989324 | 2.596572 | 8.166492 | 1.88E-15 | 5.27E-15 | 23.53118 | NOT |
| MAN2B1 | 0.987055 | 5.5516 | 11.51862 | 7.10E-28 | 4.02E-27 | 51.86423 | NOT |
| ABCB4 | 0.975882 | 1.480183 | 8.637812 | 5.18E-17 | 1.58E-16 | 27.07664 | NOT |
| CYP2C8 | 0.975061 | 1.769967 | 6.608749 | 8.56E-11 | 1.91E-10 | 12.98371 | NOT |
| PLA2G4C | 0.975005 | 3.355068 | 10.49579 | 8.94E-24 | 4.00E-23 | 42.49371 | NOT |
| PNPLA7 | 0.974623 | 2.230061 | 8.537645 | 1.13E-16 | 3.36E-16 | 26.31005 | NOT |
| AMY2B | 0.968666 | 1.934984 | 8.057045 | 4.23E-15 | 1.17E-14 | 22.73054 | NOT |
| ENTPD1 | 0.967396 | 4.321267 | 11.16324 | 2.01E-26 | 1.06E-25 | 48.5433 | NOT |
| HSD17B14 | 0.966801 | 6.197018 | 7.152287 | 2.49E-12 | 6.07E-12 | 16.45221 | NOT |
| SLC5A4 | 0.965545 | 1.724194 | 8.64876 | 4.75E-17 | 1.46E-16 | 27.16085 | NOT |
| GDPD5 | 0.96119 | 2.134665 | 11.81257 | 4.25E-29 | 2.66E-28 | 54.66104 | NOT |
| GGT1 | 0.952779 | 6.206163 | 4.956814 | 9.34E-07 | 1.65E-06 | 3.937526 | NOT |
| MTHFD2 | 0.950164 | 4.012201 | 8.992956 | 3.13E-18 | 1.04E-17 | 29.85022 | NOT |
| CYP21A2 | 0.942383 | 1.194415 | 9.3499 | 1.71E-19 | 6.06E-19 | 32.72327 | NOT |
| CHST14 | 0.933896 | 4.955643 | 11.5616 | 4.72E-28 | 2.72E-27 | 52.27038 | NOT |
| ADCY3 | 0.930402 | 4.160772 | 8.827801 | 1.17E-17 | 3.70E-17 | 28.54969 | NOT |
| SLC5A9 | 0.927835 | 3.416528 | 5.833151 | 8.90E-09 | 1.77E-08 | 8.448719 | NOT |
| CYB5R3 | 0.922722 | 7.815713 | 11.54756 | 5.39E-28 | 3.07E-27 | 52.13762 | NOT |
| GUK1 | 0.9202 | 6.915688 | 13.146 | 7.19E-35 | 6.31E-34 | 67.87473 | NOT |
| SLC2A2 | 0.912033 | 4.081993 | 3.7557 | 0.000189696 | 0.000284674 | -1.13546 | NOT |
| GYS1 | 0.911763 | 6.077002 | 11.73521 | 8.96E-29 | 5.44E-28 | 53.92071 | NOT |
| PGK1 | 0.903601 | 8.991235 | 8.311846 | 6.31E-16 | 1.80E-15 | 24.60781 | NOT |
| PIK3CG | 0.901642 | 2.388614 | 7.377587 | 5.39E-13 | 1.36E-12 | 17.95773 | NOT |
| ADPGK | 0.8963 | 4.958364 | 15.95385 | 5.01E-48 | 7.87E-47 | 98.04215 | NOT |
| DECR2 | 0.892386 | 4.836752 | 8.439246 | 2.40E-16 | 6.99E-16 | 25.56383 | NOT |
| ELOVL5 | 0.885342 | 6.086575 | 10.44297 | 1.43E-23 | 6.28E-23 | 42.02583 | NOT |
| TK1 | 0.880033 | 4.83558 | 6.729686 | 3.98E-11 | 8.98E-11 | 13.73515 | NOT |
| LRAT | 0.867542 | 1.250357 | 8.783656 | 1.65E-17 | 5.21E-17 | 28.20521 | NOT |
| PLA2G6 | 0.861447 | 2.633534 | 7.385497 | 5.10E-13 | 1.29E-12 | 18.0113 | NOT |
| SRM | 0.856541 | 5.875924 | 10.85935 | 3.33E-25 | 1.62E-24 | 45.75783 | NOT |
| UPP1 | 0.851761 | 4.398207 | 8.502636 | 1.47E-16 | 4.38E-16 | 26.04378 | NOT |
| ADCY8 | 0.849628 | 1.058429 | 5.005056 | 7.35E-07 | 1.31E-06 | 4.168451 | NOT |
| IMPDH1 | 0.830755 | 4.435421 | 9.291998 | 2.76E-19 | 9.61E-19 | 32.25151 | NOT |
| PGLS | 0.830041 | 5.653517 | 13.73341 | 1.61E-37 | 1.54E-36 | 73.94575 | NOT |
| ACAD11 | 0.819326 | 2.219565 | 6.07811 | 2.16E-09 | 4.52E-09 | 9.827171 | NOT |
| CHST1 | 0.814873 | 3.023285 | 4.933763 | 1.05E-06 | 1.84E-06 | 3.827914 | NOT |
| PIK3CD | 0.813451 | 3.952941 | 8.661157 | 4.32E-17 | 1.33E-16 | 27.2563 | NOT |
| CSGALNACT2 | 0.810313 | 4.643286 | 8.397042 | 3.31E-16 | 9.60E-16 | 25.24585 | NOT |
| PLD2 | 0.808221 | 4.277105 | 10.24472 | 8.30E-23 | 3.46E-22 | 40.28442 | NOT |
| SLC5A10 | 0.796892 | 3.909172 | 3.536685 | 0.000436453 | 0.000642701 | -1.9173 | NOT |
| ALOX12 | 0.787851 | 1.868353 | 10.34811 | 3.33E-23 | 1.42E-22 | 41.18971 | NOT |
| LPGAT1 | 0.783873 | 4.960918 | 8.968837 | 3.80E-18 | 1.25E-17 | 29.65914 | NOT |
| GPX1 | 0.783516 | 8.722602 | 9.54353 | 3.43E-20 | 1.26E-19 | 34.31668 | NOT |
| CPT1B | 0.753985 | 1.271057 | 7.22658 | 1.51E-12 | 3.75E-12 | 16.94433 | NOT |
| NME4 | 0.751347 | 5.884168 | 9.440647 | 8.08E-20 | 2.90E-19 | 33.46702 | NOT |
| CHPF2 | 0.750517 | 5.423524 | 10.77486 | 7.20E-25 | 3.46E-24 | 44.99257 | NOT |
| PLB1 | 0.739741 | 1.423119 | 10.37438 | 2.64E-23 | 1.14E-22 | 41.42074 | NOT |
| SRD5A3 | 0.733011 | 4.172484 | 9.611972 | 1.93E-20 | 7.22E-20 | 34.88566 | NOT |
| FAR2 | 0.732456 | 2.958459 | 7.758404 | 3.71E-14 | 9.94E-14 | 20.59039 | NOT |
| STARD3 | 0.730199 | 4.657761 | 12.69258 | 7.23E-33 | 5.64E-32 | 63.2892 | NOT |
| CHSY1 | 0.728806 | 5.39112 | 6.87614 | 1.55E-11 | 3.61E-11 | 14.66079 | NOT |
| ASNS | 0.723925 | 3.918836 | 8.352986 | 4.62E-16 | 1.33E-15 | 24.91527 | NOT |
| CHPF | 0.715453 | 6.586067 | 6.436655 | 2.50E-10 | 5.44E-10 | 11.93475 | NOT |
| SLC16A8 | 0.713581 | 0.986873 | 8.833391 | 1.12E-17 | 3.57E-17 | 28.59341 | NOT |
| ABCC1 | 0.711043 | 4.930819 | 7.859956 | 1.79E-14 | 4.84E-14 | 21.31078 | NOT |
| BAAT | 0.710582 | 0.892971 | 4.785977 | 2.14E-06 | 3.68E-06 | 3.136375 | NOT |
| NME2 | 0.709176 | 5.710748 | 8.072468 | 3.78E-15 | 1.05E-14 | 22.84284 | NOT |
| PLA2G5 | 0.70184 | 0.789595 | 8.880979 | 7.65E-18 | 2.49E-17 | 28.96643 | NOT |
| AKR1C3 | 0.696572 | 6.08847 | 5.184003 | 2.97E-07 | 5.41E-07 | 5.04295 | NOT |
| PIP4K2A | 0.695989 | 5.088432 | 7.824387 | 2.31E-14 | 6.23E-14 | 21.05759 | NOT |
| ARSA | 0.695227 | 5.373906 | 9.418395 | 9.72E-20 | 3.47E-19 | 33.28416 | NOT |
| NPC1 | 0.691842 | 4.357697 | 8.348029 | 4.80E-16 | 1.38E-15 | 24.87816 | NOT |
| NME1 | 0.683802 | 4.599069 | 8.566253 | 9.02E-17 | 2.70E-16 | 26.52828 | NOT |
| NT5E | 0.683456 | 4.393374 | 4.862533 | 1.48E-06 | 2.56E-06 | 3.492181 | NOT |
| KCNS3 | 0.679257 | 4.143752 | 5.321769 | 1.45E-07 | 2.70E-07 | 5.735322 | NOT |
| ADCY4 | 0.665548 | 3.060208 | 5.461412 | 6.93E-08 | 1.30E-07 | 6.453964 | NOT |
| UGCG | 0.665526 | 6.146213 | 7.570631 | 1.41E-13 | 3.66E-13 | 19.27862 | NOT |
| MAN2C1 | 0.657824 | 4.453914 | 6.825597 | 2.15E-11 | 4.96E-11 | 14.33941 | NOT |
| FADS2 | 0.654677 | 3.634661 | 4.668804 | 3.74E-06 | 6.26E-06 | 2.601941 | NOT |
| CHST2 | 0.654387 | 4.235361 | 6.193387 | 1.09E-09 | 2.33E-09 | 10.49321 | NOT |
| ENO1 | 0.654091 | 10.89031 | 6.207025 | 1.01E-09 | 2.15E-09 | 10.57274 | NOT |
| MGLL | 0.648595 | 6.494645 | 7.342046 | 6.88E-13 | 1.73E-12 | 17.71764 | NOT |
| GUSB | 0.647905 | 6.087888 | 11.28476 | 6.46E-27 | 3.47E-26 | 49.67123 | NOT |
| GALNS | 0.643834 | 3.564299 | 9.803174 | 3.81E-21 | 1.46E-20 | 36.49089 | NOT |
| PGAM1 | 0.641431 | 6.386867 | 6.277242 | 6.61E-10 | 1.42E-09 | 10.98462 | NOT |
| ALDH1L2 | 0.641108 | 1.530201 | 5.965921 | 4.16E-09 | 8.46E-09 | 9.189605 | NOT |
| B4GALT7 | 0.638951 | 4.419835 | 9.548148 | 3.30E-20 | 1.22E-19 | 34.35497 | NOT |
| ST3GAL2 | 0.631374 | 3.868493 | 9.099804 | 1.32E-18 | 4.49E-18 | 30.70138 | NOT |
| CNDP2 | 0.629975 | 7.859223 | 5.492783 | 5.85E-08 | 1.11E-07 | 6.617727 | NOT |
| CACNA1C | 0.620711 | 1.965832 | 5.92892 | 5.15E-09 | 1.04E-08 | 8.981638 | NOT |
| SLC27A1 | 0.62061 | 3.82191 | 9.57311 | 2.67E-20 | 9.94E-20 | 34.56222 | NOT |
| MTHFD1L | 0.616579 | 3.804414 | 6.602183 | 8.93E-11 | 1.99E-10 | 12.94325 | NOT |
| HDC | 0.610834 | 0.851966 | 7.759568 | 3.68E-14 | 9.88E-14 | 20.59861 | NOT |
| APRT | 0.60826 | 6.936415 | 9.553706 | 3.15E-20 | 1.17E-19 | 34.40109 | NOT |
| ALOX12B | 0.605581 | 0.868856 | 8.229798 | 1.17E-15 | 3.31E-15 | 23.99822 | NOT |
| TDO2 | 0.602149 | 1.009271 | 5.356826 | 1.21E-07 | 2.25E-07 | 5.914144 | NOT |
| CYP27A1 | 0.600878 | 6.050971 | 4.466654 | 9.50E-06 | 1.55E-05 | 1.708937 | NOT |
| GPI | 0.590304 | 7.623803 | 8.596849 | 7.12E-17 | 2.15E-16 | 26.76231 | NOT |
| NT5C | 0.58987 | 5.450731 | 8.643191 | 4.97E-17 | 1.52E-16 | 27.118 | NOT |
| GAMT | 0.589576 | 6.226894 | 4.09196 | 4.86E-05 | 7.61E-05 | 0.151891 | NOT |
| SLC10A2 | 0.576588 | 2.977648 | 2.307327 | 0.021376007 | 0.027598131 | -5.45967 | NOT |
| TPK1 | 0.576302 | 2.443431 | 7.255593 | 1.24E-12 | 3.09E-12 | 17.13768 | NOT |
| ACOT7 | 0.565561 | 5.190812 | 4.822669 | 1.80E-06 | 3.09E-06 | 3.306256 | NOT |
| DDHD1 | 0.563589 | 2.048489 | 9.461469 | 6.80E-20 | 2.44E-19 | 33.63843 | NOT |
| SLC5A8 | 0.560954 | 3.557869 | 1.957343 | 0.050770295 | 0.062820387 | -6.20088 | NOT |
| SLC19A1 | 0.559057 | 2.296963 | 7.506383 | 2.21E-13 | 5.70E-13 | 18.83587 | NOT |
| ACBD6 | 0.558731 | 3.784492 | 10.60742 | 3.28E-24 | 1.52E-23 | 43.48795 | NOT |
| CHST9 | 0.557974 | 2.229825 | 3.275644 | 0.001114957 | 0.001607408 | -2.79023 | NOT |
| SLC25A27 | 0.553527 | 2.48913 | 3.927543 | 9.58E-05 | 0.000147155 | -0.49066 | NOT |
| PLD3 | 0.552107 | 7.006672 | 8.691567 | 3.40E-17 | 1.06E-16 | 27.49091 | NOT |
| STS | 0.550671 | 4.172022 | 5.298962 | 1.64E-07 | 3.02E-07 | 5.619555 | NOT |
| ADCY2 | 0.548782 | 0.702786 | 5.484738 | 6.11E-08 | 1.16E-07 | 6.575649 | NOT |
| PLCB3 | 0.545922 | 4.181568 | 7.790763 | 2.94E-14 | 7.92E-14 | 20.81911 | NOT |
| CYP2R1 | 0.535137 | 2.850441 | 8.988565 | 3.24E-18 | 1.07E-17 | 29.8154 | NOT |
| PGS1 | 0.53503 | 4.081159 | 7.757452 | 3.74E-14 | 9.98E-14 | 20.58368 | NOT |
| HEXB | 0.533943 | 6.24133 | 7.132466 | 2.85E-12 | 6.92E-12 | 16.32164 | NOT |
| GPX4 | 0.522025 | 8.505131 | 7.180604 | 2.06E-12 | 5.05E-12 | 16.63928 | NOT |
| ASPG | 0.518743 | 2.31684 | 2.415547 | 0.016009267 | 0.020914719 | -5.20628 | NOT |
| NUDT18 | 0.514486 | 4.177945 | 6.691111 | 5.09E-11 | 1.14E-10 | 13.49418 | NOT |
| ACHE | 0.512148 | 1.895764 | 3.890351 | 0.000111296 | 0.000170035 | -0.63254 | NOT |
| B4GALT5 | 0.511604 | 6.542508 | 5.42415 | 8.45E-08 | 1.59E-07 | 6.260549 | NOT |
| CHST12 | 0.505029 | 2.688299 | 10.48335 | 9.99E-24 | 4.43E-23 | 42.38339 | NOT |
| DGUOK | 0.504277 | 5.982548 | 9.717425 | 7.91E-21 | 2.99E-20 | 35.76814 | NOT |
| TCN2 | 0.502549 | 7.408949 | 3.497805 | 0.000503803 | 0.000737926 | -2.05139 | NOT |
| XYLT2 | 0.499492 | 4.6936 | 7.130093 | 2.89E-12 | 7.00E-12 | 16.30603 | NOT |
| PRODH | 0.499163 | 2.643051 | 3.001006 | 0.002802637 | 0.003922266 | -3.63885 | NOT |
| PLA2G1B | 0.493637 | 1.201753 | 4.911698 | 1.17E-06 | 2.04E-06 | 3.723429 | NOT |
| ACBD4 | 0.490856 | 4.507748 | 5.297238 | 1.65E-07 | 3.05E-07 | 5.610825 | NOT |
| ACSL5 | 0.489727 | 4.668948 | 4.35539 | 1.56E-05 | 2.53E-05 | 1.233206 | NOT |
| CHST7 | 0.488046 | 3.183961 | 4.994343 | 7.75E-07 | 1.38E-06 | 4.116993 | NOT |
| PIK3R3 | 0.485322 | 4.801253 | 4.012134 | 6.78E-05 | 0.000105302 | -0.16319 | NOT |
| UCK2 | 0.480677 | 3.31093 | 5.9494 | 4.57E-09 | 9.27E-09 | 9.096604 | NOT |
| KMO | 0.478425 | 3.328411 | 2.541848 | 0.011277121 | 0.015017958 | -4.89615 | NOT |
| SLC25A14 | 0.47829 | 2.985522 | 9.408043 | 1.06E-19 | 3.77E-19 | 33.19919 | NOT |
| PYGM | 0.47684 | 1.865152 | 4.829065 | 1.74E-06 | 3.00E-06 | 3.33599 | NOT |
| IDS | 0.476674 | 5.769226 | 5.989233 | 3.63E-09 | 7.44E-09 | 9.321215 | NOT |
| STARD3NL | 0.46921 | 5.258701 | 6.011647 | 3.19E-09 | 6.58E-09 | 9.448191 | NOT |
| ITPR3 | 0.469118 | 4.582563 | 3.347315 | 0.000867017 | 0.001254894 | -2.55697 | NOT |
| ITPA | 0.468235 | 5.700665 | 8.603891 | 6.74E-17 | 2.04E-16 | 26.81626 | NOT |
| HSD17B11 | 0.46745 | 6.4205 | 4.670911 | 3.70E-06 | 6.22E-06 | 2.611441 | NOT |
| PLD1 | 0.46662 | 3.308619 | 5.229451 | 2.35E-07 | 4.30E-07 | 5.269528 | NOT |
| COX19 | 0.4609 | 2.780527 | 8.14919 | 2.14E-15 | 5.98E-15 | 23.40403 | NOT |
| NMNAT2 | 0.460737 | 1.119642 | 3.694363 | 0.000240577 | 0.000359558 | -1.35894 | NOT |
| UPB1 | 0.456553 | 3.470118 | 2.42136 | 0.015758025 | 0.020660104 | -5.19234 | NOT |
| DDO | 0.454602 | 3.82215 | 4.110637 | 4.50E-05 | 7.08E-05 | 0.22646 | NOT |
| KHK | 0.447364 | 6.507941 | 2.064356 | 0.039413051 | 0.049661348 | -5.98697 | NOT |
| PHOSPHO1 | 0.445386 | 1.361123 | 5.948788 | 4.59E-09 | 9.28E-09 | 9.093163 | NOT |
| B3GAT3 | 0.444683 | 5.506876 | 6.732711 | 3.90E-11 | 8.83E-11 | 13.75409 | NOT |
| MANBA | 0.439438 | 4.283605 | 6.166196 | 1.29E-09 | 2.71E-09 | 10.33512 | NOT |
| PON2 | 0.436765 | 5.987053 | 4.04151 | 6.00E-05 | 9.34E-05 | -0.04792 | NOT |
| NDST2 | 0.434671 | 1.385717 | 8.447772 | 2.24E-16 | 6.58E-16 | 25.62822 | NOT |
| INPP5E | 0.433344 | 3.814509 | 5.7317 | 1.58E-08 | 3.08E-08 | 7.892638 | NOT |
| PIK3C2B | 0.432764 | 3.64223 | 4.092787 | 4.85E-05 | 7.59E-05 | 0.155186 | NOT |
| AANAT | 0.432561 | 0.493018 | 8.982089 | 3.41E-18 | 1.13E-17 | 29.76407 | NOT |
| CACNB3 | 0.424606 | 2.707636 | 4.967628 | 8.85E-07 | 1.57E-06 | 3.989112 | NOT |
| D2HGDH | 0.422068 | 3.955513 | 3.92838 | 9.55E-05 | 0.000146862 | -0.48745 | NOT |
| ARSB | 0.420583 | 4.654498 | 3.789519 | 0.000166167 | 0.000250389 | -1.01073 | NOT |
| HAS3 | 0.417593 | 1.452576 | 5.086203 | 4.89E-07 | 8.80E-07 | 4.561518 | NOT |
| B3GALT6 | 0.415971 | 4.525606 | 6.008844 | 3.24E-09 | 6.67E-09 | 9.43229 | NOT |
| ADSL | 0.41335 | 4.857303 | 6.928362 | 1.10E-11 | 2.59E-11 | 14.99497 | NOT |
| FADS1 | 0.412258 | 3.499387 | 4.549775 | 6.50E-06 | 1.07E-05 | 2.071665 | NOT |
| KCNB1 | 0.402039 | 1.125401 | 3.97582 | 7.87E-05 | 0.00012188 | -0.30458 | NOT |
| MLYCD | 0.399544 | 2.965792 | 4.523064 | 7.35E-06 | 1.21E-05 | 1.954421 | NOT |
| GSTO1 | 0.399298 | 7.558134 | 6.175195 | 1.22E-09 | 2.58E-09 | 10.38737 | NOT |
| PLD6 | 0.398853 | 2.711333 | 5.003124 | 7.42E-07 | 1.32E-06 | 4.159163 | NOT |
| LPCAT4 | 0.390978 | 3.673761 | 6.002458 | 3.36E-09 | 6.91E-09 | 9.396087 | NOT |
| GALE | 0.390638 | 4.969464 | 4.847765 | 1.59E-06 | 2.75E-06 | 3.423136 | NOT |
| CUBN | 0.389272 | 5.85633 | 1.462642 | 0.144088866 | 0.167368271 | -7.04329 | NOT |
| STARD4 | 0.38401 | 3.593285 | 4.238843 | 2.60E-05 | 4.16E-05 | 0.746973 | NOT |
| CAD | 0.380537 | 3.812554 | 4.739479 | 2.68E-06 | 4.56E-06 | 2.922825 | NOT |
| NMRAL1 | 0.379684 | 4.928215 | 6.480971 | 1.90E-10 | 4.16E-10 | 12.20257 | NOT |
| UCP3 | 0.378449 | 0.952302 | 7.165392 | 2.28E-12 | 5.57E-12 | 16.53871 | NOT |
| LTA4H | 0.374722 | 5.627659 | 5.10054 | 4.55E-07 | 8.22E-07 | 4.63157 | NOT |
| GAA | 0.372675 | 6.616821 | 4.745298 | 2.61E-06 | 4.44E-06 | 2.949441 | NOT |
| NDUFS5 | 0.369463 | 9.234162 | 5.766076 | 1.30E-08 | 2.56E-08 | 8.080081 | NOT |
| STARD5 | 0.361058 | 0.874182 | 9.269046 | 3.33E-19 | 1.15E-18 | 32.06511 | NOT |
| DTYMK | 0.359822 | 4.416767 | 5.418265 | 8.72E-08 | 1.63E-07 | 6.230111 | NOT |
| GBA | 0.359115 | 5.840589 | 4.611665 | 4.88E-06 | 8.12E-06 | 2.345795 | NOT |
| ALDH18A1 | 0.358736 | 6.081116 | 4.569015 | 5.95E-06 | 9.84E-06 | 2.156513 | NOT |
| DHODH | 0.358409 | 2.95756 | 6.215898 | 9.56E-10 | 2.04E-09 | 10.62456 | NOT |
| PAOX | 0.352208 | 3.701831 | 4.982622 | 8.22E-07 | 1.46E-06 | 4.060806 | NOT |
| SGSH | 0.347916 | 4.280254 | 6.024365 | 2.96E-09 | 6.14E-09 | 9.520425 | NOT |
| GLRX | 0.344813 | 5.559844 | 3.682437 | 0.000251856 | 0.000375395 | -1.40199 | NOT |
| SLC45A2 | 0.342298 | 0.520372 | 4.930984 | 1.06E-06 | 1.86E-06 | 3.814731 | NOT |
| GLA | 0.340434 | 5.211754 | 5.629919 | 2.77E-08 | 5.36E-08 | 7.343543 | NOT |
| PIP5K1C | 0.337832 | 4.841855 | 4.906605 | 1.20E-06 | 2.09E-06 | 3.699374 | NOT |
| SLC2A14 | 0.334783 | 0.407003 | 6.043041 | 2.66E-09 | 5.52E-09 | 9.626744 | NOT |
| AMPD2 | 0.334539 | 4.564808 | 3.948244 | 8.81E-05 | 0.00013605 | -0.41114 | NOT |
| FAH | 0.331425 | 4.730378 | 3.738504 | 0.000202831 | 0.00030397 | -1.19846 | NOT |
| PFKL | 0.325494 | 6.846439 | 3.78523 | 0.000168991 | 0.000253948 | -1.02661 | NOT |
| MTRR | 0.323496 | 4.737609 | 3.888833 | 0.000111976 | 0.000170838 | -0.6383 | NOT |
| SMPD4 | 0.321151 | 4.38473 | 4.959124 | 9.23E-07 | 1.63E-06 | 3.948535 | NOT |
| DSEL | 0.319609 | 2.541257 | 2.404097 | 0.016514526 | 0.021523672 | -5.23363 | NOT |
| RPIA | 0.317237 | 4.676843 | 4.878865 | 1.37E-06 | 2.38E-06 | 3.568759 | NOT |
| PGM1 | 0.316619 | 6.461767 | 3.528035 | 0.000450664 | 0.000661857 | -1.94726 | NOT |
| TCN1 | 0.313874 | 0.484326 | 2.56113 | 0.010676545 | 0.014252669 | -4.84744 | NOT |
| ABCD4 | 0.313561 | 4.161743 | 4.488472 | 8.60E-06 | 1.41E-05 | 1.803545 | NOT |
| NDUFA7 | 0.313379 | 2.674947 | 5.878897 | 6.86E-09 | 1.38E-08 | 8.70231 | NOT |
| DERA | 0.313263 | 6.098717 | 3.7371 | 0.00020394 | 0.000305217 | -1.2036 | NOT |
| UCP2 | 0.311474 | 6.182503 | 2.527964 | 0.011728024 | 0.01552446 | -4.931 | NOT |
| SOAT1 | 0.311018 | 4.990301 | 3.571591 | 0.000383258 | 0.000568173 | -1.7957 | NOT |
| ACADL | 0.30821 | 4.551473 | 1.824413 | 0.068586645 | 0.083364982 | -6.45094 | NOT |
| NPC2 | 0.307206 | 7.787269 | 4.948935 | 9.71E-07 | 1.71E-06 | 3.900008 | NOT |
| ASPA | 0.30064 | 4.581481 | 1.712055 | 0.087403177 | 0.105305032 | -6.64874 | NOT |
| AGPAT5 | 0.299114 | 5.011657 | 3.139063 | 0.001778101 | 0.002527017 | -3.22124 | NOT |
| MOCS3 | 0.297264 | 2.854632 | 5.275756 | 1.85E-07 | 3.40E-07 | 5.502231 | NOT |
| SLC25A19 | 0.294704 | 2.803802 | 5.530581 | 4.77E-08 | 9.11E-08 | 6.816166 | NOT |
| INPP4A | 0.28906 | 2.95117 | 4.064112 | 5.46E-05 | 8.54E-05 | 0.041303 | NOT |
| SLC25A1 | 0.287824 | 7.245925 | 3.931283 | 9.43E-05 | 0.000145551 | -0.47632 | NOT |
| ELOVL7 | 0.285141 | 5.324425 | 2.009783 | 0.044901276 | 0.056017272 | -6.09746 | NOT |
| INPPL1 | 0.284636 | 5.714382 | 4.413434 | 1.21E-05 | 1.96E-05 | 1.479983 | NOT |
| PLA2G4D | 0.281431 | 0.270544 | 4.671013 | 3.70E-06 | 6.22E-06 | 2.611902 | NOT |
| CACNA1A | 0.273008 | 0.523283 | 5.982163 | 3.79E-09 | 7.73E-09 | 9.281253 | NOT |
| NDUFAF2 | 0.272356 | 5.210105 | 4.048135 | 5.84E-05 | 9.10E-05 | -0.02182 | NOT |
| SCLY | 0.272105 | 0.858708 | 5.987056 | 3.68E-09 | 7.52E-09 | 9.308908 | NOT |
| HSD17B13 | 0.268431 | 0.875869 | 2.38975 | 0.017167441 | 0.022321732 | -5.26772 | NOT |
| PYCR2 | 0.267341 | 5.428495 | 4.588406 | 5.44E-06 | 9.03E-06 | 2.242368 | NOT |
| KDSR | 0.266887 | 5.059432 | 3.534288 | 0.000440348 | 0.000647571 | -1.92561 | NOT |
| DPEP3 | 0.265243 | 0.321695 | 6.501969 | 1.67E-10 | 3.67E-10 | 12.33003 | NOT |
| HAL | 0.262813 | 0.398912 | 5.099426 | 4.57E-07 | 8.25E-07 | 4.626117 | NOT |
| ACADVL | 0.261706 | 7.987752 | 3.281325 | 0.001093132 | 0.001578012 | -2.77192 | NOT |
| MVD | 0.261413 | 3.841577 | 4.204226 | 3.02E-05 | 4.81E-05 | 0.604942 | NOT |
| CYP46A1 | 0.25706 | 0.811707 | 4.69989 | 3.23E-06 | 5.46E-06 | 2.742526 | NOT |
| INPP4B | 0.252515 | 3.0813 | 2.136977 | 0.033003513 | 0.041969785 | -5.83542 | NOT |
| B4GALT3 | 0.249217 | 4.976452 | 4.250292 | 2.48E-05 | 3.96E-05 | 0.794188 | NOT |
| NDST1 | 0.249031 | 5.655072 | 2.377195 | 0.017757288 | 0.023007086 | -5.29738 | NOT |
| ACMSD | 0.242538 | 5.443228 | 1.138988 | 0.255162517 | 0.288467389 | -7.46343 | NOT |
| LIPN | 0.241818 | 0.357927 | 5.393148 | 9.97E-08 | 1.86E-07 | 6.100544 | NOT |
| HS2ST1 | 0.240514 | 4.599602 | 2.802119 | 0.005240664 | 0.007178991 | -4.20837 | NOT |
| PPCDC | 0.239962 | 3.187266 | 4.453931 | 1.01E-05 | 1.64E-05 | 1.65397 | NOT |
| ADCY5 | 0.236926 | 4.313955 | 1.417378 | 0.15689135 | 0.181855095 | -7.10829 | NOT |
| DCK | 0.235468 | 4.607432 | 2.566206 | 0.01052328 | 0.014065137 | -4.83456 | NOT |
| CYP4F22 | 0.235075 | 0.428893 | 5.655465 | 2.41E-08 | 4.67E-08 | 7.480529 | NOT |
| CDIPT | 0.231042 | 6.698512 | 4.11164 | 4.48E-05 | 7.06E-05 | 0.230473 | NOT |
| CACNA1E | 0.229568 | 0.670971 | 3.379328 | 0.00077375 | 0.001122856 | -2.45121 | NOT |
| PIP4K2B | 0.22955 | 5.622526 | 3.059731 | 0.002314217 | 0.003263639 | -3.46343 | NOT |
| GCLM | 0.225467 | 4.240911 | 2.426246 | 0.015549529 | 0.020435462 | -5.18061 | NOT |
| GLUL | 0.224769 | 7.153124 | 2.163235 | 0.03091718 | 0.039499301 | -5.77935 | NOT |
| MTHFR | 0.222627 | 3.762897 | 2.60758 | 0.009345279 | 0.012597803 | -4.72863 | NOT |
| G6PD | 0.217813 | 4.918512 | 2.985228 | 0.00294907 | 0.004116722 | -3.68541 | NOT |
| PLA2G10 | 0.215485 | 0.391747 | 6.285082 | 6.30E-10 | 1.35E-09 | 11.03086 | NOT |
| AGPS | 0.213767 | 4.884681 | 2.285814 | 0.022612862 | 0.029160784 | -5.50869 | NOT |
| B3GAT2 | 0.212511 | 0.418614 | 6.269032 | 6.95E-10 | 1.49E-09 | 10.93625 | NOT |
| PIK3R2 | 0.209776 | 0.670053 | 5.63982 | 2.62E-08 | 5.08E-08 | 7.396566 | NOT |
| HEXA | 0.208415 | 4.810756 | 4.182313 | 3.32E-05 | 5.27E-05 | 0.515603 | NOT |
| ARG1 | 0.207282 | 0.403151 | 3.998173 | 7.18E-05 | 0.000111379 | -0.21769 | NOT |
| NEU1 | 0.205986 | 6.210521 | 2.830084 | 0.004809317 | 0.00661281 | -4.13059 | NOT |
| HYAL2 | 0.200444 | 5.919045 | 1.913693 | 0.056134811 | 0.068838676 | -6.28491 | NOT |
| NSDHL | 0.197271 | 5.119307 | 3.482907 | 0.000532094 | 0.000777295 | -2.1024 | NOT |
| FLAD1 | 0.197008 | 4.272334 | 3.882273 | 0.000114965 | 0.000175154 | -0.66319 | NOT |
| ACER3 | 0.192873 | 3.763627 | 2.820372 | 0.00495532 | 0.006805059 | -4.15769 | NOT |
| INPP5K | 0.19121 | 4.877395 | 3.183025 | 0.001532834 | 0.00218409 | -3.08445 | NOT |
| PNPLA2 | 0.187665 | 6.171352 | 2.603371 | 0.009459497 | 0.012718452 | -4.73948 | NOT |
| BST1 | 0.183529 | 3.337731 | 1.689968 | 0.091553442 | 0.109824194 | -6.68616 | NOT |
| HSD3B2 | 0.182097 | 0.591579 | 2.953504 | 0.003264881 | 0.004534557 | -3.77832 | NOT |
| TKT | 0.181991 | 5.33394 | 2.423057 | 0.015685306 | 0.020589303 | -5.18827 | NOT |
| ST3GAL3 | 0.181632 | 3.530934 | 2.76029 | 0.005951442 | 0.008112251 | -4.32332 | NOT |
| NDUFA12 | 0.181567 | 6.184255 | 3.545983 | 0.000421646 | 0.000622565 | -1.88502 | NOT |
| CACNB2 | 0.180582 | 1.92439 | 1.93242 | 0.053777927 | 0.066169708 | -6.24909 | NOT |
| OCA2 | 0.177954 | 1.18396 | 1.50888 | 0.131855755 | 0.154463611 | -6.97479 | NOT |
| B4GALT2 | 0.177477 | 4.814799 | 2.533321 | 0.011552194 | 0.015325851 | -4.91758 | NOT |
| SLC25A16 | 0.176607 | 3.635942 | 2.603005 | 0.009469466 | 0.012718452 | -4.74042 | NOT |
| SMPD2 | 0.176052 | 4.024639 | 2.908564 | 0.003765254 | 0.005222925 | -3.90828 | NOT |
| ARSG | 0.171162 | 2.615984 | 2.770725 | 0.005766411 | 0.007879568 | -4.2948 | NOT |
| NDUFA2 | 0.170713 | 7.026828 | 2.359166 | 0.018635463 | 0.024116482 | -5.33972 | NOT |
| ASMT | 0.168348 | 0.209411 | 6.561049 | 1.16E-10 | 2.57E-10 | 12.69056 | NOT |
| CHAT | 0.168341 | 0.15132 | 2.380771 | 0.017587498 | 0.022813972 | -5.28895 | NOT |
| GNPAT | 0.168166 | 5.872169 | 2.957324 | 0.003225278 | 0.004490894 | -3.76718 | NOT |
| XYLT1 | 0.167019 | 2.006271 | 1.710614 | 0.087669209 | 0.105509988 | -6.6512 | NOT |
| CYP2C9 | 0.166521 | 0.860658 | 1.288698 | 0.197999476 | 0.226402728 | -7.28201 | NOT |
| GCLC | 0.165967 | 3.843416 | 1.960919 | 0.050350538 | 0.062371162 | -6.19392 | NOT |
| RRM1 | 0.16422 | 5.140952 | 1.906829 | 0.057019924 | 0.069846232 | -6.29795 | NOT |
| LPCAT3 | 0.163348 | 4.419288 | 1.496543 | 0.135037855 | 0.157687517 | -6.99327 | NOT |
| PI4KB | 0.162635 | 5.54647 | 2.571279 | 0.010372042 | 0.013879861 | -4.82166 | NOT |
| ELOVL1 | 0.158117 | 6.421053 | 2.722106 | 0.006675147 | 0.009042687 | -4.42677 | NOT |
| XDH | 0.153519 | 0.381741 | 1.859282 | 0.063476347 | 0.077324454 | -6.38703 | NOT |
| SLC25A32 | 0.144911 | 4.317429 | 2.130884 | 0.033504484 | 0.042557658 | -5.84833 | NOT |
| EXT2 | 0.143541 | 5.769052 | 2.027285 | 0.043074722 | 0.053966053 | -6.06235 | NOT |
| DUOX1 | 0.143521 | 0.98588 | 1.754367 | 0.079877975 | 0.096768472 | -6.57571 | NOT |
| ACADS | 0.142402 | 5.9793 | 1.630787 | 0.103459886 | 0.12316653 | -6.78405 | NOT |
| HGSNAT | 0.140556 | 5.358917 | 1.989284 | 0.047123339 | 0.058505274 | -6.13821 | NOT |
| NDUFA11 | 0.139756 | 4.532592 | 1.927399 | 0.054401491 | 0.066787545 | -6.25873 | NOT |
| AKR1C4 | 0.138681 | 0.533778 | 1.931771 | 0.053858147 | 0.066194371 | -6.25034 | NOT |
| HK1 | 0.138556 | 6.13849 | 1.537272 | 0.124753754 | 0.146769123 | -6.93168 | NOT |
| SLC35D2 | 0.138355 | 5.46222 | 1.938881 | 0.05298419 | 0.06541258 | -6.23665 | NOT |
| NDUFC1 | 0.136068 | 6.728585 | 2.138707 | 0.032862418 | 0.041838726 | -5.83174 | NOT |
| SLC5A5 | 0.135601 | 0.157562 | 4.933415 | 1.05E-06 | 1.84E-06 | 3.826265 | NOT |
| PISD | 0.135196 | 4.538958 | 1.973899 | 0.048851426 | 0.060582377 | -6.16852 | NOT |
| FPGS | 0.134646 | 4.82993 | 2.390365 | 0.017138968 | 0.022311082 | -5.26626 | NOT |
| PMVK | 0.132955 | 7.005067 | 1.893224 | 0.058808884 | 0.07179775 | -6.32367 | NOT |
| G6PC2 | 0.131508 | 0.212842 | 3.807086 | 0.000155059 | 0.000234938 | -0.94552 | NOT |
| TPH1 | 0.129034 | 0.538039 | 2.896179 | 0.003914942 | 0.005423724 | -3.94376 | NOT |
| SRD5A1 | 0.128568 | 3.648912 | 1.519188 | 0.129241762 | 0.151562834 | -6.95923 | NOT |
| SLC10A1 | 0.12324 | 0.189942 | 2.835758 | 0.004725821 | 0.006506136 | -4.11471 | NOT |
| AKR1D1 | 0.122762 | 0.149088 | 2.420224 | 0.015806844 | 0.020699439 | -5.19507 | NOT |
| CYB5A | 0.122591 | 6.578894 | 1.002077 | 0.31671042 | 0.350837323 | -7.60983 | NOT |
| UROC1 | 0.122427 | 0.154396 | 4.871547 | 1.42E-06 | 2.46E-06 | 3.534419 | NOT |
| SLC25A6 | 0.122304 | 9.653583 | 1.552189 | 0.121144228 | 0.142981385 | -6.90871 | NOT |
| NDUFB4 | 0.121585 | 7.19955 | 2.02656 | 0.043149093 | 0.053997727 | -6.06381 | NOT |
| NDUFB10 | 0.120095 | 7.770281 | 2.071123 | 0.038774136 | 0.048912327 | -5.97307 | NOT |
| IMPDH2 | 0.11962 | 6.599453 | 1.669386 | 0.095562334 | 0.114259312 | -6.7206 | NOT |
| FASN | 0.118619 | 4.16572 | 1.312049 | 0.190005332 | 0.218168961 | -7.25171 | NOT |
| NDUFV2 | 0.117521 | 3.929172 | 2.103134 | 0.035869774 | 0.045352588 | -5.90669 | NOT |
| LTC4S | 0.116078 | 0.207642 | 4.754319 | 2.50E-06 | 4.26E-06 | 2.990767 | NOT |
| SLC25A2 | 0.114584 | 0.168895 | 4.393601 | 1.32E-05 | 2.14E-05 | 1.395315 | NOT |
| ACSBG1 | 0.113741 | 0.28085 | 1.901686 | 0.057690724 | 0.070589317 | -6.3077 | NOT |
| PTEN | 0.113284 | 5.004777 | 1.415898 | 0.157323986 | 0.182164615 | -7.11039 | NOT |
| ME1 | 0.113084 | 3.447282 | 0.897501 | 0.369811675 | 0.405172154 | -7.70908 | NOT |
| PDSS1 | 0.112917 | 3.018347 | 1.645402 | 0.100410479 | 0.119665793 | -6.7602 | NOT |
| LIPI | 0.11221 | 0.182588 | 1.837912 | 0.066569423 | 0.081002617 | -6.42634 | NOT |
| NADK | 0.1116 | 4.561501 | 1.788558 | 0.074190364 | 0.090076601 | -6.51541 | NOT |
| HAS1 | 0.109602 | 0.335152 | 1.588361 | 0.112731364 | 0.133338172 | -6.8521 | NOT |
| UCK1 | 0.109062 | 5.598544 | 1.877591 | 0.060921914 | 0.074295017 | -6.35299 | NOT |
| B4GALT4 | 0.107886 | 3.645086 | 1.408895 | 0.159384036 | 0.184162227 | -7.12025 | NOT |
| HNMT | 0.106039 | 5.867839 | 1.016192 | 0.309947312 | 0.344385902 | -7.5956 | NOT |
| PCYT1A | 0.104732 | 4.863434 | 1.596734 | 0.110851278 | 0.131396989 | -6.83881 | NOT |
| IDH3B | 0.104636 | 6.611932 | 2.017891 | 0.044047118 | 0.055058898 | -6.08123 | NOT |
| AMPD3 | 0.103301 | 3.422886 | 1.036364 | 0.30044972 | 0.335187314 | -7.57492 | NOT |
| MBOAT7 | 0.102391 | 5.216024 | 1.53217 | 0.126007292 | 0.148085493 | -6.93948 | NOT |
| VKORC1L1 | 0.101314 | 4.571691 | 1.269147 | 0.204880512 | 0.233542553 | -7.30696 | NOT |
| AMPD1 | 0.099106 | 0.360577 | 1.551013 | 0.12142574 | 0.143160037 | -6.91053 | NOT |
| DCT | 0.093666 | 0.549332 | 1.559937 | 0.119301972 | 0.140958291 | -6.89669 | NOT |
| PTGES3 | 0.090397 | 7.798285 | 1.291738 | 0.196945261 | 0.22543162 | -7.27809 | NOT |
| SAT1 | 0.089759 | 8.5207 | 1.069345 | 0.285344273 | 0.319632077 | -7.54023 | NOT |
| LDHAL6B | 0.088692 | 0.265359 | 3.463915 | 0.000570311 | 0.000830917 | -2.16711 | NOT |
| PPAT | 0.084927 | 3.132216 | 1.065167 | 0.287228643 | 0.321415572 | -7.54468 | NOT |
| GYS2 | 0.084436 | 0.42834 | 1.720383 | 0.085878227 | 0.103694896 | -6.63451 | NOT |
| UMPS | 0.078525 | 3.612779 | 1.311436 | 0.190211988 | 0.218178505 | -7.25251 | NOT |
| PTGS1 | 0.078265 | 4.635612 | 0.369074 | 0.712202896 | 0.739078477 | -8.04373 | NOT |
| SLC5A6 | 0.075564 | 3.682448 | 1.100639 | 0.2714949 | 0.305988105 | -7.50632 | NOT |
| STAR | 0.075341 | 0.1609 | 2.819459 | 0.004969254 | 0.006815685 | -4.16023 | NOT |
| SMPD3 | 0.07515 | 0.88803 | 1.226403 | 0.220527984 | 0.250341364 | -7.3602 | NOT |
| PRPS1 | 0.074904 | 5.809398 | 0.965543 | 0.334661968 | 0.369978055 | -7.64574 | NOT |
| ACOT9 | 0.071729 | 4.190515 | 1.09808 | 0.272609711 | 0.30661624 | -7.50912 | NOT |
| PAICS | 0.068558 | 5.326307 | 0.981381 | 0.326800378 | 0.361650318 | -7.63034 | NOT |
| NUDT19 | 0.066591 | 4.254403 | 1.30633 | 0.191940655 | 0.219932001 | -7.25918 | NOT |
| DIO3 | 0.063092 | 0.539736 | 0.758395 | 0.448512252 | 0.483215942 | -7.82421 | NOT |
| FDPS | 0.062811 | 5.719187 | 0.879287 | 0.379597475 | 0.414243276 | -7.72525 | NOT |
| SLC6A7 | 0.06102 | 0.077549 | 4.696328 | 3.29E-06 | 5.54E-06 | 2.726373 | NOT |
| CYP19A1 | 0.060779 | 0.133864 | 2.540229 | 0.011328901 | 0.015068672 | -4.90022 | NOT |
| DUT | 0.059563 | 4.924822 | 1.027182 | 0.304748409 | 0.339294787 | -7.58438 | NOT |
| EXT1 | 0.057822 | 4.2183 | 0.767939 | 0.442825519 | 0.478495158 | -7.81693 | NOT |
| SULT2A1 | 0.056485 | 0.0923 | 0.903872 | 0.3664259 | 0.402263962 | -7.70335 | NOT |
| ENTPD6 | 0.054542 | 5.135205 | 0.830261 | 0.406721627 | 0.442088725 | -7.76714 | NOT |
| GYG1 | 0.053584 | 5.198484 | 0.705267 | 0.48091752 | 0.515419746 | -7.86309 | NOT |
| TPO | 0.053541 | 0.067687 | 4.89424 | 1.27E-06 | 2.21E-06 | 3.641068 | NOT |
| ACAD9 | 0.053213 | 5.036147 | 0.900899 | 0.36800312 | 0.403592655 | -7.70603 | NOT |
| COX6C | 0.053086 | 6.386464 | 0.778792 | 0.436409337 | 0.472025831 | -7.80854 | NOT |
| ACER1 | 0.05244 | 0.145063 | 2.465694 | 0.013953401 | 0.018427066 | -5.085 | NOT |
| PTDSS2 | 0.046059 | 4.200611 | 0.890388 | 0.373614361 | 0.408524649 | -7.71544 | NOT |
| LIAS | 0.045569 | 3.919782 | 0.70479 | 0.481214617 | 0.515419746 | -7.86343 | NOT |
| PGM2 | 0.044974 | 4.618188 | 0.490864 | 0.623701761 | 0.657157028 | -7.99134 | NOT |
| THEM5 | 0.044746 | 3.327446 | 0.361096 | 0.71815501 | 0.744552791 | -8.04664 | NOT |
| TYR | 0.044654 | 0.116751 | 1.099301 | 0.272077714 | 0.3063311 | -7.50779 | NOT |
| PNPLA5 | 0.043824 | 0.046198 | 2.182605 | 0.029451783 | 0.03771474 | -5.73755 | NOT |
| SLC7A5 | 0.043422 | 4.793764 | 0.220262 | 0.825741668 | 0.84573169 | -8.08761 | NOT |
| FAR1 | 0.042843 | 4.397607 | 0.573475 | 0.566537956 | 0.60037741 | -7.94736 | NOT |
| PLA2G2E | 0.040369 | 0.038028 | 2.8647 | 0.004320091 | 0.005969975 | -4.03326 | NOT |
| DGAT2L6 | 0.040141 | 0.055037 | 3.928665 | 9.53E-05 | 0.000146862 | -0.48636 | NOT |
| KYNU | 0.035477 | 1.434387 | 0.428076 | 0.668749692 | 0.699262986 | -8.0202 | NOT |
| ABCB11 | 0.031691 | 0.04044 | 1.199129 | 0.230951174 | 0.261633667 | -7.39323 | NOT |
| GAPDHS | 0.030987 | 0.052894 | 1.503384 | 0.133266084 | 0.155783945 | -6.98304 | NOT |
| MMAA | 0.030554 | 3.217654 | 0.413003 | 0.679752009 | 0.709418605 | -8.02654 | NOT |
| GDA | 0.029839 | 3.680454 | 0.135188 | 0.892508872 | 0.905682435 | -8.10274 | NOT |
| NDUFA13 | 0.029571 | 4.975643 | 0.383005 | 0.701851438 | 0.730403578 | -8.03849 | NOT |
| LCT | 0.029413 | 0.037283 | 2.598386 | 0.00959635 | 0.012873152 | -4.75231 | NOT |
| TAT | 0.027359 | 0.178187 | 0.471958 | 0.637127958 | 0.6696936 | -8.00044 | NOT |
| IDI2 | 0.025543 | 0.03923 | 2.745452 | 0.006223809 | 0.008462535 | -4.36369 | NOT |
| CHKA | 0.024685 | 3.582535 | 0.310879 | 0.756000795 | 0.78157977 | -8.06352 | NOT |
| NUDT13 | 0.024317 | 2.506861 | 0.318796 | 0.749991866 | 0.776096945 | -8.06103 | NOT |
| HS3ST4 | 0.023685 | 0.04261 | 0.920872 | 0.357487293 | 0.392843179 | -7.68785 | NOT |
| SLCO1B3 | 0.022882 | 0.024801 | 0.959623 | 0.337631502 | 0.372886197 | -7.65144 | NOT |
| SLC23A2 | 0.022155 | 4.496966 | 0.259233 | 0.795544388 | 0.819380924 | -8.07826 | NOT |
| PGK2 | 0.020568 | 0.034046 | 2.219151 | 0.026849612 | 0.034503006 | -5.6577 | NOT |
| HSD3B1 | 0.020539 | 0.048199 | 1.119263 | 0.26347559 | 0.297559701 | -7.48567 | NOT |
| SLCO1B1 | 0.020182 | 0.042278 | 0.648644 | 0.516816433 | 0.550336956 | -7.90142 | NOT |
| ALOX15 | 0.020156 | 0.131566 | 0.87239 | 0.383344736 | 0.417917948 | -7.73129 | NOT |
| LIPT1 | 0.017128 | 3.222607 | 0.283945 | 0.776550428 | 0.800567452 | -8.07154 | NOT |
| SLC6A11 | 0.016959 | 0.048336 | 0.927946 | 0.353808384 | 0.389189222 | -7.68131 | NOT |
| CSGALNACT1 | 0.015485 | 3.701652 | 0.125891 | 0.899860262 | 0.911370271 | -8.10395 | NOT |
| LALBA | 0.015074 | 0.013266 | 2.195679 | 0.028496915 | 0.036534506 | -5.70914 | NOT |
| STARD6 | 0.014707 | 0.162006 | 0.803456 | 0.422029076 | 0.457371413 | -7.78903 | NOT |
| MTAP | 0.014339 | 2.951966 | 0.207223 | 0.835905815 | 0.854550555 | -8.09039 | NOT |
| NDUFS4 | 0.013456 | 6.850308 | 0.194151 | 0.846123715 | 0.863391545 | -8.09302 | NOT |
| CYP7A1 | 0.008728 | 0.103549 | 0.23652 | 0.813109585 | 0.834347522 | -8.08389 | NOT |
| BCO2 | 0.008721 | 0.8245 | 0.121916 | 0.903006272 | 0.9129659 | -8.10444 | NOT |
| CYP11B1 | 0.008537 | 0.008147 | 1.547764 | 0.122206237 | 0.143925976 | -6.91554 | NOT |
| SI | 0.008141 | 0.010814 | 1.481129 | 0.139097089 | 0.161911955 | -7.01615 | NOT |
| PLA2G4E | 0.008124 | 0.043899 | 0.765881 | 0.44404822 | 0.479327698 | -7.81851 | NOT |
| TPH2 | 0.007437 | 0.019615 | 1.221916 | 0.222218913 | 0.252000829 | -7.36569 | NOT |
| PNLIPRP3 | 0.007404 | 0.084007 | 0.139772 | 0.888887321 | 0.902840308 | -8.10211 | NOT |
| MTHFS | 0.007369 | 3.802302 | 0.081295 | 0.935234149 | 0.941223755 | -8.10857 | NOT |
| PRPS1L1 | 0.006715 | 0.015564 | 1.089603 | 0.276325465 | 0.31047805 | -7.51839 | NOT |
| ENO3 | 0.006176 | 1.909933 | 0.058072 | 0.953710409 | 0.955447587 | -8.11019 | NOT |
| KCNC2 | 0.006064 | 0.006043 | 0.828488 | 0.407723515 | 0.442740243 | -7.76861 | NOT |
| NEU2 | 0.004787 | 0.004213 | 0.892166 | 0.372661644 | 0.407888367 | -7.71385 | NOT |
| GMPS | 0.004709 | 4.214047 | 0.068938 | 0.94506168 | 0.94937703 | -8.1095 | NOT |
| CYP11B2 | 0.00407 | 0.004971 | 0.955616 | 0.339650846 | 0.37436466 | -7.65527 | NOT |
| CYP2C19 | 0.00405 | 0.007242 | 0.765176 | 0.444467502 | 0.479327698 | -7.81905 | NOT |
| NEU3 | 0.004002 | 2.728853 | 0.063144 | 0.949672915 | 0.952384757 | -8.10989 | NOT |
| GNPDA1 | 0.003606 | 5.753671 | 0.049031 | 0.960910671 | 0.961785021 | -8.11068 | NOT |
| GK2 | 0.002856 | 0.00383 | 0.629651 | 0.529162633 | 0.562395069 | -7.91356 | NOT |
| IDO2 | 0.001772 | 0.215319 | 0.063 | 0.949787344 | 0.952384757 | -8.1099 | NOT |
| AMY1A | -0.00077 | 0.003832 | -0.19014 | 0.849267603 | 0.865796444 | -8.09379 | NOT |
| FAAH | -0.00354 | 4.912972 | -0.03092 | 0.97534263 | 0.97534263 | -8.1114 | NOT |
| LIPK | -0.00409 | 0.011233 | -0.66283 | 0.507697056 | 0.541149963 | -7.89212 | NOT |
| PCTP | -0.0059 | 3.853524 | -0.09346 | 0.925567691 | 0.932348406 | -8.10751 | NOT |
| UQCRQ | -0.00636 | 7.589185 | -0.08015 | 0.936144322 | 0.941278569 | -8.10867 | NOT |
| UCP1 | -0.00665 | 0.05732 | -0.33901 | 0.734721667 | 0.761011143 | -8.05438 | NOT |
| OTC | -0.00788 | 0.159689 | -0.15385 | 0.877776403 | 0.892378968 | -8.10004 | NOT |
| PIK3C3 | -0.00884 | 3.377344 | -0.12002 | 0.904506014 | 0.913642439 | -8.10467 | NOT |
| NDUFB7 | -0.00928 | 8.502201 | -0.11013 | 0.912343275 | 0.919869479 | -8.10581 | NOT |
| LIPJ | -0.01154 | 0.115123 | -0.67724 | 0.498516689 | 0.531880076 | -7.88246 | NOT |
| LBR | -0.0119 | 4.976129 | -0.12496 | 0.900599532 | 0.911370271 | -8.10407 | NOT |
| SPTLC3 | -0.01288 | 3.950592 | -0.11289 | 0.910158364 | 0.91850844 | -8.1055 | NOT |
| PNLIPRP1 | -0.0145 | 0.039681 | -1.50697 | 0.132344816 | 0.154871594 | -6.97766 | NOT |
| NDUFB11 | -0.01509 | 7.124886 | -0.25515 | 0.798691147 | 0.821852443 | -8.07931 | NOT |
| AMY2A | -0.0151 | 0.048557 | -0.72434 | 0.469141015 | 0.503468406 | -7.84946 | NOT |
| CTPS2 | -0.01514 | 3.643974 | -0.23137 | 0.817102764 | 0.837663598 | -8.08509 | NOT |
| AKR1A1 | -0.01517 | 7.147579 | -0.18308 | 0.854800921 | 0.869825174 | -8.09511 | NOT |
| PEMT | -0.01539 | 4.815757 | -0.20564 | 0.83714159 | 0.855019265 | -8.09072 | NOT |
| NDST4 | -0.01571 | 0.022955 | -2.12447 | 0.034038812 | 0.043087104 | -5.86188 | NOT |
| PSPH | -0.01753 | 4.2979 | -0.18603 | 0.852482906 | 0.868269626 | -8.09456 | NOT |
| SLC46A1 | -0.01811 | 2.649886 | -0.23759 | 0.812280447 | 0.834274969 | -8.08364 | NOT |
| ADCY9 | -0.02025 | 4.179621 | -0.21061 | 0.83326548 | 0.852643747 | -8.08969 | NOT |
| PFAS | -0.02084 | 3.72324 | -0.28423 | 0.776335486 | 0.800567452 | -8.07146 | NOT |
| ENOPH1 | -0.02236 | 6.0156 | -0.37672 | 0.706515243 | 0.734562162 | -8.04088 | NOT |
| ACOX3 | -0.02411 | 3.804893 | -0.4141 | 0.678946113 | 0.70925045 | -8.02609 | NOT |
| NT5C1B | -0.02574 | 0.367112 | -1.04321 | 0.297271064 | 0.331977838 | -7.56781 | NOT |
| TXNRD1 | -0.02587 | 5.369088 | -0.2842 | 0.776355416 | 0.800567452 | -8.07147 | NOT |
| NUDT5 | -0.0265 | 4.936553 | -0.4115 | 0.680856018 | 0.70989727 | -8.02716 | NOT |
| AASDHPPT | -0.02674 | 4.703229 | -0.37035 | 0.711251642 | 0.738788297 | -8.04326 | NOT |
| GALT | -0.02747 | 3.911141 | -0.43012 | 0.667262596 | 0.698371889 | -8.01932 | NOT |
| PLA2G2F | -0.02846 | 0.028311 | -1.93409 | 0.05357224 | 0.065990442 | -6.24589 | NOT |
| PLCB1 | -0.03254 | 3.514192 | -0.2455 | 0.806149547 | 0.82875187 | -8.08172 | NOT |
| MOGAT1 | -0.0347 | 0.456239 | -0.54307 | 0.587285863 | 0.620571037 | -7.96434 | NOT |
| BBOX1 | -0.03492 | 6.831836 | -0.12681 | 0.899137053 | 0.911370271 | -8.10384 | NOT |
| COX4I1 | -0.0384 | 7.471641 | -0.58401 | 0.559436238 | 0.593423204 | -7.94127 | NOT |
| PNLIP | -0.04199 | 0.066722 | -1.03441 | 0.301359129 | 0.335861238 | -7.57693 | NOT |
| LIPF | -0.04245 | 0.090449 | -1.52621 | 0.127484067 | 0.149661125 | -6.94857 | NOT |
| ACOXL | -0.04552 | 0.220428 | -1.62435 | 0.104825276 | 0.124657085 | -6.79448 | NOT |
| DSE | -0.04604 | 2.980029 | -0.43944 | 0.660499099 | 0.691951437 | -8.01527 | NOT |
| PI4K2B | -0.04737 | 3.723144 | -0.69345 | 0.488293574 | 0.521478575 | -7.87135 | NOT |
| GALC | -0.04818 | 4.683434 | -0.4801 | 0.631333785 | 0.664561879 | -7.99657 | NOT |
| MAN2B2 | -0.04883 | 4.574447 | -0.69723 | 0.485929581 | 0.51945825 | -7.86873 | NOT |
| AMD1 | -0.05118 | 5.644062 | -0.45397 | 0.650016845 | 0.68161919 | -8.00878 | NOT |
| AGK | -0.05302 | 3.976859 | -1.00717 | 0.314258912 | 0.348824221 | -7.60472 | NOT |
| ST3GAL1 | -0.0541 | 4.841849 | -0.56121 | 0.574862127 | 0.608027249 | -7.95432 | NOT |
| SLC2A4 | -0.05486 | 2.016412 | -0.4948 | 0.620925001 | 0.654858582 | -7.9894 | NOT |
| CEPT1 | -0.05529 | 3.588121 | -0.70195 | 0.482982739 | 0.516810323 | -7.86542 | NOT |
| ELOVL4 | -0.05696 | 1.341714 | -0.62428 | 0.532684168 | 0.565591298 | -7.91693 | NOT |
| DCTD | -0.05843 | 6.1013 | -1.02249 | 0.306962023 | 0.341413777 | -7.58919 | NOT |
| ATIC | -0.05946 | 5.551871 | -0.78782 | 0.431115637 | 0.466759056 | -7.80147 | NOT |
| SLC26A1 | -0.06179 | 2.860737 | -0.56448 | 0.572636954 | 0.606256641 | -7.95248 | NOT |
| CHST5 | -0.06374 | 0.13415 | -3.91182 | 0.000102073 | 0.000156598 | -0.55079 | NOT |
| ACER2 | -0.0645 | 2.692826 | -0.51765 | 0.604892147 | 0.638561767 | -7.97782 | NOT |
| ACSF3 | -0.0663 | 2.786784 | -1.05836 | 0.290315321 | 0.324539485 | -7.5519 | NOT |
| MMADHC | -0.06701 | 6.724815 | -0.82776 | 0.408135753 | 0.442750817 | -7.76922 | NOT |
| ALDH1A1 | -0.06889 | 8.831793 | -0.45875 | 0.646577346 | 0.678659428 | -8.00659 | NOT |
| COQ5 | -0.0699 | 5.436783 | -1.23389 | 0.217727289 | 0.247417374 | -7.35101 | NOT |
| ARSJ | -0.07406 | 3.581812 | -0.74262 | 0.458 | 0.492473119 | -7.83605 | NOT |
| DGAT1 | -0.07414 | 4.562023 | -1.33077 | 0.183771087 | 0.211452088 | -7.22702 | NOT |
| PANK4 | -0.07475 | 3.612464 | -1.28104 | 0.200673007 | 0.228983722 | -7.29182 | NOT |
| GALK1 | -0.07605 | 4.487057 | -0.88022 | 0.379091457 | 0.41410189 | -7.72443 | NOT |
| DPYD | -0.07726 | 4.155214 | -0.72683 | 0.467615331 | 0.502321157 | -7.84765 | NOT |
| B3GAT1 | -0.07727 | 0.645016 | -1.07239 | 0.283975822 | 0.318423449 | -7.53697 | NOT |
| PLA2G2A | -0.07727 | 0.735353 | -0.47154 | 0.637426545 | 0.6696936 | -8.00064 | NOT |
| LIPM | -0.08084 | 0.575266 | -1.07898 | 0.281029324 | 0.315441078 | -7.52989 | NOT |
| ABCC8 | -0.08204 | 0.182366 | -2.5376 | 0.011413572 | 0.015162958 | -4.90684 | NOT |
| AMACR | -0.0852 | 4.084303 | -0.6345 | 0.525995905 | 0.559570112 | -7.9105 | NOT |
| BCAT2 | -0.08545 | 4.638426 | -1.48751 | 0.137405345 | 0.160281951 | -7.00671 | NOT |
| HPSE | -0.08946 | 2.604423 | -0.85968 | 0.390310565 | 0.425090714 | -7.7423 | NOT |
| AGPAT1 | -0.09046 | 6.19829 | -1.28134 | 0.200570124 | 0.228983722 | -7.29145 | NOT |
| NDUFV3 | -0.09047 | 4.354519 | -1.66752 | 0.095933164 | 0.114578155 | -6.7237 | NOT |
| PCYT1B | -0.09111 | 0.409187 | -1.61739 | 0.10631961 | 0.126161349 | -6.80573 | NOT |
| MGAM | -0.09143 | 3.625024 | -0.40058 | 0.688868502 | 0.717571357 | -8.0316 | NOT |
| GNPDA2 | -0.09255 | 3.265859 | -1.40824 | 0.159578314 | 0.184193227 | -7.12117 | NOT |
| SLC35B2 | -0.09632 | 6.357261 | -1.32829 | 0.184586309 | 0.212168171 | -7.23031 | NOT |
| SOAT2 | -0.1017 | 0.533219 | -1.70342 | 0.089006588 | 0.106885641 | -6.66342 | NOT |
| GART | -0.10791 | 4.200489 | -1.48305 | 0.138586433 | 0.161488429 | -7.01332 | NOT |
| COX7C | -0.10851 | 8.613232 | -1.67546 | 0.094364631 | 0.112950047 | -6.71047 | NOT |
| IDH3G | -0.10924 | 6.019123 | -2.12791 | 0.033751046 | 0.042772063 | -5.85461 | NOT |
| ENTPD7 | -0.10984 | 3.298103 | -1.10278 | 0.270563749 | 0.305251409 | -7.50396 | NOT |
| FIG4 | -0.11296 | 4.399292 | -1.7683 | 0.077518779 | 0.094013954 | -6.55128 | NOT |
| TALDO1 | -0.11355 | 6.762139 | -1.90053 | 0.05784279 | 0.070696744 | -6.30989 | NOT |
| MMAB | -0.11814 | 4.188259 | -2.05105 | 0.040696005 | 0.051160692 | -6.01419 | NOT |
| CDS2 | -0.11822 | 4.909101 | -1.62083 | 0.10557857 | 0.125417307 | -6.80017 | NOT |
| HADHA | -0.11839 | 8.030161 | -1.7208 | 0.085803142 | 0.103694896 | -6.6338 | NOT |
| SLC35B3 | -0.11959 | 5.147437 | -1.71531 | 0.086804232 | 0.104698086 | -6.64318 | NOT |
| CBS | -0.12088 | 0.109123 | -5.02614 | 6.61E-07 | 1.18E-06 | 4.270037 | NOT |
| NDUFAB1 | -0.12388 | 6.440486 | -2.00965 | 0.044915667 | 0.056017272 | -6.09774 | NOT |
| TK2 | -0.12794 | 4.395895 | -2.1546 | 0.031590713 | 0.040312975 | -5.79787 | NOT |
| NDUFS7 | -0.12838 | 4.155533 | -2.03602 | 0.042186769 | 0.052974253 | -6.0447 | NOT |
| AKR1B1 | -0.12916 | 7.360873 | -0.83582 | 0.403588444 | 0.439117001 | -7.76251 | NOT |
| CHIA | -0.13027 | 0.090543 | -4.56014 | 6.20E-06 | 1.02E-05 | 2.117353 | NOT |
| PNPLA6 | -0.13244 | 4.756899 | -2.02865 | 0.042934549 | 0.053851772 | -6.05959 | NOT |
| SLC26A2 | -0.13378 | 3.526994 | -1.44054 | 0.150235471 | 0.174323859 | -7.07528 | NOT |
| GCK | -0.13683 | 0.297129 | -2.44602 | 0.014730213 | 0.019428339 | -5.13286 | NOT |
| AK2 | -0.13702 | 6.216629 | -2.43775 | 0.015068031 | 0.0198501 | -5.15287 | NOT |
| CDA | -0.13764 | 4.819522 | -0.94839 | 0.343314788 | 0.378024291 | -7.66215 | NOT |
| CACNA1D | -0.13829 | 1.787876 | -1.39241 | 0.164315215 | 0.189263598 | -7.14329 | NOT |
| DDHD2 | -0.13943 | 4.018129 | -1.70473 | 0.088762047 | 0.106708471 | -6.6612 | NOT |
| PIK3R1 | -0.14023 | 4.88155 | -1.25757 | 0.209038217 | 0.238035237 | -7.32157 | NOT |
| LIPC | -0.14215 | 3.384105 | -0.75028 | 0.453382129 | 0.487984679 | -7.83034 | NOT |
| ARSH | -0.1454 | 0.039165 | -11.9465 | 1.16E-29 | 7.70E-29 | 55.94958 | NOT |
| COX6A1 | -0.14914 | 7.764152 | -2.05769 | 0.040051438 | 0.050407989 | -6.00063 | NOT |
| PIK3CA | -0.14956 | 3.590066 | -1.59581 | 0.11105865 | 0.131501093 | -6.84029 | NOT |
| COX6B1 | -0.15058 | 8.75335 | -2.46567 | 0.013954315 | 0.018427066 | -5.08505 | NOT |
| LDHAL6A | -0.15116 | 0.361233 | -4.34876 | 1.61E-05 | 2.59E-05 | 1.205202 | NOT |
| G6PC3 | -0.15342 | 5.422017 | -2.64023 | 0.008500439 | 0.011472985 | -4.64386 | NOT |
| ABCG5 | -0.15344 | 0.405962 | -3.65024 | 0.000284835 | 0.000423976 | -1.51752 | NOT |
| PTDSS1 | -0.15553 | 5.07749 | -2.41616 | 0.015982715 | 0.020904859 | -5.20482 | NOT |
| GGPS1 | -0.15918 | 4.723561 | -3.02197 | 0.002618338 | 0.003677574 | -3.57659 | NOT |
| NDUFA3 | -0.15979 | 5.570406 | -2.12923 | 0.03364131 | 0.042682169 | -5.85182 | NOT |
| TH | -0.16211 | 0.066159 | -7.28099 | 1.04E-12 | 2.61E-12 | 17.30743 | NOT |
| NDUFA8 | -0.16338 | 7.293612 | -2.75098 | 0.006121133 | 0.008333226 | -4.34868 | NOT |
| KCNG2 | -0.16478 | 0.168724 | -7.67336 | 6.81E-14 | 1.79E-13 | 19.99303 | NOT |
| DHFR | -0.1663 | 3.107587 | -1.93717 | 0.053193221 | 0.065597022 | -6.23995 | NOT |
| MTR | -0.16654 | 4.763593 | -2.00383 | 0.045537583 | 0.056728586 | -6.10935 | NOT |
| ACOT4 | -0.17003 | 3.533643 | -1.41429 | 0.15779619 | 0.182519252 | -7.11266 | NOT |
| MVK | -0.17375 | 4.034277 | -1.75273 | 0.080159577 | 0.097002789 | -6.57858 | NOT |
| NDUFB8 | -0.1747 | 5.780586 | -3.20365 | 0.001428857 | 0.00204654 | -3.01962 | NOT |
| SDHA | -0.1788 | 6.275238 | -2.15062 | 0.031904587 | 0.040666334 | -5.80636 | NOT |
| NDUFA1 | -0.18327 | 8.494726 | -3.21677 | 0.001366169 | 0.001959304 | -2.97818 | NOT |
| PIP5K1A | -0.1851 | 4.871193 | -2.73968 | 0.006332762 | 0.008600047 | -4.37933 | NOT |
| BCKDHA | -0.19239 | 2.966743 | -2.24176 | 0.025341235 | 0.032602759 | -5.60764 | NOT |
| AMDHD1 | -0.19791 | 2.446722 | -1.39532 | 0.163435528 | 0.188447674 | -7.13924 | NOT |
| RRM2B | -0.2019 | 5.458132 | -2.09526 | 0.036566372 | 0.046180263 | -5.92311 | NOT |
| SLC25A21 | -0.20389 | 0.623322 | -3.54464 | 0.000423754 | 0.000624838 | -1.88969 | NOT |
| COQ7 | -0.20519 | 4.354101 | -3.59728 | 0.000348064 | 0.000516694 | -1.7055 | NOT |
| ADO | -0.20756 | 4.585418 | -2.99172 | 0.002887976 | 0.004036561 | -3.66628 | NOT |
| NUDT15 | -0.20985 | 4.990975 | -3.20292 | 0.001432424 | 0.002048981 | -3.02192 | NOT |
| NDUFS6 | -0.21029 | 6.341566 | -3.02165 | 0.002621107 | 0.003677574 | -3.57756 | NOT |
| UGP2 | -0.2109 | 5.782785 | -3.2268 | 0.001319973 | 0.001897507 | -2.9464 | NOT |
| ENPP7 | -0.21196 | 1.485794 | -1.24885 | 0.212206368 | 0.241392973 | -7.33247 | NOT |
| ASL | -0.21553 | 5.130469 | -2.68447 | 0.007465082 | 0.010100358 | -4.52736 | NOT |
| UQCRB | -0.2165 | 6.012268 | -4.05723 | 5.62E-05 | 8.77E-05 | 0.014069 | NOT |
| ASAH2 | -0.22391 | 0.439714 | -7.67605 | 6.68E-14 | 1.76E-13 | 20.01181 | NOT |
| CERK | -0.22406 | 5.206427 | -2.31753 | 0.020810245 | 0.026899259 | -5.43627 | NOT |
| COX5B | -0.22459 | 7.970962 | -3.0723 | 0.002220399 | 0.003135352 | -3.42546 | NOT |
| DPYS | -0.2248 | 4.995795 | -0.9588 | 0.338043181 | 0.372966399 | -7.65222 | NOT |
| ETFB | -0.22576 | 5.105102 | -3.25512 | 0.001197217 | 0.001723742 | -2.85613 | NOT |
| NAGLU | -0.22579 | 5.630615 | -3.13409 | 0.001808026 | 0.00256623 | -3.2366 | NOT |
| RPE | -0.22831 | 4.942546 | -3.36459 | 0.000815455 | 0.001181819 | -2.50001 | NOT |
| ARSK | -0.22844 | 3.371532 | -2.7662 | 0.005845932 | 0.007978319 | -4.30717 | NOT |
| PIK3CB | -0.22913 | 4.797106 | -2.38129 | 0.017562873 | 0.022808925 | -5.28772 | NOT |
| GDPD1 | -0.22962 | 1.99684 | -3.18839 | 0.001505125 | 0.00214739 | -3.06762 | NOT |
| CRAT | -0.23056 | 5.910579 | -2.84403 | 0.004606443 | 0.006349733 | -4.09151 | NOT |
| RFK | -0.23146 | 5.065575 | -2.53296 | 0.011564051 | 0.015325851 | -4.91849 | NOT |
| PANK2 | -0.23336 | 3.692629 | -3.6228 | 0.00031611 | 0.000469894 | -1.61525 | NOT |
| HSD17B1 | -0.23427 | 0.946455 | -6.32134 | 5.06E-10 | 1.09E-09 | 11.24535 | NOT |
| SLC25A17 | -0.23502 | 4.239136 | -4.35093 | 1.59E-05 | 2.57E-05 | 1.214353 | NOT |
| CYP1A2 | -0.23515 | 0.169568 | -3.3153 | 0.00097064 | 0.001403028 | -2.66176 | NOT |
| MOCS1 | -0.23695 | 4.559784 | -2.27245 | 0.02341226 | 0.030156307 | -5.53891 | NOT |
| PPCS | -0.23786 | 5.949867 | -3.97553 | 7.88E-05 | 0.00012188 | -0.3057 | NOT |
| SYNJ1 | -0.24278 | 3.257231 | -3.01634 | 0.00266677 | 0.003736875 | -3.59337 | NOT |
| AGPAT2 | -0.24337 | 6.286719 | -2.88564 | 0.004046604 | 0.005599075 | -3.97384 | NOT |
| PKLR | -0.24443 | 3.741657 | -1.00362 | 0.315967259 | 0.35036692 | -7.60829 | NOT |
| SGMS2 | -0.24587 | 4.300826 | -2.20917 | 0.027540077 | 0.035348991 | -5.67965 | NOT |
| HMGCLL1 | -0.24705 | 0.516254 | -4.10576 | 4.59E-05 | 7.22E-05 | 0.206946 | NOT |
| HSD17B2 | -0.24862 | 0.855172 | -2.77478 | 0.00569587 | 0.007792857 | -4.28368 | NOT |
| B3GNT2 | -0.2497 | 5.846685 | -3.03676 | 0.002495127 | 0.00350977 | -3.53244 | NOT |
| CYP11A1 | -0.25032 | 0.587681 | -2.17661 | 0.029899139 | 0.038243085 | -5.75054 | NOT |
| SYNJ2 | -0.25075 | 2.787666 | -2.72509 | 0.006615764 | 0.008973293 | -4.41873 | NOT |
| FBP2 | -0.25218 | 0.189886 | -7.68658 | 6.20E-14 | 1.64E-13 | 20.08554 | NOT |
| PON1 | -0.25245 | 0.186897 | -4.73602 | 2.72E-06 | 4.61E-06 | 2.907026 | NOT |
| GBE1 | -0.25419 | 5.411763 | -2.55618 | 0.01082791 | 0.014437214 | -4.85998 | NOT |
| NT5M | -0.25891 | 2.761684 | -2.60353 | 0.009455089 | 0.012718452 | -4.73907 | NOT |
| HYAL3 | -0.26131 | 1.907554 | -4.16098 | 3.63E-05 | 5.76E-05 | 0.429064 | NOT |
| AHCY | -0.26158 | 6.967868 | -3.48746 | 0.000523297 | 0.000765461 | -2.08684 | NOT |
| LIPE | -0.26283 | 2.071372 | -2.85808 | 0.004410018 | 0.0060866 | -4.05197 | NOT |
| TMLHE | -0.26666 | 3.427189 | -3.6932 | 0.000241658 | 0.000360684 | -1.36316 | NOT |
| COX7A2L | -0.26978 | 5.777486 | -4.13957 | 3.98E-05 | 6.30E-05 | 0.342624 | NOT |
| GMPR2 | -0.27031 | 5.959596 | -3.78858 | 0.000166784 | 0.000250974 | -1.01422 | NOT |
| AGPAT3 | -0.27053 | 5.324295 | -3.18157 | 0.001540447 | 0.002192097 | -3.08902 | NOT |
| CRLS1 | -0.27095 | 4.977242 | -4.13101 | 4.12E-05 | 6.52E-05 | 0.308172 | NOT |
| DUOX2 | -0.27937 | 0.255328 | -4.85863 | 1.51E-06 | 2.61E-06 | 3.473911 | NOT |
| B3GALNT1 | -0.27982 | 4.6954 | -2.5867 | 0.009924025 | 0.013296501 | -4.78228 | NOT |
| COQ10B | -0.28324 | 5.307446 | -4.42288 | 1.16E-05 | 1.88E-05 | 1.520451 | NOT |
| COASY | -0.28503 | 5.635401 | -5.12361 | 4.05E-07 | 7.32E-07 | 4.744645 | NOT |
| SLC25A13 | -0.28687 | 5.11616 | -3.51853 | 0.000466773 | 0.000684601 | -1.98008 | NOT |
| B3GNT7 | -0.2876 | 1.830475 | -3.19692 | 0.001462048 | 0.00208864 | -3.04082 | NOT |
| AGXT2 | -0.29136 | 4.841773 | -1.15835 | 0.247183433 | 0.279734338 | -7.44122 | NOT |
| COQ6 | -0.2924 | 2.711106 | -5.5229 | 4.97E-08 | 9.48E-08 | 6.775736 | NOT |
| MBOAT1 | -0.29258 | 3.081515 | -2.95461 | 0.003253326 | 0.00452422 | -3.77508 | NOT |
| NDUFB6 | -0.29959 | 6.326629 | -4.65338 | 4.02E-06 | 6.72E-06 | 2.532494 | NOT |
| INPP5F | -0.2996 | 3.598508 | -3.79706 | 0.000161309 | 0.000243737 | -0.98277 | NOT |
| ACOT8 | -0.30302 | 3.719371 | -5.9065 | 5.85E-09 | 1.18E-08 | 8.856208 | NOT |
| GPT | -0.30341 | 3.779869 | -1.66194 | 0.097046294 | 0.115781912 | -6.73295 | NOT |
| ENTPD4 | -0.30854 | 3.901157 | -4.21877 | 2.84E-05 | 4.52E-05 | 0.664475 | NOT |
| LSS | -0.30929 | 3.873308 | -4.47817 | 9.01E-06 | 1.48E-05 | 1.758805 | NOT |
| MTHFD1 | -0.30955 | 4.88353 | -3.84067 | 0.000135744 | 0.000206086 | -0.82004 | NOT |
| PLA2G12A | -0.31129 | 4.819515 | -4.18165 | 3.33E-05 | 5.28E-05 | 0.512903 | NOT |
| B4GALT1 | -0.31258 | 6.907682 | -3.2265 | 0.001321355 | 0.001897507 | -2.94736 | NOT |
| NPC1L1 | -0.31654 | 0.729744 | -2.40683 | 0.016392561 | 0.021390056 | -5.2271 | NOT |
| MCAT | -0.31754 | 4.523117 | -5.75396 | 1.39E-08 | 2.73E-08 | 8.01388 | NOT |
| CYP4F11 | -0.31756 | 2.274622 | -1.95033 | 0.051601674 | 0.06377735 | -6.21451 | NOT |
| CS | -0.31757 | 6.242647 | -4.70986 | 3.08E-06 | 5.22E-06 | 2.7878 | NOT |
| OLAH | -0.32149 | 0.342138 | -7.9048 | 1.29E-14 | 3.52E-14 | 21.6313 | NOT |
| SGPL1 | -0.32152 | 5.225815 | -4.34762 | 1.62E-05 | 2.60E-05 | 1.200402 | NOT |
| GPD2 | -0.32681 | 4.160152 | -3.7945 | 0.000162941 | 0.000245864 | -0.99225 | NOT |
| COQ2 | -0.33217 | 3.080598 | -4.39046 | 1.34E-05 | 2.17E-05 | 1.381951 | NOT |
| AKR1B10 | -0.33706 | 1.255245 | -1.47975 | 0.139464385 | 0.162167889 | -7.01819 | NOT |
| SLC3A2 | -0.33754 | 6.566725 | -4.50761 | 7.88E-06 | 1.29E-05 | 1.886882 | NOT |
| HS3ST5 | -0.34295 | 0.120314 | -7.90934 | 1.25E-14 | 3.42E-14 | 21.66383 | NOT |
| COX18 | -0.34304 | 3.183779 | -5.84968 | 8.10E-09 | 1.62E-08 | 8.540167 | NOT |
| PLA2G3 | -0.34336 | 0.097675 | -10.1509 | 1.89E-22 | 7.56E-22 | 39.4683 | NOT |
| GLUD2 | -0.35014 | 2.682853 | -3.84051 | 0.00013583 | 0.000206086 | -0.82064 | NOT |
| MDH2 | -0.35037 | 7.149015 | -5.18295 | 2.99E-07 | 5.43E-07 | 5.037707 | NOT |
| GLS2 | -0.35109 | 0.475914 | -8.84476 | 1.02E-17 | 3.27E-17 | 28.6824 | NOT |
| PIK3R4 | -0.35217 | 4.417417 | -4.22136 | 2.80E-05 | 4.48E-05 | 0.675097 | NOT |
| HLCS | -0.3528 | 3.491617 | -4.63856 | 4.31E-06 | 7.19E-06 | 2.465994 | NOT |
| PTGES2 | -0.35502 | 5.119839 | -5.21799 | 2.49E-07 | 4.56E-07 | 5.212211 | NOT |
| CHST3 | -0.36089 | 4.14423 | -2.97848 | 0.003013845 | 0.004201812 | -3.70527 | NOT |
| ARSF | -0.36159 | 1.888709 | -1.99329 | 0.046682331 | 0.058023237 | -6.13029 | NOT |
| COX11 | -0.3618 | 4.594784 | -5.44378 | 7.61E-08 | 1.43E-07 | 6.362289 | NOT |
| ADCY6 | -0.36311 | 4.485996 | -4.10542 | 4.59E-05 | 7.22E-05 | 0.205611 | NOT |
| SLC44A5 | -0.36499 | 1.200922 | -2.00232 | 0.045699492 | 0.056865884 | -6.11234 | NOT |
| NAALAD2 | -0.37685 | 0.689421 | -7.72456 | 4.73E-14 | 1.25E-13 | 20.35201 | NOT |
| ACAA2 | -0.38428 | 7.032786 | -3.12464 | 0.001866158 | 0.002645327 | -3.26573 | NOT |
| NDUFAF1 | -0.38531 | 4.89043 | -7.22573 | 1.52E-12 | 3.77E-12 | 16.9387 | NOT |
| SLC27A5 | -0.38566 | 1.349803 | -6.9361 | 1.05E-11 | 2.47E-11 | 15.04464 | NOT |
| ABCC5 | -0.38812 | 3.328986 | -4.34538 | 1.63E-05 | 2.62E-05 | 1.190969 | NOT |
| CYP2U1 | -0.38856 | 3.209208 | -4.57664 | 5.74E-06 | 9.52E-06 | 2.190239 | NOT |
| PI4KA | -0.3893 | 4.357925 | -4.73663 | 2.71E-06 | 4.61E-06 | 2.90981 | NOT |
| GDPD3 | -0.39471 | 2.645642 | -3.08881 | 0.002102463 | 0.002972634 | -3.37534 | NOT |
| NDUFA9 | -0.39554 | 3.754302 | -7.28258 | 1.03E-12 | 2.58E-12 | 17.3181 | NOT |
| SLC25A11 | -0.39679 | 6.496621 | -6.74991 | 3.49E-11 | 7.96E-11 | 13.86193 | NOT |
| PFKFB1 | -0.40078 | 0.494409 | -9.8816 | 1.95E-21 | 7.50E-21 | 37.15597 | NOT |
| AADAT | -0.40239 | 2.4547 | -5.77084 | 1.26E-08 | 2.49E-08 | 8.10613 | NOT |
| GNMT | -0.40334 | 0.972586 | -5.51006 | 5.33E-08 | 1.01E-07 | 6.708273 | NOT |
| GBA2 | -0.40381 | 5.688837 | -5.0402 | 6.16E-07 | 1.11E-06 | 4.33798 | NOT |
| CYC1 | -0.40413 | 7.44082 | -5.30627 | 1.58E-07 | 2.91E-07 | 5.656608 | NOT |
| SPTLC1 | -0.40477 | 5.552287 | -4.33932 | 1.68E-05 | 2.69E-05 | 1.165432 | NOT |
| STARD10 | -0.40486 | 4.653612 | -4.99562 | 7.70E-07 | 1.37E-06 | 4.123127 | NOT |
| BPGM | -0.41554 | 5.33257 | -5.66855 | 2.24E-08 | 4.35E-08 | 7.550927 | NOT |
| NDUFS3 | -0.41625 | 5.695954 | -8.17369 | 1.78E-15 | 5.01E-15 | 23.58411 | NOT |
| GLUD1 | -0.41822 | 7.830978 | -5.37861 | 1.08E-07 | 2.01E-07 | 6.025781 | NOT |
| XYLB | -0.4202 | 2.346471 | -3.86468 | 0.000123357 | 0.000187681 | -0.72973 | NOT |
| NDUFB5 | -0.42178 | 5.385746 | -6.09885 | 1.91E-09 | 4.01E-09 | 9.946204 | NOT |
| DBH | -0.42255 | 0.970985 | -5.8407 | 8.52E-09 | 1.70E-08 | 8.49046 | NOT |
| DIO2 | -0.4257 | 1.4072 | -3.05905 | 0.002319364 | 0.00326671 | -3.46547 | NOT |
| EBP | -0.42587 | 5.309561 | -6.74568 | 3.59E-11 | 8.14E-11 | 13.83541 | NOT |
| NDUFB1 | -0.43025 | 5.626132 | -6.52613 | 1.44E-10 | 3.17E-10 | 12.47713 | NOT |
| PDHX | -0.4311 | 4.47985 | -6.53828 | 1.33E-10 | 2.95E-10 | 12.55126 | NOT |
| ARSI | -0.43588 | 0.744545 | -3.89872 | 0.000107613 | 0.000164637 | -0.60074 | NOT |
| ACSBG2 | -0.43687 | 0.385037 | -11.6534 | 1.96E-28 | 1.17E-27 | 53.14073 | NOT |
| GLCE | -0.43751 | 4.156627 | -4.67378 | 3.65E-06 | 6.16E-06 | 2.624404 | NOT |
| NFS1 | -0.44053 | 4.211435 | -8.44172 | 2.35E-16 | 6.88E-16 | 25.58248 | NOT |
| BHMT | -0.44318 | 7.150589 | -1.68754 | 0.092018639 | 0.110261986 | -6.69024 | NOT |
| NDUFS8 | -0.44719 | 5.18778 | -5.82951 | 9.08E-09 | 1.81E-08 | 8.428632 | NOT |
| COX16 | -0.44839 | 4.858885 | -7.55118 | 1.61E-13 | 4.19E-13 | 19.14423 | NOT |
| GLO1 | -0.44886 | 7.067773 | -5.94685 | 4.64E-09 | 9.37E-09 | 9.082261 | NOT |
| NDUFB2 | -0.45125 | 5.657398 | -6.89027 | 1.41E-11 | 3.30E-11 | 14.75099 | NOT |
| IDH1 | -0.45368 | 5.36227 | -4.86899 | 1.44E-06 | 2.49E-06 | 3.522451 | NOT |
| COQ9 | -0.45435 | 5.831315 | -6.48029 | 1.91E-10 | 4.17E-10 | 12.19843 | NOT |
| ACAT2 | -0.46183 | 3.42684 | -7.6214 | 9.84E-14 | 2.58E-13 | 19.63071 | NOT |
| GCSH | -0.46401 | 3.347414 | -6.11399 | 1.75E-09 | 3.68E-09 | 10.03331 | NOT |
| SLC9A1 | -0.46494 | 4.363222 | -6.16702 | 1.28E-09 | 2.71E-09 | 10.33992 | NOT |
| PYGB | -0.46535 | 6.407651 | -5.56313 | 4.00E-08 | 7.66E-08 | 6.988019 | NOT |
| PANK3 | -0.46645 | 4.351568 | -5.16223 | 3.32E-07 | 6.02E-07 | 4.935026 | NOT |
| ETFA | -0.46692 | 5.700465 | -6.71606 | 4.34E-11 | 9.78E-11 | 13.64989 | NOT |
| SLC44A1 | -0.47087 | 4.600994 | -5.46477 | 6.80E-08 | 1.28E-07 | 6.471458 | NOT |
| NDUFA10 | -0.47101 | 4.982578 | -7.9558 | 8.90E-15 | 2.44E-14 | 21.99762 | NOT |
| GNS | -0.47472 | 7.555684 | -5.20102 | 2.72E-07 | 4.97E-07 | 5.127576 | NOT |
| UQCRC2 | -0.47612 | 6.7646 | -6.00934 | 3.23E-09 | 6.66E-09 | 9.435111 | NOT |
| NQO1 | -0.47948 | 5.278865 | -4.90048 | 1.23E-06 | 2.15E-06 | 3.670481 | NOT |
| PTGR2 | -0.47962 | 2.975708 | -5.9537 | 4.46E-09 | 9.06E-09 | 9.120795 | NOT |
| ELOVL6 | -0.48231 | 2.877355 | -5.03759 | 6.25E-07 | 1.12E-06 | 4.325361 | NOT |
| GDE1 | -0.4869 | 5.321335 | -6.62877 | 7.55E-11 | 1.69E-10 | 13.10729 | NOT |
| PCBD1 | -0.49034 | 6.969696 | -6.48387 | 1.87E-10 | 4.09E-10 | 12.22016 | NOT |
| PI4K2A | -0.49476 | 4.42929 | -7.73072 | 4.53E-14 | 1.20E-13 | 20.39533 | NOT |
| NDUFA6 | -0.49548 | 6.95568 | -7.9017 | 1.32E-14 | 3.60E-14 | 21.60909 | NOT |
| MCEE | -0.49841 | 3.984246 | -8.38734 | 3.56E-16 | 1.03E-15 | 25.17297 | NOT |
| NUDT16 | -0.50067 | 4.811187 | -6.18816 | 1.13E-09 | 2.39E-09 | 10.46276 | NOT |
| B3GNT3 | -0.50188 | 3.297618 | -2.42834 | 0.015460802 | 0.02034316 | -5.17556 | NOT |
| SMS | -0.50432 | 7.097912 | -7.58738 | 1.25E-13 | 3.26E-13 | 19.39454 | NOT |
| ABCG8 | -0.50484 | 0.249849 | -8.77158 | 1.82E-17 | 5.68E-17 | 28.11121 | NOT |
| BHMT2 | -0.50566 | 7.406601 | -3.1 | 0.002025905 | 0.002868077 | -3.34126 | NOT |
| GGT5 | -0.50736 | 4.252549 | -3.46901 | 0.00055982 | 0.000816714 | -2.14979 | NOT |
| PECR | -0.50992 | 4.929844 | -4.89473 | 1.27E-06 | 2.21E-06 | 3.64337 | NOT |
| ADCY1 | -0.51078 | 1.539444 | -4.93635 | 1.03E-06 | 1.82E-06 | 3.840198 | NOT |
| GRHPR | -0.5121 | 5.389333 | -6.5927 | 9.47E-11 | 2.11E-10 | 12.88485 | NOT |
| SLC25A12 | -0.51774 | 3.963343 | -8.28618 | 7.66E-16 | 2.18E-15 | 24.41661 | NOT |
| HSD17B10 | -0.52049 | 7.150486 | -9.11137 | 1.20E-18 | 4.12E-18 | 30.79395 | NOT |
| SCP2 | -0.52445 | 5.971149 | -7.36797 | 5.76E-13 | 1.45E-12 | 17.89266 | NOT |
| HS3ST1 | -0.52501 | 2.571445 | -4.77949 | 2.21E-06 | 3.79E-06 | 3.106475 | NOT |
| ABHD3 | -0.5251 | 3.215146 | -6.0277 | 2.90E-09 | 6.03E-09 | 9.539364 | NOT |
| HAGH | -0.52991 | 4.630291 | -8.10648 | 2.94E-15 | 8.18E-15 | 23.09109 | NOT |
| ACACA | -0.53024 | 3.15055 | -6.74577 | 3.59E-11 | 8.14E-11 | 13.83596 | NOT |
| OGDH | -0.5359 | 7.23965 | -6.43712 | 2.49E-10 | 5.43E-10 | 11.93754 | NOT |
| PIK3C2A | -0.53694 | 4.925841 | -5.4834 | 6.15E-08 | 1.16E-07 | 6.568637 | NOT |
| INMT | -0.53723 | 3.613675 | -3.54967 | 0.000415898 | 0.000614903 | -1.87218 | NOT |
| ETNK1 | -0.53855 | 4.670388 | -7.01677 | 6.15E-12 | 1.47E-11 | 15.56564 | NOT |
| GSTZ1 | -0.5395 | 2.494674 | -7.61473 | 1.03E-13 | 2.70E-13 | 19.58433 | NOT |
| NDUFA5 | -0.53978 | 5.033202 | -8.26624 | 8.90E-16 | 2.53E-15 | 24.26839 | NOT |
| HSD17B8 | -0.53985 | 5.584796 | -5.80998 | 1.01E-08 | 2.01E-08 | 8.320916 | NOT |
| ALDH7A1 | -0.54017 | 5.483674 | -7.00717 | 6.56E-12 | 1.55E-11 | 15.50335 | NOT |
| AK5 | -0.54118 | 0.551157 | -7.49536 | 2.38E-13 | 6.11E-13 | 18.76024 | NOT |
| NMNAT3 | -0.54314 | 3.052074 | -6.52612 | 1.44E-10 | 3.17E-10 | 12.47706 | NOT |
| AACS | -0.54625 | 2.604075 | -8.24878 | 1.02E-15 | 2.88E-15 | 24.13884 | NOT |
| CHPT1 | -0.54778 | 4.697323 | -6.79012 | 2.70E-11 | 6.20E-11 | 14.11507 | NOT |
| EHHADH | -0.554 | 6.247428 | -3.41671 | 0.000676699 | 0.000984615 | -2.32651 | NOT |
| AGPAT4 | -0.55407 | 2.398954 | -6.9137 | 1.21E-11 | 2.85E-11 | 14.90089 | NOT |
| STARD7 | -0.56064 | 6.719086 | -8.66095 | 4.32E-17 | 1.33E-16 | 27.25469 | NOT |
| THTPA | -0.56169 | 3.213082 | -6.34464 | 4.39E-10 | 9.49E-10 | 11.38379 | NOT |
| PNPLA3 | -0.5629 | 1.030047 | -6.07777 | 2.17E-09 | 4.52E-09 | 9.825247 | NOT |
| GPAM | -0.56464 | 2.349647 | -8.02719 | 5.27E-15 | 1.45E-14 | 22.51362 | NOT |
| NDUFB9 | -0.56535 | 6.875148 | -8.67578 | 3.85E-17 | 1.19E-16 | 27.36902 | NOT |
| AK1 | -0.56871 | 2.71014 | -7.41959 | 4.03E-13 | 1.02E-12 | 18.24269 | NOT |
| IDH3A | -0.57057 | 3.409887 | -6.01295 | 3.17E-09 | 6.55E-09 | 9.4556 | NOT |
| ELOVL3 | -0.5745 | 0.537182 | -11.4262 | 1.70E-27 | 9.52E-27 | 50.99442 | NOT |
| ACAD10 | -0.57654 | 3.857328 | -7.49296 | 2.42E-13 | 6.20E-13 | 18.74379 | NOT |
| HMGCS1 | -0.57912 | 4.817607 | -6.29209 | 6.04E-10 | 1.30E-09 | 11.07221 | NOT |
| CYP7B1 | -0.58252 | 3.607426 | -4.5484 | 6.54E-06 | 1.08E-05 | 2.06563 | NOT |
| ACO2 | -0.58276 | 6.91382 | -5.4227 | 8.52E-08 | 1.60E-07 | 6.253066 | NOT |
| MOCS2 | -0.58585 | 4.929883 | -10.03 | 5.42E-22 | 2.12E-21 | 38.4248 | NOT |
| LDHC | -0.58706 | 0.601035 | -7.06194 | 4.56E-12 | 1.09E-11 | 15.85954 | NOT |
| HAS2 | -0.58971 | 0.969283 | -5.68657 | 2.03E-08 | 3.94E-08 | 7.648095 | NOT |
| GSR | -0.5927 | 6.458725 | -7.97065 | 7.99E-15 | 2.20E-14 | 22.10469 | NOT |
| MOCOS | -0.59364 | 1.821766 | -3.90382 | 0.000105423 | 0.000161511 | -0.58131 | NOT |
| THEM4 | -0.59682 | 3.842028 | -10.2357 | 8.98E-23 | 3.73E-22 | 40.20544 | NOT |
| COX8A | -0.59836 | 9.3324 | -8.75963 | 2.00E-17 | 6.22E-17 | 28.01829 | NOT |
| ENTPD5 | -0.59963 | 4.158108 | -4.62238 | 4.65E-06 | 7.73E-06 | 2.393626 | NOT |
| GLYCTK | -0.60195 | 3.644676 | -6.51203 | 1.57E-10 | 3.46E-10 | 12.3912 | NOT |
| PFKFB3 | -0.6035 | 6.892197 | -4.10512 | 4.60E-05 | 7.22E-05 | 0.204387 | NOT |
| NDUFC2 | -0.60478 | 5.14671 | -10.1375 | 2.12E-22 | 8.47E-22 | 39.3523 | NOT |
| SLC27A2 | -0.60836 | 4.981141 | -2.64352 | 0.008419274 | 0.011377397 | -4.63527 | NOT |
| AKR1C2 | -0.61037 | 1.846713 | -4.3828 | 1.38E-05 | 2.24E-05 | 1.349364 | NOT |
| PNPLA8 | -0.6111 | 4.575637 | -7.18561 | 1.99E-12 | 4.89E-12 | 16.6724 | NOT |
| HS3ST3A1 | -0.61235 | 0.716226 | -5.3186 | 1.48E-07 | 2.74E-07 | 5.7192 | NOT |
| NDUFV1 | -0.61339 | 6.603624 | -9.94045 | 1.17E-21 | 4.57E-21 | 37.65749 | NOT |
| MMACHC | -0.61661 | 2.637896 | -10.1828 | 1.43E-22 | 5.76E-22 | 39.74485 | NOT |
| SQLE | -0.61834 | 3.836348 | -6.83895 | 1.97E-11 | 4.57E-11 | 14.4241 | NOT |
| ODC1 | -0.62147 | 6.747777 | -6.16486 | 1.30E-09 | 2.72E-09 | 10.32735 | NOT |
| NDUFB3 | -0.62272 | 6.475233 | -10.4332 | 1.56E-23 | 6.82E-23 | 41.93916 | NOT |
| NMNAT1 | -0.62483 | 3.152956 | -9.03344 | 2.26E-18 | 7.60E-18 | 30.17185 | NOT |
| COX7B | -0.62512 | 6.608347 | -8.62703 | 5.63E-17 | 1.71E-16 | 26.99379 | NOT |
| ACBD7 | -0.62893 | 0.479474 | -14.8166 | 1.48E-42 | 1.66E-41 | 85.49214 | NOT |
| DGAT2 | -0.62895 | 0.928483 | -7.50261 | 2.27E-13 | 5.84E-13 | 18.80998 | NOT |
| HGD | -0.62937 | 5.796714 | -4.63235 | 4.44E-06 | 7.39E-06 | 2.438176 | NOT |
| SPTLC2 | -0.63479 | 5.219793 | -7.0128 | 6.32E-12 | 1.50E-11 | 15.53985 | NOT |
| DHTKD1 | -0.63596 | 5.146339 | -5.80013 | 1.07E-08 | 2.12E-08 | 8.266782 | NOT |
| ABHD4 | -0.63797 | 5.123371 | -8.96236 | 4.00E-18 | 1.31E-17 | 29.60792 | NOT |
| SLC25A20 | -0.63886 | 4.955226 | -10.0907 | 3.20E-22 | 1.27E-21 | 38.94773 | NOT |
| NUDT9 | -0.64301 | 4.938789 | -10.5107 | 7.82E-24 | 3.53E-23 | 42.62603 | NOT |
| SLC44A2 | -0.65492 | 7.018909 | -6.76931 | 3.09E-11 | 7.06E-11 | 13.98388 | NOT |
| HADHB | -0.65646 | 7.172413 | -8.50116 | 1.49E-16 | 4.42E-16 | 26.03258 | NOT |
| NDUFS2 | -0.65684 | 6.497807 | -11.3603 | 3.18E-27 | 1.75E-26 | 50.37621 | NOT |
| ACOT6 | -0.65728 | 0.479768 | -12.0862 | 2.98E-30 | 2.03E-29 | 57.30363 | NOT |
| CPT2 | -0.65781 | 5.007293 | -6.85617 | 1.76E-11 | 4.10E-11 | 14.53355 | NOT |
| SLC44A3 | -0.6589 | 5.42045 | -5.60637 | 3.15E-08 | 6.08E-08 | 7.217782 | NOT |
| CPS1 | -0.65993 | 1.109788 | -5.60488 | 3.18E-08 | 6.11E-08 | 7.209791 | NOT |
| SLC19A3 | -0.66249 | 1.848353 | -5.56038 | 4.06E-08 | 7.76E-08 | 6.973465 | NOT |
| ETFDH | -0.66397 | 4.68973 | -7.89348 | 1.40E-14 | 3.81E-14 | 21.55028 | NOT |
| COQ10A | -0.67051 | 3.04203 | -10.3172 | 4.38E-23 | 1.86E-22 | 40.91809 | NOT |
| DECR1 | -0.67073 | 6.107276 | -11.9262 | 1.41E-29 | 9.26E-29 | 55.75429 | NOT |
| SLCO1A2 | -0.67629 | 0.25499 | -10.0476 | 4.65E-22 | 1.84E-21 | 38.57598 | NOT |
| SDHC | -0.67667 | 4.724808 | -8.8793 | 7.75E-18 | 2.52E-17 | 28.95322 | NOT |
| CHST6 | -0.67681 | 0.250965 | -12.4532 | 7.94E-32 | 5.94E-31 | 60.90558 | NOT |
| ALDH9A1 | -0.68144 | 6.878837 | -8.13863 | 2.31E-15 | 6.46E-15 | 23.32651 | NOT |
| MTHFD2L | -0.68402 | 1.77216 | -12.3227 | 2.90E-31 | 2.10E-30 | 59.61738 | NOT |
| NUDT7 | -0.68466 | 3.13955 | -9.75919 | 5.55E-21 | 2.12E-20 | 36.11956 | NOT |
| HAO1 | -0.68655 | 0.187563 | -10.8631 | 3.22E-25 | 1.57E-24 | 45.79227 | NOT |
| HPRT1 | -0.6867 | 5.720885 | -8.7733 | 1.79E-17 | 5.62E-17 | 28.12461 | NOT |
| PAPSS1 | -0.68865 | 5.446685 | -8.85623 | 9.31E-18 | 3.01E-17 | 28.77222 | NOT |
| CYP4B1 | -0.68881 | 0.828928 | -8.51002 | 1.39E-16 | 4.15E-16 | 26.09988 | NOT |
| ALDH3A2 | -0.68954 | 6.147656 | -5.82879 | 9.12E-09 | 1.81E-08 | 8.424608 | NOT |
| COX5A | -0.68974 | 7.208032 | -9.54566 | 3.37E-20 | 1.24E-19 | 34.33433 | NOT |
| NUDT12 | -0.69072 | 4.447635 | -6.80441 | 2.46E-11 | 5.67E-11 | 14.20527 | NOT |
| HMGCL | -0.69279 | 5.12285 | -10.2651 | 6.93E-23 | 2.90E-22 | 40.46239 | NOT |
| HSD17B4 | -0.69694 | 5.556513 | -9.01456 | 2.63E-18 | 8.77E-18 | 30.02173 | NOT |
| DLAT | -0.69789 | 5.106494 | -8.33774 | 5.19E-16 | 1.49E-15 | 24.80118 | NOT |
| PAPSS2 | -0.70403 | 5.067257 | -5.69963 | 1.88E-08 | 3.67E-08 | 7.718679 | NOT |
| ASAH1 | -0.70694 | 7.445263 | -6.3729 | 3.70E-10 | 8.01E-10 | 11.55228 | NOT |
| NDST3 | -0.70721 | 0.221637 | -22.5852 | 3.26E-82 | 2.24E-80 | 176.5636 | NOT |
| ACSS3 | -0.71289 | 3.374012 | -5.16874 | 3.21E-07 | 5.83E-07 | 4.967271 | NOT |
| AHCYL1 | -0.7184 | 6.793756 | -10.5882 | 3.90E-24 | 1.80E-23 | 43.31613 | NOT |
| DHCR24 | -0.72049 | 6.303653 | -5.81712 | 9.74E-09 | 1.93E-08 | 8.360265 | NOT |
| CPT1A | -0.72253 | 5.83388 | -7.17182 | 2.19E-12 | 5.35E-12 | 16.58121 | NOT |
| QDPR | -0.72748 | 5.591403 | -9.10592 | 1.26E-18 | 4.29E-18 | 30.75029 | NOT |
| B4GALT6 | -0.73192 | 2.228095 | -7.43275 | 3.68E-13 | 9.38E-13 | 18.33232 | NOT |
| ACACB | -0.73625 | 3.397539 | -7.85535 | 1.85E-14 | 4.99E-14 | 21.27791 | NOT |
| PNPLA4 | -0.73822 | 4.535465 | -9.29002 | 2.81E-19 | 9.74E-19 | 32.23539 | NOT |
| SDHB | -0.73971 | 6.510402 | -12.5042 | 4.78E-32 | 3.62E-31 | 61.41093 | NOT |
| SLC25A10 | -0.74355 | 3.796619 | -7.10637 | 3.39E-12 | 8.16E-12 | 16.15018 | NOT |
| SLC45A3 | -0.74383 | 2.836838 | -6.75182 | 3.45E-11 | 7.88E-11 | 13.87396 | NOT |
| DHCR7 | -0.74662 | 4.306932 | -9.02707 | 2.38E-18 | 7.98E-18 | 30.12116 | NOT |
| LPIN1 | -0.75496 | 2.766859 | -9.90394 | 1.61E-21 | 6.23E-21 | 37.3461 | NOT |
| ACBD5 | -0.76145 | 5.309674 | -8.77637 | 1.75E-17 | 5.50E-17 | 28.14852 | NOT |
| GCDH | -0.76368 | 3.827363 | -13.3231 | 1.16E-35 | 1.07E-34 | 69.69032 | NOT |
| NEU4 | -0.76576 | 0.498144 | -9.09874 | 1.33E-18 | 4.52E-18 | 30.69289 | NOT |
| SGMS1 | -0.77027 | 4.324376 | -9.18906 | 6.41E-19 | 2.20E-18 | 31.41821 | NOT |
| NT5C2 | -0.77097 | 3.802742 | -8.48155 | 1.73E-16 | 5.12E-16 | 25.88378 | NOT |
| SARDH | -0.78008 | 2.828555 | -6.64125 | 6.98E-11 | 1.57E-10 | 13.18451 | NOT |
| UQCRH | -0.7806 | 7.16752 | -8.43696 | 2.44E-16 | 7.09E-16 | 25.5466 | NOT |
| MDH1 | -0.78152 | 6.400703 | -11.7417 | 8.41E-29 | 5.14E-28 | 53.9829 | NOT |
| PDSS2 | -0.78292 | 4.329051 | -9.9548 | 1.04E-21 | 4.05E-21 | 37.7801 | NOT |
| AASS | -0.78347 | 2.924956 | -7.47593 | 2.73E-13 | 6.97E-13 | 18.62708 | NOT |
| PGD | -0.78623 | 6.037076 | -10.5034 | 8.35E-24 | 3.75E-23 | 42.56116 | NOT |
| PNPO | -0.79795 | 5.238344 | -8.85422 | 9.46E-18 | 3.05E-17 | 28.75652 | NOT |
| ADAL | -0.80402 | 2.587698 | -12.4399 | 9.06E-32 | 6.74E-31 | 60.77417 | NOT |
| PTGIS | -0.80751 | 2.902548 | -4.34935 | 1.60E-05 | 2.59E-05 | 1.207697 | NOT |
| ADI1 | -0.80921 | 6.087923 | -10.2015 | 1.21E-22 | 4.94E-22 | 39.90801 | NOT |
| ACOX1 | -0.81132 | 4.857773 | -11.75 | 7.77E-29 | 4.83E-28 | 54.06219 | NOT |
| MCCC2 | -0.81569 | 5.51345 | -11.0835 | 4.22E-26 | 2.15E-25 | 47.80729 | NOT |
| BTD | -0.8191 | 4.335338 | -11.4687 | 1.14E-27 | 6.40E-27 | 51.39347 | NOT |
| ITPR1 | -0.82132 | 4.074596 | -7.2173 | 1.61E-12 | 3.98E-12 | 16.88264 | NOT |
| SGPP2 | -0.82145 | 5.408085 | -5.24621 | 2.16E-07 | 3.95E-07 | 5.353536 | NOT |
| HS3ST6 | -0.82689 | 0.17395 | -18.027 | 2.20E-58 | 4.84E-57 | 121.8183 | NOT |
| IVD | -0.82932 | 5.462563 | -11.3318 | 4.15E-27 | 2.27E-26 | 50.10976 | NOT |
| COQ3 | -0.83404 | 3.56701 | -12.4034 | 1.30E-31 | 9.62E-31 | 60.41369 | NOT |
| HACL1 | -0.83606 | 3.922739 | -12.9789 | 3.97E-34 | 3.34E-33 | 66.17394 | NOT |
| CBR4 | -0.8365 | 4.132858 | -12.1294 | 1.95E-30 | 1.36E-29 | 57.72436 | NOT |
| FAAH2 | -0.83735 | 3.678135 | -8.37501 | 3.91E-16 | 1.13E-15 | 25.08033 | NOT |
| CH25H | -0.84311 | 1.94281 | -6.90592 | 1.27E-11 | 2.99E-11 | 14.8511 | NOT |
| PON3 | -0.84511 | 0.777013 | -9.38785 | 1.25E-19 | 4.44E-19 | 33.03364 | NOT |
| DMGDH | -0.8469 | 5.374737 | -5.26951 | 1.91E-07 | 3.51E-07 | 5.470727 | NOT |
| GLTP | -0.85098 | 6.220228 | -11.6752 | 1.59E-28 | 9.58E-28 | 53.34852 | NOT |
| FH | -0.85306 | 6.445411 | -13.2585 | 2.26E-35 | 2.04E-34 | 69.02678 | NOT |
| SMPD1 | -0.85633 | 5.733176 | -11.5846 | 3.79E-28 | 2.19E-27 | 52.48815 | NOT |
| BDH2 | -0.85644 | 6.01557 | -10.2856 | 5.79E-23 | 2.43E-22 | 40.64127 | NOT |
| HSD17B12 | -0.85702 | 4.91862 | -10.6073 | 3.28E-24 | 1.52E-23 | 43.48687 | NOT |
| ABHD5 | -0.86116 | 3.484406 | -11.7457 | 8.09E-29 | 4.97E-28 | 54.02113 | NOT |
| NAGS | -0.86718 | 2.554033 | -7.12823 | 2.93E-12 | 7.07E-12 | 16.29377 | NOT |
| CRYM | -0.86874 | 3.397613 | -5.50166 | 5.58E-08 | 1.06E-07 | 6.664244 | NOT |
| ACSL1 | -0.88405 | 6.903148 | -7.08791 | 3.84E-12 | 9.21E-12 | 16.02922 | NOT |
| SLC23A1 | -0.88869 | 3.246288 | -4.73646 | 2.72E-06 | 4.61E-06 | 2.909012 | NOT |
| SLC6A12 | -0.89159 | 4.790008 | -5.58365 | 3.57E-08 | 6.86E-08 | 7.096831 | NOT |
| ACOT1 | -0.89349 | 3.002194 | -7.49937 | 2.32E-13 | 5.96E-13 | 18.78769 | NOT |
| SHMT1 | -0.89523 | 6.016157 | -7.21705 | 1.61E-12 | 3.98E-12 | 16.88099 | NOT |
| RDH11 | -0.89545 | 5.711895 | -10.6469 | 2.30E-24 | 1.08E-23 | 43.84096 | NOT |
| ACSL3 | -0.89632 | 4.729789 | -10.7571 | 8.46E-25 | 4.03E-24 | 44.83217 | NOT |
| GPD1 | -0.89829 | 4.710415 | -3.8031 | 0.000157518 | 0.000238336 | -0.96035 | NOT |
| MAT1A | -0.90076 | 1.087044 | -5.35327 | 1.23E-07 | 2.29E-07 | 5.895976 | NOT |
| LPIN2 | -0.90499 | 5.077907 | -10.3568 | 3.08E-23 | 1.32E-22 | 41.26631 | NOT |
| ARSD | -0.90889 | 5.454786 | -10.4658 | 1.17E-23 | 5.16E-23 | 42.22821 | NOT |
| GLS | -0.90934 | 6.375863 | -10.0456 | 4.73E-22 | 1.86E-21 | 38.55915 | NOT |
| LPCAT2 | -0.91452 | 3.666602 | -8.45974 | 2.05E-16 | 6.02E-16 | 25.71867 | NOT |
| PDXK | -0.91983 | 5.058809 | -11.1354 | 2.61E-26 | 1.36E-25 | 48.28556 | NOT |
| IDI1 | -0.92092 | 4.858366 | -11.109 | 3.33E-26 | 1.70E-25 | 48.04264 | NOT |
| FDFT1 | -0.92339 | 5.252419 | -12.1217 | 2.10E-30 | 1.45E-29 | 57.64931 | NOT |
| LPL | -0.92394 | 4.413167 | -6.06706 | 2.31E-09 | 4.81E-09 | 9.763902 | NOT |
| DDC | -0.9244 | 5.268513 | -3.38331 | 0.000762828 | 0.001108469 | -2.43799 | NOT |
| CYCS | -0.92706 | 6.169829 | -10.2074 | 1.15E-22 | 4.72E-22 | 39.9596 | NOT |
| SPHK2 | -0.92828 | 2.924912 | -14.7276 | 3.90E-42 | 4.25E-41 | 84.52693 | NOT |
| ASRGL1 | -0.93293 | 4.278852 | -6.16628 | 1.28E-09 | 2.71E-09 | 10.33559 | NOT |
| CYP51A1 | -0.9339 | 1.817938 | -10.2035 | 1.19E-22 | 4.87E-22 | 39.92561 | NOT |
| ACOT2 | -0.9349 | 4.433481 | -10.7554 | 8.59E-25 | 4.07E-24 | 44.8173 | NOT |
| DLD | -0.93558 | 5.817944 | -10.8238 | 4.61E-25 | 2.22E-24 | 45.43576 | NOT |
| ACAD8 | -0.93932 | 3.646327 | -13.8263 | 6.06E-38 | 5.90E-37 | 74.91841 | NOT |
| GK | -0.94039 | 3.540545 | -8.05213 | 4.39E-15 | 1.21E-14 | 22.69478 | NOT |
| AFMID | -0.94109 | 4.623391 | -10.409 | 1.94E-23 | 8.39E-23 | 41.72617 | NOT |
| SDHD | -0.94436 | 6.718707 | -11.9039 | 1.76E-29 | 1.14E-28 | 55.53916 | NOT |
| AGL | -0.94688 | 3.896752 | -10.7742 | 7.24E-25 | 3.46E-24 | 44.98675 | NOT |
| CBR1 | -0.94926 | 6.47776 | -12.8585 | 1.35E-33 | 1.09E-32 | 64.9562 | NOT |
| SUOX | -0.95109 | 4.681933 | -15.6097 | 2.36E-46 | 3.29E-45 | 94.20228 | NOT |
| AKR1C1 | -0.95206 | 3.205914 | -5.87551 | 6.99E-09 | 1.40E-08 | 8.683503 | NOT |
| HMGCR | -0.95397 | 4.202511 | -11.3677 | 2.96E-27 | 1.64E-26 | 50.44604 | NOT |
| GLB1 | -0.96159 | 5.420097 | -10.8963 | 2.37E-25 | 1.17E-24 | 46.09395 | NOT |
| NT5C1A | -0.96967 | 0.240638 | -24.87 | 2.23E-94 | 2.45E-92 | 204.5242 | NOT |
| PRPS2 | -0.971 | 5.474673 | -11.8373 | 3.35E-29 | 2.12E-28 | 54.89814 | NOT |
| GADL1 | -0.98095 | 0.201411 | -23.0203 | 1.58E-84 | 1.16E-82 | 181.8797 | NOT |
| CKM | -0.98872 | 0.471406 | -15.0636 | 9.94E-44 | 1.19E-42 | 88.18247 | NOT |
| TST | -0.99857 | 5.780392 | -9.02235 | 2.47E-18 | 8.26E-18 | 30.08359 | NOT |
| MOGAT2 | -0.99921 | 0.21235 | -22.252 | 1.91E-80 | 1.24E-78 | 172.4985 | NOT |
| CRYL1 | -1.00013 | 7.609075 | -8.83261 | 1.12E-17 | 3.58E-17 | 28.58728 | DOWN |
| GPT2 | -1.00044 | 3.375571 | -8.95107 | 4.37E-18 | 1.43E-17 | 29.51862 | DOWN |
| HS3ST3B1 | -1.0027 | 1.846099 | -11.9976 | 7.06E-30 | 4.71E-29 | 56.44422 | DOWN |
| MCCC1 | -1.00458 | 4.720651 | -11.1979 | 1.46E-26 | 7.70E-26 | 48.86395 | DOWN |
| SUCLA2 | -1.00511 | 5.431251 | -10.5287 | 6.66E-24 | 3.03E-23 | 42.78613 | DOWN |
| PHYH | -1.00569 | 5.681696 | -10.1939 | 1.30E-22 | 5.26E-22 | 39.84213 | DOWN |
| ALAS1 | -1.0062 | 5.493187 | -12.5651 | 2.60E-32 | 1.98E-31 | 62.01643 | DOWN |
| CYP1A1 | -1.00685 | 0.327884 | -11.1444 | 2.40E-26 | 1.26E-25 | 48.36875 | DOWN |
| FTCD | -1.01072 | 3.571965 | -3.56548 | 0.00039211 | 0.000580513 | -1.81708 | DOWN |
| HIBCH | -1.01426 | 4.119971 | -10.5206 | 7.16E-24 | 3.24E-23 | 42.714 | DOWN |
| PFKM | -1.01672 | 4.875512 | -10.5317 | 6.48E-24 | 2.96E-23 | 42.81305 | DOWN |
| IDH2 | -1.01862 | 7.594545 | -11.9437 | 1.19E-29 | 7.87E-29 | 55.92245 | DOWN |
| KCNJ11 | -1.03018 | 1.446144 | -9.61247 | 1.92E-20 | 7.21E-20 | 34.88984 | DOWN |
| BCHE | -1.03577 | 1.185806 | -7.28784 | 9.97E-13 | 2.50E-12 | 17.35332 | DOWN |
| AMT | -1.03801 | 3.647541 | -10.1918 | 1.32E-22 | 5.34E-22 | 39.82328 | DOWN |
| HPSE2 | -1.03983 | 0.610489 | -13.3521 | 8.59E-36 | 8.08E-35 | 69.98838 | DOWN |
| FDX1 | -1.04077 | 4.722811 | -16.4162 | 2.69E-50 | 4.55E-49 | 103.2531 | DOWN |
| HIBADH | -1.05109 | 7.06379 | -12.0047 | 6.59E-30 | 4.42E-29 | 56.5127 | DOWN |
| ENPP1 | -1.0524 | 2.206305 | -9.30064 | 2.57E-19 | 8.98E-19 | 32.32177 | DOWN |
| HS6ST3 | -1.05309 | 0.626716 | -9.46979 | 6.34E-20 | 2.29E-19 | 33.70701 | DOWN |
| ADK | -1.05318 | 4.209819 | -15.8782 | 1.17E-47 | 1.79E-46 | 97.19502 | DOWN |
| ADHFE1 | -1.05461 | 2.813364 | -11.1149 | 3.15E-26 | 1.63E-25 | 48.09698 | DOWN |
| GOT2 | -1.06326 | 6.411896 | -15.5221 | 6.27E-46 | 8.31E-45 | 93.23018 | DOWN |
| SLC25A15 | -1.06931 | 2.851377 | -11.6247 | 2.58E-28 | 1.52E-27 | 52.86857 | DOWN |
| ECHS1 | -1.07465 | 8.23825 | -14.95 | 3.45E-43 | 3.96E-42 | 86.94231 | DOWN |
| ST3GAL6 | -1.08474 | 2.706934 | -10.5395 | 6.04E-24 | 2.77E-23 | 42.88173 | DOWN |
| PNMT | -1.08612 | 0.435829 | -15.4942 | 8.54E-46 | 1.12E-44 | 92.92168 | DOWN |
| PDHB | -1.09408 | 5.067329 | -17.9942 | 3.23E-58 | 6.97E-57 | 121.4349 | DOWN |
| UQCRC1 | -1.09513 | 6.96532 | -15.3942 | 2.59E-45 | 3.32E-44 | 91.81536 | DOWN |
| NDUFA4 | -1.09666 | 6.619269 | -14.0705 | 4.57E-39 | 4.61E-38 | 77.49249 | DOWN |
| GCAT | -1.10462 | 3.629452 | -9.89701 | 1.71E-21 | 6.59E-21 | 37.28708 | DOWN |
| HYAL1 | -1.10706 | 5.191068 | -10.2085 | 1.14E-22 | 4.70E-22 | 39.96881 | DOWN |
| PDHA1 | -1.10707 | 5.827031 | -16.5313 | 7.24E-51 | 1.26E-49 | 104.5592 | DOWN |
| GOT1 | -1.12193 | 6.933694 | -10.5527 | 5.37E-24 | 2.47E-23 | 42.9994 | DOWN |
| HSD11B1 | -1.12556 | 2.015803 | -7.13167 | 2.86E-12 | 6.94E-12 | 16.31638 | DOWN |
| ENTPD8 | -1.12705 | 1.158284 | -11.8341 | 3.45E-29 | 2.17E-28 | 54.8672 | DOWN |
| OAT | -1.13237 | 5.610422 | -12.8321 | 1.77E-33 | 1.42E-32 | 64.69026 | DOWN |
| GPHN | -1.13282 | 3.463056 | -10.7472 | 9.26E-25 | 4.37E-24 | 44.74296 | DOWN |
| ACSL6 | -1.13435 | 0.64669 | -18.0851 | 1.11E-58 | 2.50E-57 | 122.4981 | DOWN |
| SLC25A4 | -1.13909 | 5.268141 | -12.2165 | 8.27E-31 | 5.95E-30 | 58.57572 | DOWN |
| PC | -1.13983 | 3.907244 | -8.85359 | 9.51E-18 | 3.06E-17 | 28.75155 | DOWN |
| CROT | -1.14619 | 3.676379 | -15.7137 | 7.40E-47 | 1.10E-45 | 95.35933 | DOWN |
| NDUFS1 | -1.14783 | 4.899881 | -13.27 | 2.01E-35 | 1.82E-34 | 69.14459 | DOWN |
| ALDH1L1 | -1.15849 | 4.090985 | -5.72754 | 1.61E-08 | 3.15E-08 | 7.870032 | DOWN |
| CYP8B1 | -1.15867 | 2.188905 | -6.49256 | 1.77E-10 | 3.89E-10 | 12.27289 | DOWN |
| AUH | -1.1608 | 5.0881 | -12.1729 | 1.27E-30 | 8.96E-30 | 58.14874 | DOWN |
| PTGS2 | -1.18636 | 2.354666 | -6.9703 | 8.36E-12 | 1.98E-11 | 15.26489 | DOWN |
| TM7SF2 | -1.19416 | 4.035026 | -11.3189 | 4.69E-27 | 2.55E-26 | 49.9896 | DOWN |
| ACOX2 | -1.19958 | 3.389097 | -9.50031 | 4.92E-20 | 1.80E-19 | 33.95891 | DOWN |
| AK7 | -1.20861 | 1.943829 | -12.933 | 6.34E-34 | 5.17E-33 | 65.70887 | DOWN |
| L2HGDH | -1.22298 | 2.713473 | -12.0624 | 3.76E-30 | 2.55E-29 | 57.07227 | DOWN |
| ACAA1 | -1.22851 | 4.587535 | -17.6314 | 2.24E-56 | 4.41E-55 | 117.2055 | DOWN |
| SUCLG2 | -1.22926 | 6.181175 | -15.0645 | 9.84E-44 | 1.19E-42 | 88.19311 | DOWN |
| CHDH | -1.22955 | 4.332845 | -8.82329 | 1.21E-17 | 3.82E-17 | 28.51442 | DOWN |
| ST3GAL4 | -1.23165 | 4.17151 | -11.203 | 1.39E-26 | 7.38E-26 | 48.91177 | DOWN |
| PCYT2 | -1.23196 | 3.643396 | -15.7923 | 3.07E-47 | 4.63E-46 | 96.2357 | DOWN |
| GYG2 | -1.23822 | 1.058147 | -9.72434 | 7.46E-21 | 2.83E-20 | 35.82623 | DOWN |
| GPX2 | -1.25808 | 1.111121 | -10.9377 | 1.62E-25 | 8.09E-25 | 46.47146 | DOWN |
| UQCRFS1 | -1.26081 | 5.650187 | -15.0103 | 1.78E-43 | 2.11E-42 | 87.60032 | DOWN |
| SGPP1 | -1.26101 | 5.028744 | -14.1242 | 2.58E-39 | 2.63E-38 | 78.06119 | DOWN |
| BCKDHB | -1.26364 | 3.877011 | -13.6393 | 4.32E-37 | 4.10E-36 | 72.96335 | DOWN |
| CAT | -1.26984 | 7.32477 | -11.7483 | 7.90E-29 | 4.88E-28 | 54.04567 | DOWN |
| SLC25A5 | -1.29341 | 8.679234 | -12.6925 | 7.23E-33 | 5.64E-32 | 63.28835 | DOWN |
| PTGR1 | -1.29549 | 6.021131 | -12.334 | 2.59E-31 | 1.89E-30 | 59.729 | DOWN |
| AOX1 | -1.29741 | 4.728373 | -5.61475 | 3.01E-08 | 5.81E-08 | 7.262462 | DOWN |
| LIPG | -1.31376 | 1.64642 | -10.1815 | 1.45E-22 | 5.80E-22 | 39.73371 | DOWN |
| CYP4A22 | -1.3269 | 2.085959 | -5.73732 | 1.53E-08 | 2.99E-08 | 7.923192 | DOWN |
| EPHX2 | -1.34252 | 5.467404 | -10.486 | 9.75E-24 | 4.34E-23 | 42.40729 | DOWN |
| PLA2G4A | -1.34337 | 2.818875 | -10.9961 | 9.47E-26 | 4.80E-25 | 47.00522 | DOWN |
| PIP4K2C | -1.34417 | 5.423718 | -18.7962 | 2.52E-62 | 6.75E-61 | 130.8703 | DOWN |
| ACSM3 | -1.35015 | 3.571336 | -11.6298 | 2.46E-28 | 1.45E-27 | 52.91728 | DOWN |
| ALDH3B2 | -1.35745 | 0.294787 | -27.5894 | 7.94E-109 | 1.75E-106 | 237.7362 | DOWN |
| ITPR2 | -1.36328 | 3.674649 | -11.8491 | 2.99E-29 | 1.91E-28 | 55.01181 | DOWN |
| PCK2 | -1.37539 | 5.138472 | -11.6179 | 2.76E-28 | 1.61E-27 | 52.8038 | DOWN |
| DBT | -1.37743 | 3.422825 | -18.3366 | 5.77E-60 | 1.41E-58 | 125.449 | DOWN |
| ACAT1 | -1.38803 | 6.278877 | -10.4323 | 1.58E-23 | 6.85E-23 | 41.93123 | DOWN |
| PCCA | -1.40277 | 4.560863 | -12.8 | 2.44E-33 | 1.93E-32 | 64.36782 | DOWN |
| CKB | -1.40464 | 6.527842 | -8.46174 | 2.02E-16 | 5.95E-16 | 25.73382 | DOWN |
| NNT | -1.41075 | 5.165884 | -12.1112 | 2.33E-30 | 1.60E-29 | 57.54693 | DOWN |
| AGMAT | -1.41152 | 4.973177 | -6.37343 | 3.69E-10 | 8.00E-10 | 11.5554 | DOWN |
| SLC19A2 | -1.41172 | 3.510602 | -11.6411 | 2.21E-28 | 1.31E-27 | 53.02414 | DOWN |
| QPRT | -1.41326 | 5.428636 | -9.07532 | 1.61E-18 | 5.44E-18 | 30.50563 | DOWN |
| ACSL4 | -1.41528 | 5.443436 | -15.3227 | 5.72E-45 | 7.15E-44 | 91.02702 | DOWN |
| PTGES | -1.43635 | 3.128208 | -7.70323 | 5.51E-14 | 1.46E-13 | 20.20221 | DOWN |
| GATM | -1.44112 | 6.926292 | -7.08665 | 3.87E-12 | 9.27E-12 | 16.021 | DOWN |
| ACADM | -1.45281 | 5.734655 | -12.0131 | 6.07E-30 | 4.10E-29 | 56.59425 | DOWN |
| PANK1 | -1.4541 | 3.223043 | -10.9375 | 1.63E-25 | 8.09E-25 | 46.46949 | DOWN |
| MBOAT2 | -1.46124 | 3.012369 | -14.7939 | 1.90E-42 | 2.09E-41 | 85.24544 | DOWN |
| CDS1 | -1.48692 | 4.097921 | -12.1806 | 1.18E-30 | 8.37E-30 | 58.22381 | DOWN |
| CACNA2D2 | -1.48779 | 1.153628 | -24.6368 | 3.89E-93 | 3.89E-91 | 201.6679 | DOWN |
| CDO1 | -1.51406 | 1.201599 | -14.3517 | 2.26E-40 | 2.39E-39 | 80.48392 | DOWN |
| UST | -1.52526 | 1.970245 | -11.3088 | 5.15E-27 | 2.79E-26 | 49.89568 | DOWN |
| LDHB | -1.52675 | 8.929128 | -17.764 | 4.78E-57 | 9.81E-56 | 118.7483 | DOWN |
| ETNK2 | -1.53738 | 4.217143 | -10.4465 | 1.39E-23 | 6.11E-23 | 42.05667 | DOWN |
| PIP5K1B | -1.54679 | 2.002665 | -14.4913 | 5.04E-41 | 5.43E-40 | 81.9799 | DOWN |
| ENTPD3 | -1.55338 | 0.508156 | -27.2464 | 5.19E-107 | 9.52E-105 | 233.5628 | DOWN |
| SUCLG1 | -1.56381 | 6.040636 | -21.1298 | 1.66E-74 | 7.60E-73 | 158.8538 | DOWN |
| CTH | -1.59593 | 2.602459 | -18.7681 | 3.51E-62 | 9.19E-61 | 130.5379 | DOWN |
| HADH | -1.61066 | 5.743064 | -19.9631 | 2.21E-68 | 8.38E-67 | 144.7815 | DOWN |
| SORD | -1.61424 | 3.440455 | -13.9682 | 1.35E-38 | 1.34E-37 | 76.41175 | DOWN |
| MTTP | -1.64554 | 0.97159 | -11.386 | 2.49E-27 | 1.38E-26 | 50.61701 | DOWN |
| AGXT | -1.6456 | 1.031944 | -14.4689 | 6.42E-41 | 6.86E-40 | 81.7387 | DOWN |
| TREH | -1.65413 | 1.68362 | -10.9929 | 9.76E-26 | 4.92E-25 | 46.97582 | DOWN |
| CYP4F3 | -1.65728 | 1.52659 | -9.31532 | 2.28E-19 | 8.01E-19 | 32.44129 | DOWN |
| PCCB | -1.66367 | 3.774129 | -18.9433 | 4.38E-63 | 1.27E-61 | 132.6135 | DOWN |
| SLC22A13 | -1.66906 | 1.280061 | -10.8633 | 3.21E-25 | 1.57E-24 | 45.79376 | DOWN |
| CYP4A11 | -1.67672 | 4.252433 | -4.81019 | 1.91E-06 | 3.28E-06 | 3.248323 | DOWN |
| GSTO2 | -1.67704 | 1.720748 | -14.2204 | 9.24E-40 | 9.50E-39 | 79.08316 | DOWN |
| CYP1B1 | -1.71996 | 4.311024 | -9.30224 | 2.54E-19 | 8.89E-19 | 32.33478 | DOWN |
| CYP39A1 | -1.72059 | 2.114812 | -13.0328 | 2.29E-34 | 1.96E-33 | 66.72098 | DOWN |
| GPD1L | -1.75811 | 4.253419 | -16.1944 | 3.32E-49 | 5.37E-48 | 100.7463 | DOWN |
| ACOT12 | -1.76908 | 0.345336 | -39.0582 | 5.87E-167 | 1.62E-164 | 371.3491 | DOWN |
| PHGDH | -1.77208 | 3.906271 | -10.0429 | 4.84E-22 | 1.90E-21 | 38.53547 | DOWN |
| ACOT11 | -1.81455 | 2.041882 | -19.4545 | 9.87E-66 | 3.39E-64 | 138.6941 | DOWN |
| ACADSB | -1.82254 | 4.744847 | -15.5637 | 3.94E-46 | 5.42E-45 | 93.69201 | DOWN |
| HS6ST1 | -1.84687 | 4.6944 | -18.9334 | 4.93E-63 | 1.36E-61 | 132.496 | DOWN |
| ALDH4A1 | -1.85814 | 5.423007 | -11.5053 | 8.06E-28 | 4.55E-27 | 51.73816 | DOWN |
| UGT8 | -1.87197 | 4.35331 | -13.2012 | 4.08E-35 | 3.62E-34 | 68.43868 | DOWN |
| SLC5A3 | -1.89803 | 4.748894 | -12.3346 | 2.58E-31 | 1.89E-30 | 59.73427 | DOWN |
| CYP24A1 | -1.90113 | 1.869958 | -8.59434 | 7.26E-17 | 2.18E-16 | 26.74306 | DOWN |
| HAO2 | -1.90749 | 4.380029 | -6.82591 | 2.14E-11 | 4.96E-11 | 14.34142 | DOWN |
| DCXR | -1.91009 | 5.53547 | -19.6273 | 1.25E-66 | 4.42E-65 | 140.7593 | DOWN |
| PIPOX | -1.91649 | 3.174011 | -9.74613 | 6.20E-21 | 2.36E-20 | 36.0096 | DOWN |
| ASS1 | -1.93349 | 7.110172 | -12.1322 | 1.90E-30 | 1.33E-29 | 57.7514 | DOWN |
| CYP27B1 | -1.94374 | 1.308306 | -16.3005 | 9.99E-50 | 1.67E-48 | 101.9435 | DOWN |
| BDH1 | -1.96391 | 1.723637 | -16.4873 | 1.19E-50 | 2.05E-49 | 104.0604 | DOWN |
| OXCT1 | -1.99493 | 4.591128 | -13.0255 | 2.47E-34 | 2.09E-33 | 66.64697 | DOWN |
| PFKFB2 | -2.02202 | 2.750874 | -17.2736 | 1.43E-54 | 2.62E-53 | 113.0626 | DOWN |
| CKMT2 | -2.04781 | 2.353263 | -9.63397 | 1.60E-20 | 6.04E-20 | 35.06914 | DOWN |
| DAO | -2.11718 | 1.965776 | -11.2903 | 6.13E-27 | 3.31E-26 | 49.72272 | DOWN |
| PTGDS | -2.13675 | 3.895385 | -9.6006 | 2.12E-20 | 7.91E-20 | 34.7909 | DOWN |
| FBP1 | -2.14774 | 6.081365 | -12.6212 | 1.48E-32 | 1.14E-31 | 62.57602 | DOWN |
| DEGS2 | -2.15586 | 1.773697 | -17.5684 | 4.67E-56 | 8.86E-55 | 116.4744 | DOWN |
| HPGD | -2.31674 | 2.639741 | -13.7881 | 9.07E-38 | 8.75E-37 | 74.51785 | DOWN |
| IYD | -2.33596 | 0.895408 | -21.9263 | 1.02E-78 | 5.61E-77 | 168.5295 | DOWN |
| ACSF2 | -2.47693 | 3.291703 | -20.2349 | 8.38E-70 | 3.41E-68 | 148.0471 | DOWN |
| GMPR | -2.47736 | 3.395498 | -19.1656 | 3.11E-64 | 9.50E-63 | 135.2527 | DOWN |
| SCD5 | -2.47836 | 4.250894 | -12.4649 | 7.06E-32 | 5.32E-31 | 61.02195 | DOWN |
| GLDC | -2.50184 | 3.274948 | -15.4044 | 2.32E-45 | 3.00E-44 | 91.92817 | DOWN |
| G6PC | -2.51613 | 2.290083 | -10.2333 | 9.17E-23 | 3.79E-22 | 40.18502 | DOWN |
| CEL | -2.58916 | 0.946218 | -26.5168 | 3.85E-103 | 4.70E-101 | 224.6666 | DOWN |
| PAH | -2.60981 | 2.528473 | -9.48368 | 5.65E-20 | 2.04E-19 | 33.82157 | DOWN |
| SLC44A4 | -2.62401 | 4.023825 | -12.933 | 6.34E-34 | 5.17E-33 | 65.70957 | DOWN |
| SLC5A2 | -2.6265 | 0.979551 | -19.1686 | 3.00E-64 | 9.43E-63 | 135.2884 | DOWN |
| ALDH6A1 | -2.63606 | 4.912182 | -17.7731 | 4.30E-57 | 9.09E-56 | 118.8541 | DOWN |
| ARG2 | -2.66117 | 4.056055 | -12.6781 | 8.36E-33 | 6.48E-32 | 63.14405 | DOWN |
| UPP2 | -2.66414 | 1.252948 | -21.3598 | 1.01E-75 | 4.85E-74 | 161.6427 | DOWN |
| ENPP6 | -2.76693 | 0.922619 | -26.5741 | 1.91E-103 | 3.00E-101 | 225.3661 | DOWN |
| CYP4F2 | -2.87727 | 0.936113 | -18.0936 | 1.01E-58 | 2.30E-57 | 122.5983 | DOWN |
| PCK1 | -3.03974 | 4.895875 | -10.294 | 5.37E-23 | 2.26E-22 | 40.71498 | DOWN |
| CYP17A1 | -3.04129 | 1.457221 | -17.5984 | 3.29E-56 | 6.36E-55 | 116.823 | DOWN |
| PIK3C2G | -3.11343 | 0.541337 | -40.2548 | 1.18E-172 | 4.33E-170 | 384.4371 | DOWN |
| TYRP1 | -3.385 | 0.618497 | -40.7162 | 7.89E-175 | 4.34E-172 | 389.4355 | DOWN |
| LIPH | -3.43607 | 1.524602 | -23.8414 | 6.73E-89 | 6.17E-87 | 191.9276 | DOWN |
| DIO1 | -3.603 | 1.444995 | -21.1197 | 1.87E-74 | 8.25E-73 | 158.7314 | DOWN |
| PLA2G4F | -3.74566 | 0.851851 | -26.5372 | 3.00E-103 | 4.12E-101 | 224.9154 | DOWN |
| DPEP1 | -3.77193 | 2.948938 | -15.525 | 6.06E-46 | 8.14E-45 | 93.26298 | DOWN |
| INPP5J | -3.81275 | 1.669559 | -23.3898 | 1.71E-86 | 1.34E-84 | 186.3994 | DOWN |
| HSD11B2 | -3.84919 | 5.713457 | -19.4218 | 1.46E-65 | 4.72E-64 | 138.3048 | DOWN |
| HMGCS2 | -3.98941 | 4.237237 | -13.145 | 7.26E-35 | 6.31E-34 | 67.86449 | DOWN |
| HPD | -4.19934 | 2.736985 | -17.1457 | 6.28E-54 | 1.13E-52 | 111.5879 | DOWN |
| HS6ST2 | -4.40824 | 0.84687 | -52.1127 | 6.23E-225 | 6.85E-222 | 504.4724 | DOWN |
| ALDOB | -4.94515 | 5.476194 | -13.2893 | 1.64E-35 | 1.51E-34 | 69.34284 | DOWN |
