# Supplementary material for: Metabolic Understanding of the Genetic Dysregulation in the Tumor Microenvironment of Kidney Renal Clear Cell Carcinoma
Source: Dis Markers. 2022 Jan 20;2022:6085072. doi: 10.1155/2022/6085072 (PMC8794690; doi:10.1155/2022/6085072)
Supplement: Supplementary Materials — Supplementary Figure 1: graphical abstract of this study. Supplementary Figure 2: the detailed workflow of data acquisition and downstream process study. Supplementary Figure 3: GSEA enrichment analysis of metabolic genes in tumor and normal tissue. Supplementary Figure 4: PPI network of metabolic genes. (a) PPI network of up- and downregulated metabolic genes. (b) Top 10 hub genes of PPI. Supplementary Figure 5: screening the survival-related genes. (a) Partial likelihood deviance of DEGs identified by LASSO regression model. (b) LASSO coefficients of DEGs. (c) Forrest plot showing multivariate analysis of selected genes identified by LASSO regression model. Supplementary Figure 6: correlation of NUDTI expression with immune infiltration level in KIRC. Supplementary Table 1: primers used in qPCR analysis and the small RNA interfering assay. Supplementary Table 2: the 1100 differentially expressed metabolic genes in tumor vs normal tissues. Supplementary Table 3: correlation between hub gene expression and clinicopathological features of KIRC in TCGA datasets. [file 6085072.f1.zip › Supplementary table 1.doc]

**Supplementary table 1: Primers used in qPCR analysis and the small RNA interfering assay.**

| Name | Sequence (5′-3′) |
| --- | --- |
| NUDT1F | AGTGCAAGAAGGAGAGACCATC |
| NUDT1R | CCCACGAACTCAAACACGATC |
| Si-NUDT1-1F | UCAUGGACGUGCAUGUCUUTT |
| Si-NUDT1-1R | AAGACAUGCACGUCCAUGATT |
| Si-NUDT1-2F | GGUUCCAGCUGGAUCAGAUTT |
| Si-NUDT1-2R | AUCUGAUCCAGCUGGAACCTT |
